# Antinutritional factors in pearl millet grains: Phytate and goitrogens content variability and molecular characterization of genes involved in their pathways

**DOI:** 10.1371/journal.pone.0198394

**Published:** 2018-06-01

**Authors:** Eleonora Boncompagni, Gregorio Orozco-Arroyo, Eleonora Cominelli, Prakash Irappa Gangashetty, Stefania Grando, Theophilus Tenutse Kwaku Zu, Maria Gloria Daminati, Erik Nielsen, Francesca Sparvoli

**Affiliations:** 1 Department of Biology and Biotechnology, University of Pavia, Pavia, Italy; 2 Institute of Agricultural Biology and Biotechnology, CNR, Milan, Italy; 3 ICRISAT Sahelian Center, International Crops Research Institute for the Semi-Arid Tropics, Niamey, Niger; 4 ICRISAT Patancheru, International Crops Research Institute for the Semi-Arid Tropics, Andhra Pradesh, India; Ben-Gurion University, ISRAEL

## Abstract

Pearl millet [*Pennisetum glaucum* (L.) R. Br.] is an important “orphan” cereal and the most widely grown of all the millet species worldwide. It is also the sixth most important cereal in the world after wheat, rice, maize, barley, and sorghum, being largely grown and used in West Africa as well as in India and Pakistan. The present study was carried out in the frame of a program designed to increase benefits and reduce potential health problems deriving from the consumption of pearl millet. The specific goal was to provide a database of information on the variability existing in pearl millet germplasm as to the amounts of phytate, the most relevant antinutrient compound, and the goitrogenic compounds C-glycosylflavones (C-GFs) accumulated in the grain.Results we obtained clearly show that, as indicated by the range in values, a substantial variability subsists across the investigated pearl millet inbred lines as regards the grain level of phytic acid phosphate, while the amount of C-GFs shows a very high variation. Suitable potential parents to be used in breeding programs can be therefore chosen from the surveyed material in order to create new germplasm with increased nutritional quality and food safety. Moreover, we report novel molecular data showing which genes are more relevant for phytic acid biosynthesis in the seeds as well as a preliminary analysis of a pearl millet orthologous gene for C-GFs biosynthesis. These results open the way to dissect the genetic determinants controlling key seed nutritional phenotypes and to the characterization of their impact on grain nutritional value in pearl millet.

## Introduction

Pearl millet [*Pennisetum glaucum* (L.) R. Br.] is the sixth most important cereal in the world and is the most widely grown among all the millet species worldwide, followed by foxtail millet (*Setaria italica*), proso millet (*Panicum miliaceum*) and finger millet (*Eleusine coracana*) [1].

Pearl millet is an important cereal in arid and semi-arid regions both in India and sub-Saharan Africa where it contributes to food security, but it also has other uses, such as source of feed and fodder for livestock, fuel and construction materials [2,3]. It is mostly grown in areas with limited agronomic potential characterised by low rainfall (300–500 mm) and marginal soils. These aspects make millet an important food staple all over the African continent, especially in the semi-arid areas of the Western Sahel where other crops tend to fail because inadequate rainfall and poor soil conditions.

Overall, pearl millet thanks to high levels of metabolizable energy and protein, a balanced amino acid profile, and a rich source of fibres and lipids, is nutritionally superior to many others cereals [4–6]. Moreover, pearl millet food is gluten free, has a low glycemic index, and is a significant source of micronutrients such as iron and zinc (contents are higher than those in other cereals), both in India and sub-Saharan Africa where, as compared to other cereals and vegetables, it potentially represents one of the cheapest source of these micronutrients [7].

Malnutrition arising from dietary deficiency of one or more essential micronutrients affects two thirds of the world's population [8]. The mineral elements most commonly lacking in human diets are iron (Fe) and zinc (Zn), which rank fifth and sixth, respectively, among the top 10 risk factors contributing to burden of disease, especially in the developing countries [9]. Crop biofortification is a sustainable and cost-effective approach to address micronutrient malnutrition, especially in the developing world [10, 11]. Research carried out at International Crop Research Institute for Semi-Arid Tropics (ICRISAT) showed high levels (much higher than those of other cereals) and large variability for both iron and zinc [12–15]. Commercial open-pollinated varieties and experimental hybrids of pearl millet with high iron content (68–72 ppm) were developed [12,16–18] and the line ICTP 8203Fe, with 71 ppm of iron density and 2.21 t/ha of grain yield, was the first biofortified crop cultivar officially released (in 2013) and reaching farmers’ fields in India [13,19].

However, like in other cereals, biofortification in millet is still limited by the presence of antinutrients like phytic acid, polyphenols, and tannins. Phytate is the salt of phytic acid, *myo*-inositol-1,2,3,4,5,6 hexa*kis*phosphate, it is widely distributed in the plant kingdom and serves as the major form of stored phosphorus and minerals containing up to 75% of total phosphorus in the kernel [20]. Phytic acid has strong chelating ability and readily forms complexes with monovalent and multivalent cations of potassium, calcium, iron, zinc, magnesium and other cations, reducing their bioavailability and creating a deficit in their absorption. Several studies were published reporting the range of natural variability of minerals content in millet grain, but a very limited work was done for phytic acid. The level of phytic acid (as phytate phosphorus) of two millet lines was evaluated and shown to be in the range of 179–306 mg/100 g and to vary according to the location and genotype [21]. Another study [22] analysed the genetics of phytic acid content in pearl millet using a 12x12 complete diallel cross. They showed highly significant differences among parents as well as the hybrids and demonstrated that both additive and non-additive gene effects were significant.

Besides phytic acid, the presence of some goitrogenic polyphenols, the C-glycosylflavones (C-GFs), such as glucosyl vitexin, glucosyl orientin and vitexin [23,24] might be responsible for health problems. C-glycosylflavones are stable to hydrolysis and are biologically active both *in planta* as well as dietary components. Epidemiologic evidence indicated that a diet based on millet as staple food, such as that occurring in rural villages of Africa and Asia, plays a role in the genesis of endemic goitre in these areas. For instance, goitre was found to be more prevalent in rural villages of the Darfur Province in Sudan, where as much as 74% of dietary energy is derived from millet, than in an urban area, where millet provides only 37% of calories, even though the degree of iodine deficiency was similar in the two areas [25–27].

From a dietary perspective, C-GFs are implicated in both beneficial and detrimental biological activities [28,29]. Notwithstanding all their health benefits, some of these phenolic compounds interfere with numerous enzymatic systems in humans, and most notably those that control thyroid hormone synthesis, which is impaired [29,30]. This can lead to the development of goitre, i.e. a swelling of the neck or larynx resulting from enlargement of the thyroid gland. Goitre is a pathology endemic in many poor developing countries and is still prevalent in Central Africa, India, China and Central Asia. Although the great majority of the cases of goitre is due to dietary iodine deficiency, the incidence of this pathology in animals and humans with normal dietary intake of iodine suggests that there are other factors in the etiology of this condition [31]. Prominent among them are goitrogen molecules whose ingestion interferes with iodine in the body on several levels and cause hypothyroidism due to the fact that the gland is no more able to synthesize suitable amounts of thyroid hormones, leading eventually to thyroid enlargement, and goitre formation. Moreover, it was demonstrated that high prevalence of iron deficiency among children in areas of endemic goitre may reduce the effectiveness of iodized salt distribution programs [32]. Therefore, it is strongly recommended to improve iron status also in areas where iodine deficiency is present.

For pearl millet, a first achievement of biofortification was the development of high iron and high zinc lines [13,19]. This notwithstanding, the reduction in the grains of phytic acid and goitrogens still remains challenging. A prerequisite of any breeding approach is to verify how large is the genetic diversity for these traits. Moreover, translational approaches from other crops to characterize genes involved in the biosynthetic pathways for phytic acid and C-GFs may provide useful information on key genes involved in the synthesis and/or accumulation of these compounds.

Phytic acid may be synthesized through two different routes: 1) the lipid-independent pathway that consists in the sequential phosphorylation of the 6-carbon cyclic alcohol *myo*-inositol (Ins) and soluble inositol phosphates (InsPs); and 2) the lipid-dependent pathway that uses precursors that include phosphatidylinositol (PtdIns) and PtdIns phosphates. This last route is present in most eukaryotic cells, including plant vegetative tissues, while the first pathway appears to be predominant in seeds [33] (S1 Fig). Concerning C-glycosylflavones, their production depends on the activity of the key-enzyme C-glycosyl transferase (CGT) belonging to family 1 glycosyltransferases. This family contains most plant uridine diphosphate glycosyltransferases (UGTs) that use UDP-sugar as an activated donor of sugar moieties (the most common is UDP-glucose) that is directly bound to the carbon atom of an aglycon, forming a C–C bond at the anomeric carbon [34] (S2 Fig). A number of CGTs were isolated and their specificity for flavones was shown in different crops such as rice, maize, buckwheat and citrus [35–38]. In particular, a maize CGT (UGT708A6) was isolated and shown to catalyse the addition of a glucose molecule to 2-hydroxyflavanones, generating C-glycosylflavones [36].

With the purpose to contribute to increase the nutritional quality and the food safety of pearl millet as well as to widen knowledge in this area, the present study aimed at analysing the natural variability for the content of phytic acid and C-glycosylflavones in a panel of 145, in the case of phytic acid, and 97, for C-GFs, pearl millet inbred lines covering a large genetic diversity. Furthermore, we identified and characterised key genes involved in phytic acid biosynthetic pathway and a gene coding for a C-glycosyl transferase, a key-enzyme involved in C-glycosyl flavones synthesis. Finally, we compared the expression of these genes during seed development of contrasting lines for phytate and C-glycosylflavones content, and showed that some of these genes may be important for the control of the analysed traits.

## Materials and methods

### Plant materials, growth conditions and flours preparations

A total of 145 pearl millet [*Pennisetum glaucum* (L.) R. Br.] inbred lines originating from Indian, West and Central African landraces and covering a large genetic diversity were used in this study and were provided by the ICRISAT (International Crops Research Institute for the Semi-Arid Tropics) Center of Sadoré, Niger (S1 Table). The inbred line SDEB4L-160P6, was used as reference line for gene expression analysis in plant organs.

Selected pearl millet inbred lines were grown under greenhouse conditions in Milan, Italy, with 28°C/20°C day/night temperature and samples were collected from two different plants of each selected line to provide biological replicates. Tissue samples selected for RNA extraction were collected and immediately frozen in liquid nitrogen and stored at -80°C until use. Part of the material was allowed to complete maturation and collected as mature seeds.

Two grams of millet seeds were ground by ball-milling into fine powder using a Retsch Mixer mill MM 301 (Verder Scientific, Haan, Germany). The seeds were put through two 30-second periods of homogenization at 30Hz/sec at room temperature. The insides of the jars and the surface of the balls used to mill the seeds were covered with a hard layer of zirconium oxide so as to avoid any contact between the steel and the biological material and prevent contamination by metal cations. Seed flours were then used for phytates and C-GFs analysis.

### Phytic acid determination

Phytic acid content was determined using the K-PHYT Phytic Acid (Phytate)/Total Phosphorus kit (Megazyme, Wicklow, Ireland) following manufacturer instructions with slight modifications. Briefly, 100 mg seed flour were added to 2 ml of 0.66 M HCl and mixed by shaking at room temperature overnight; 1.5 ml of the resulting extract was centrifuged 10’ at 16000 g and 0.5 ml of the supernatant were neutralized with 0.5 ml of 0.75 M NaOH. 12.5 μl of the neutralized sample were used for the enzymatic dephosphorylation reaction and subsequent total phosphorous (P_tot_) quantification. In parallel 12.5 μl of the neutralized sample were used to quantify the inorganic phosphate (P_i_) of the sample. After the enzymatic treatment, both total phosphorous and inorganic phosphate samples were used to colorimetrically determine their phosphorous content. Standards were plotted as indicated in the manufacturer booklet. The assay is specific for the measurement of phosphorus released as “available phosphorus” from phytic acid, *myo*-inositol (phosphate)_n_ and monophosphate esters by phytase and alkaline phosphatase. Experiments were performed by triplicate on two biological replicas from each sample.

### C-Glycosylflavones extraction

C-Glycosyl flavones were extracted from pearl millet seed flours. The methanolic extracts from seed flour were prepared by adding methanol (2.5 ml) to 250 mg of flour and using a mechanical stirrer, for 6 h (50°C, 200 rpm). Samples were then centrifuged at 13000 rpm for 10 min, and the supernatant was filtered using NY-25mm 0.45 μm syringe filters. The supernatant was collected and used for HPLC analysis. Care was taken throughout the extraction process to minimize exposure of the extracts to direct light. Before injection into the HPLC system the samples were diluted to a third of their initial concentration.

### RP-HPLC analysis of C-glycosylflavones

HPLC-grade water was obtained from a Milli-Q system (Millipore, MA, USA). Chemical standards (glucosyl vitexin, orientin and vitexin, all primary grade) and HPLC Solvents (Acetonitrile, Methanol and Acetic Acid) were purchased from Sigma Aldrich (St Louis, MO, USA).

HPLC analysis was performed using a Jasco HPLC system (Jasco PU-1580 Pump, LG-1580-04 radiant Unit and a UV-1575 detector), equipped with an auto sampler (AS-1555) and a degasser device to prevent the presence of bubbles in the mobile phases (Jasco Europe, Cremella, LC, Italy). The chromatographic separation was performed using a Kinetex PFP column (250 × 4.6 mm, 5 μm, Phenomenex, Torrance, CA, USA) at room temperature by isocratic elution and with a flow rate of 0.4 ml/min. The mobile phase consisted of acetonitrile 21% in a water solution of acetic acid 1%. The run time was 18 min, followed by 5 min for washing column in pure acetonitrile, and 10 min at the initial condition to ri-equilibrate the column. The injection volume was 10 μl and the signal was recorded at 330 nm. All the solvents were filtered through a 0.45 μm Millipore filter before use and degassed in an ultrasonic bath. The system was controlled and data analysis was performed by Borwin software (JMBS Developments, France).

The calibration curves were constructed plotting peak area versus concentration using the Borwin HPLC software. Four different concentrations of the standards (glucosyl vitexin, vitexin and orientin) were prepared in methanol in the range of 5–50 μg/ml and stored at -20°C until use.

The HPLC method was validated in terms of linearity, limit of detection (LOD), limit of quantitation (LOQ) and precision according to the International Conference on Harmonization (ICH) guidelines and based on [39]. The calibration curves were plotted using chromatogram peak areas on 330 nm against the known concentrations of standard solutions (diluted with MetOH 100%) with the help of the Borwin HPLC software (JMBS Developments, France).

LOD and LOQ were experimentally estimated by injecting a series of diluted solutions with known concentrations until the signal-to-noise ratio for the standards reached a 3:1 ratio for LOD and 10:1 for LOQ. Intraday and interday variations were chosen to determine the precision of the HPLC method. For intraday precision, four different concentrations of standard solutions (5, 10, 25, and 50 μg/ml) were determined in one day and expressed as relative standard deviation (RSD). For interday precision, the RSD of the standards were determined for three consecutive days. Accuracy of the HPLC method was assessed by analysing standard compounds and samples. The peaks for the marker compounds from sample solutions were confirmed by comparing the retention time and UV spectra with the reference standards. The peaks were considered pure when there was a coincidence between the two spectral sections.

### Identification of pearl millet orthologous genes for phytic acid biosynthesis and C-glycosyl transferase

Protein sequences of enzymes involved in phytic acid biosynthesis, namely *myo*-inositol-3-phosphate synthase (MIPS), *myo*-inositol-phosphate monophosphatase (IMP), *myo*-inositol kinase (MIK), inositol 1,4,5-tris-phosphate kinase (IPK2), inositol 1,3,4-triphosphate 5 ⁄ 6-kinase (ITPK), inositol 1,3,4,5,6 penta*kis*phosphate 2-kinase (IPK1) and phytic acid transporter to the vacuole (MRP), from common bean, soybean, Arabidopsis, maize, rice, sorghum, *Brachipodium* and foxtail millet (*Setaria italica*) were retrieved from Phytozome v10.2 [40], under default parameters according to known sequences reported by [41] (S2 Table). Protein sequences of known CGTs were retrieved from NCBI GenBank according to gene/protein ID reported by different authors [35–38,42].

Among all these protein sequences those of maize, rice and Arabidopsis were used to search for pearl millet orthologs using Basic Local Alignment Search Tool protein-protein (BLASTp) search on the pearl millet’s genome [43] with the BioEdit Software [44] (S2 Table, in red colour).

For each protein famliy, selected amino acid sequences (S2 Table) were aligned using CLUSTAL W and then the alignments were used for phylogenetic analysis. Neighbor-Joining trees were built using the Jukes-Cantor model with 10000 replicates for the bootstrap. All the analyses were performed using Geneious^**®**^ R11 v. 10.0.7 software (Biomatters Ltd, Silkeborg, Denmark).

### RNA extraction and qRT-PCR analyses

Total RNA was isolated from stems, leaves, plantlets, flowers at anthesis and seeds at three different developmental stages (Days After Flowering, DAF): early (5–8 DAF), middle (9–14 DAF) and late (15–21 DAF). Stages were defined accordingly to the classification made by [45]. RNA isolation was performed in presence of TRIzol reagent (Invitrogen, ThermoFisher Scientific) following the manufacturer instructions; integrity of the total RNA was evaluated with an agarose gel electrophoresis. Ten μg of total RNA were DNase treated (TURBO DNase, Ambion, ThermoFisher Scientific) to degrade any genomic DNA residues and cDNA synthesis was performed from 2 μg of DNA-free RNA with the High-capacity cDNA Reverse Transcription kit (Applied Biosystems, ThermoFisher Scientific). Reactions were run in a 96-well format plates with the 7300 Real-Time PCR System and 7300 System Software (Applied Biosystems, ThermoFisher Scientific).

Relative quantitative RT-PCR (qRT-PCR) analyses were performed using the comparative cycle threshold method according to manufacturer’s protocol (Applied Biosystems) in three technical replicates for each biological duplicate using the primers reported in S4 Table. Relative values for each transcript were determined after normalization with malate dehydrogenase (MDH) and acyl carrier protein (ACP) [46]. The 2^-ΔΔCT^ method was used to calculate relative expression values.

## Results

### Grain phytic acid and total phosphorous analysis

**Fig 1:**
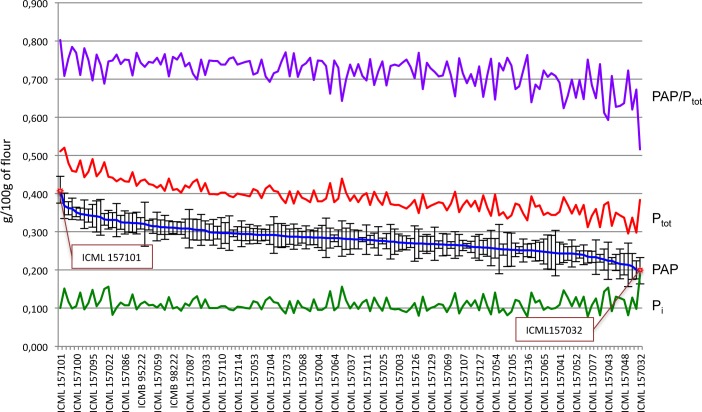
Distribution of phytate and total phosphate among 145 pearl millet lines from a panel of inbred lines covering a large genetic diversity. Phytic acid phosphorus (PAP, blue line), total phosphorus (P_tot_, red line), ratio of phytic acid content to total phosphorus (PAP/P_tot,_, violet line) and P_i_ (green line). Red dots and boxes indicate the most contrasting lines for phytic acid content.

To verify the level of variation of phytic acid content in pearl millet seeds, we determined total phosphorous (P_tot_), inorganic phosphate (P_i_) and phytic acid phosphorous (PAP) content in a panel of 145 pearl millet inbred lines covering a large genetic diversity (Fig 1, S3 Table). We also determined the weight of 50 seeds and the ratio between PAP and P_tot_ (PAP/P_tot_) (S3 Table).

In the analysed population PAP content ranged between 0.198±0.034 and 0.410±0.036 g/100 g of seed flour with a relative standard deviation among the lines of 12.8% and an average PAP content of 0.281 g/100 g (Table 1). Among the population, 57% of the inbred lines analysed presented a PAP content ranging between 0.251–0.300 g/100 g, 22% had PAP content between 0.300–0.350 g/100 g, 17% had PAP values ranging between 0.198–0.250 g/100 g, and only 0.03% of the lines showed values ranging between 0.350–0.401 g/100 g.

**Table 1:** Statistics of phytate contents in pearl millet grains from a panel of inbred lines covering a large genetic diversity.

		Min	Max	Median	Mean	St Dev	Coef var
g/100 g	PAP	0.198	0.410	0.281	0.281	0.036	0.129
	P_tot_	0.296	0.520	0.392	0.389	0.040	0.103
	P_i_	0.077	0.186	0.110	0.105	0.019	0.169
	PAP/P_tot_	0.516	0.803	0.718	0.730	0.043	0.059
g/50 seeds	seed weight	0.163	0.609	0.387	0.373	0.088	0.228

Data are related to phytic acid phosphorus (PAP), total phosphorus (P_tot_), inorganic phosphate (P_i_), ratio of phytic acid phosphorus to total phosphorus (PAP/P_tot_) and seed weight of 145 inbred lines.

As expected there was a strong positive correlation between PAP and P_tot_ and a strong negative correlation between PAP/P_tot_ and P_i_ (Table 2). A moderate correlation existed between PAP/P_tot_ and PAP, while a low correlation was found between P_tot_ and P_i_ (positive) or seed weight (negative). Very little or no correlation was found in the remaining cases (Table 2).

**Table 2:** Linear correlation (Pearson) coefficient between PAP, P_i_, P_tot_, PAP/P_tot_ and seed weight.

Series vs. Series	R	Strenght of the correlation
P_tot_ vs. PAP	0.894**	High
PAP/P_tot_ vs. P_i_	-0.768**	High
PAP/P_tot_ vs. PAP	0.610**	Moderate
P_i_ vs. P_tot_	0.434**	Low
seed weight vs. P_tot_	-0.337**	Low
seed weight vs. P_i_	-0.281**	Little, if any
seed weight vs. PAP	-0.230**	Little, if any
PAP/P_tot_ vs. P_tot_	0.192**	Little, if any
seed weight vs. PAP/P_tot_	0.093	none
P_i_ vs. PAP	-0.003	none

** significant at P< = 0.01

The two most contrasting inbred lines showed a twofold difference in PAP content, with values of 0.198±0.034 g/100 g for the line ICML157032 and 0.410±0.036 g/100 g for the line ICML157101. Hereinafter the two lines are called ICML157032_lpa for low phytic acid content, and ICML157101_hpa for high phytic acid content, respectively. Although the P_tot_ content was 0.384±0.052 g/100 g and 0.511±0.047 g/100 g, respectively for line ICML157032_lpa and line ICML157101_hpa, the PAP/P_tot_ ratios of the two lines were quite different: 0.516±0.058 g/100 g and 0.803±0.047 g/100 g, respectively, indicating that in line ICML157032_lpa the low PAP content was accompanied by an increase in free P_i_ (0.101±0.011 g/100 g and 0.186±0.018 g/100 g, respectively).

**Fig 2:**
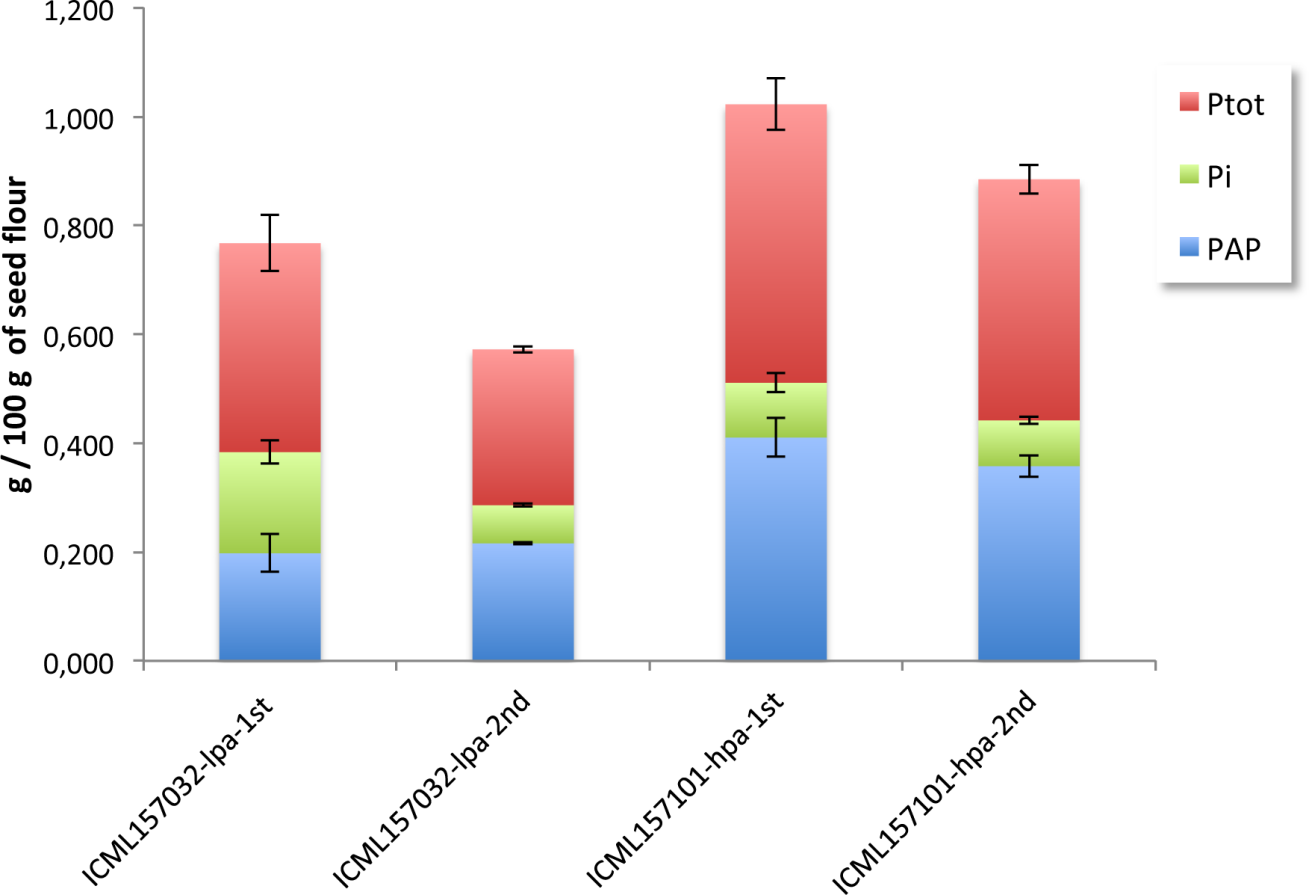
Distribution of phytic acid content, total phosphorus and inorganic phosphate in the grains of the first and second generations of inbred lines contrasting for phytic acid and total C-GFs content. ICML157032, lpa and hcgf and ICML157101, hpa and lcgf. Each data is the mean of two biological replicates.

We next selected and further analysed these two contrasting lines. Under greenhouse conditions, we grew the lines ICML157032_lpa and ICML157101_hpa until mature seed stage. The content of PAP and P_tot_ in mature grains was analysed to verify the contrasting phenotype observed (lpa or hpa). As reported in Fig 2, lpa and hpa phenotypes of the two lines were maintained also in their progenies. The PAP/P_tot_ ratio of the ICML157101_hpa progeny was similar to that of the seeds of the previous generation, whereas for the line ICML157032_lpa the PAP/P_tot_ ratios were 0.516 and 0.755 for the first and second generation, respectively.

### Variability of C-GFs levels in pearl millet grains

**Fig 3:**
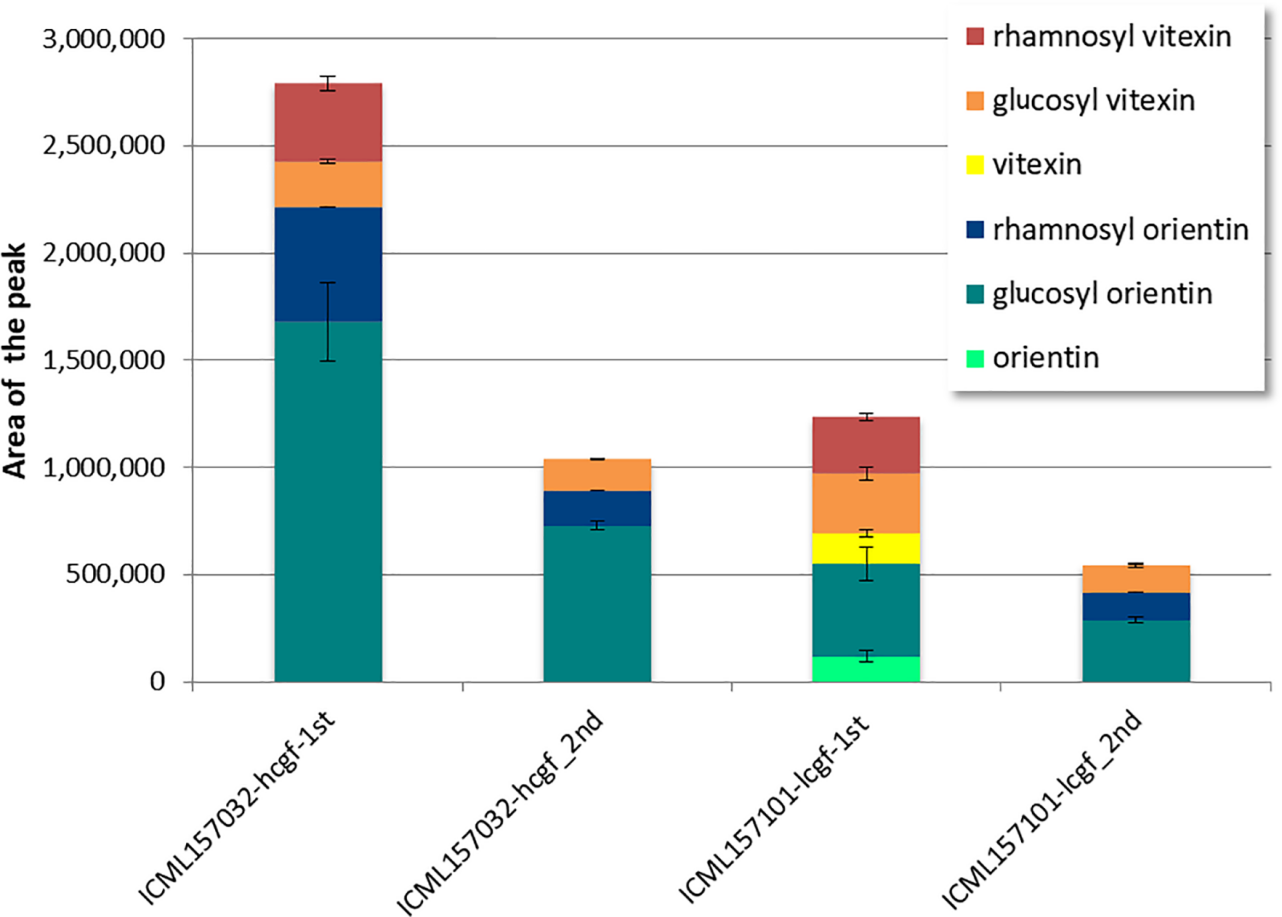
Distribution of C-GFs content among the grains of 96 pearl millet lines from a panel of inbred lines covering a large genetic diversity. Red arrowheads indicate the most contrasting lines, ICML157013 and ICML15711, having the highest and lowest C-GFs content, respectively. Red boxes and arrows indicate the two inbred lines selected for their contrasting content of phytic acid. Each bar is the sum of the content of each C-GF (vitexin, yellow; glucosyl vitexin, orange; rhamnosyl vitexin, dark red; orientin, light green; glucosyl orientin, green; rhamnosyl orientin, blue) in each line.

Available data from literature reported vitexin, glucosyl vitexin and gycosyl orientin as the C-GFs molecules present in pearl millet grains [47]. Since it was not been possible to find on the market pure glucosyl orientin, we quantified and detected vitexin, glucosyl vitexin and orientin in the grains of most of the analysed pearl millet inbred lines and their amount was found to undergo a wide variation across the 97 samples investigated, as shown in Fig 3 (see also S5 Table).

The sum of the amounts of these three C-GFs was recorded to range from 15.29 to 541.10 μg/g flour. The mean content of each compound across all the lines were calculated: orientin was present with the lowest concentration (28.12 μg/g), vitexin content was slightly higher (39.96 μg/g), while glucosyl vitexin showed the highest value (71.85 μg/g). Glucosyl vitexin content ranged from 0 to 283.44 μg/g, while those of vitexin and orientin ranged from 0 to 261.13 μg/g and from 0 to 272.75 μg/g, respectively. Orientin and vitexin were not detectable in 52.1% and 21.9% of the samples, respectively, while glucosyl vitexin was not detected only in one sample (Table 3, S5 Table).

**Table 3:** Statistics of C-GFs contents in pearl millet grains from a panel of inbred lines covering a large genetic diversity. Data refer to C-GFs content in 97 pearl millet lines. Data are expressed as sum of peak area in order to compare data for all the C-GFs, and as μg/g for orientin, vitexin and glucosyl vitexin, for which standards are available.

		Min	Max	Median	Mean	St Dev	Coef var
Peak area Sum	Glucosyl orientin	109377	2670749	388725	486789	355992	0.731
	Glucosyl vitexin	0	1437788	226746	379944	326994	0.861
	Vitexin	0	1416025	182570	230978	232350	1.006
	Orientin	0	1095337	0	117998	201298	1.706
	Rhamnosyl vitexin	0	702528	263.196	253928	156448	0.616
	Rhamnosyl orientin	0	574659	188124	204781	127851	0.624
μg/g	Glucosyl vitexin	0	283.44	42.34	71.85	64.14	0.893
	Orientin	0	272.75	0.00	28.12	49.97	1.777
	Vitexin	0	261.13	31.27	39.96	42.57	1.065

**Fig 4:**
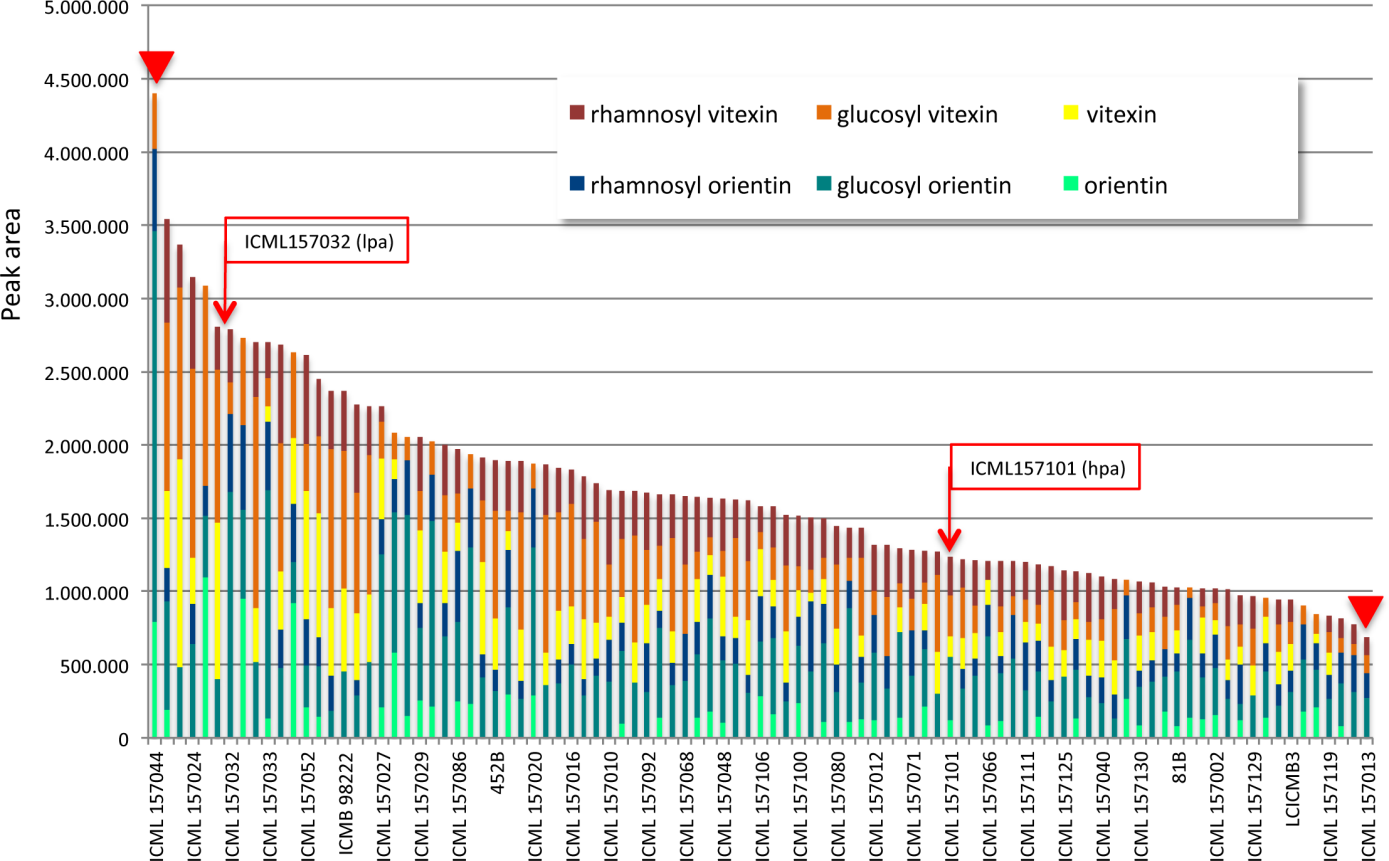
HPLC chromatogram showing the peaks of the different C-GFs identified in pearl millet grains.

Beside these three C-GFs, already known to be present in pearl millet grain, three major additional peaks eluting before or after the peaks of the three above mentioned C-GFs were found to be constantly present in variable amount in most chromatograms of the methanolic extracts of the inbred lines flours (Fig 4). These peaks have been very recently identified by HPLC-MS as corresponding to glucosyl orientin and to other C-GFs molecules previously not reported in pearl millet: rhamnosyl orientin and rhamnosyl vitexin (E. Boncompagni and E. Nielsen, personal communication).

**Fig 5:**
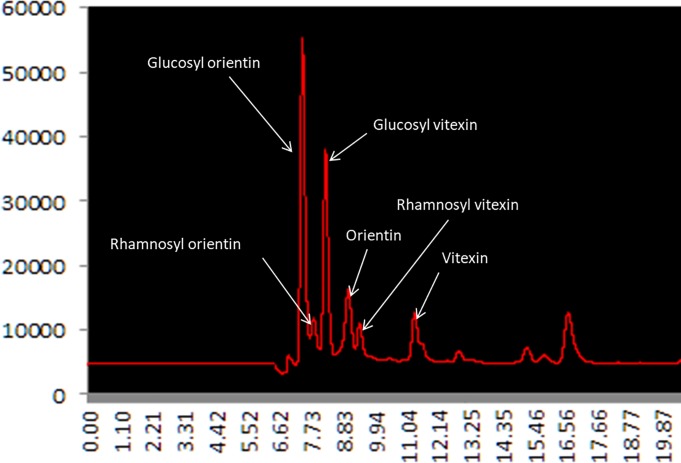
Distribution of the different C-GFs in the grains of the first and second generations of inbred lines contrasting for phytic acid and total C-GFs content. ICML157032, lpa and hcgf and ICML157101, hpa and lcgf. Data are the mean of two biological replicates.

Since pure compounds for these further C-GFs are not commercially available, we could not exactly quantify their amounts in the flours. However, assuming that the extinction coefficients of the six major C-GF molecules found in pearl millet are not too different from each other, we made up a relative comparison across the analysed lines on the basis of the sum of the areas of the six major peaks of the compounds identified as belonging to the C-GF chemical family (Fig 3, S5 Table). Such comparison showed that the most contrasting lines for the total content of C-GFs were lines ICML157044 and ICML157013 having the highest and lowest content, respectively, the line ICML157013 had about 1/5 of total C-GFs content of the line ICML157044 (Fig 3, red arrowheads; S5 Table). The two inbred lines ICML157032_lpa and ICML157101_hpa, already selected for their contrasting content of phytic acid, also showed contrasting C-GFs content, having the line ICML157032_lpa (hereinaf ter called, ICML157032_lpa_hcgf) about 2.5 times more C-GFs than the line ICML157101_hpa (hereinafter called _ ICML157101_hpa_lcgf) (Fig 5).

**Fig 6:**
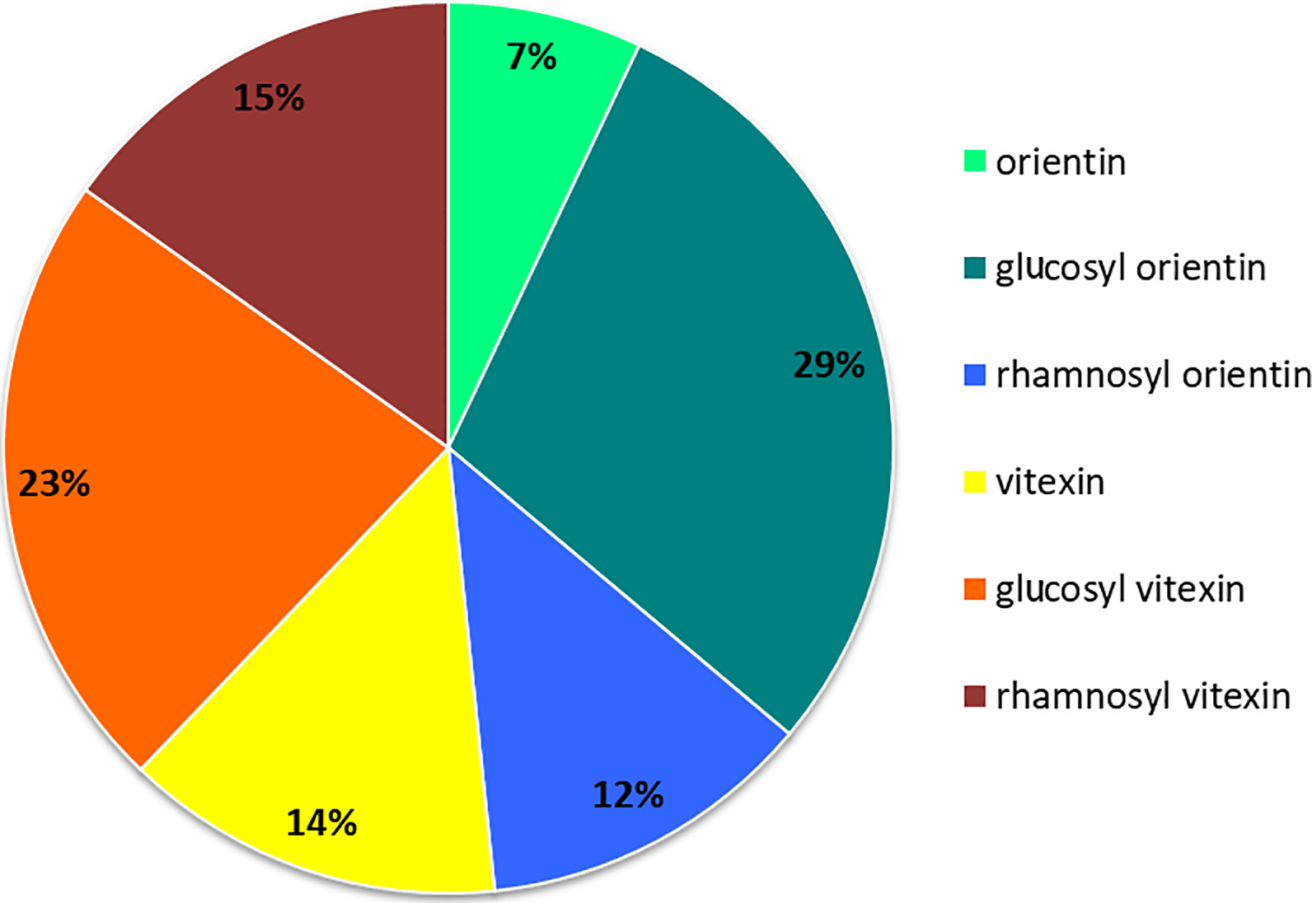
Percentage contribution of each C-GF to the total C-GFs contained in the grains of 96 pearl millet lines from a panel of inbred lines covering a large genetic diversity. Vitexin, yellow; glucosyl vitexin, orange; rhamnosyl vitexin, dark red; orientin, light green; glucosyl orientin, green; rhamnosyl orientin, blue.

Among all the inbred lines, the most abundant compounds were glucosyl orientin and glucosyl vitexin, representing 29% and 23% of total C-GFs, respectively, followed by rhamnosyl vitexin (15%), vitexin (14%) and rhamnosyl orientin (12%) with very similar abundance. Finally, the lowest accumulated C-GF was orientin which represented 7% of total C-GFs (Fig 6).

We verified if any correlation could be found among the relative amounts (measured as chromatographic areas) of C-GFs quantified in the grains of 97 pearl millet inbred lines (Table 4).

**Table 4:** Linear correlation (Pearson) coefficient between each pair of C-GFs.

Series vs. Series	R	Strenght of the correlation
Glucosyl orientin vs. Rhamnosyl orientin	0.525 **	Moderate
Vitexin vs. Glucosyl vitexin	0.523 **	Moderate
Orientin vs. Rhamnosyl orientin	0.463 **	Low
Vitexin vs. Rhamnosyl vitexin	0.446 **	Low
Glucosyl vitexin vs. Rhamnosyl vitexin	0.422 **	Low
Orientin vs. Glucosyl orientin	0.343 **	Low
Vitexin vs. Rhamnosyl orientin	-0.395 **	Low
Glucosyl vitexin vs. Rhamnosyl orientin	-0.298 **	Low
Orientin vs. Rhamnosyl vitexin	-0.462 **	Low
Glucosyl orientin vs. Rhamnosyl vitexin	-0.211 *	Little
Vitexin vs. Glucosyl orientin	-0.204 *	Little
Rhamnosyl vitexin vs. Rhamnosyl orientin	-0.195	none
Vitexin vs. Orientin	-0.177	none
Glucosyl vitexin vs. Glucosyl orientin	-0.056	none
Orientin vs. Glucosyl vitexin	0.026	none

** significant at P< = 0.01

* significant at P< = 0.05

Our results pointed out that significant positive linear correlation (P<0.01) occurs among the different glycosylated forms of orientin as well as among the different glycosylated forms of vitexin, while significant negative linear correlations were found for all couples of C-GFs compounds synthesized in the two different biosynthetic routes starting from orientin or vitexin. The most significant, although with low strength, were vitexin vs rhamnosyl orientin, glucosyl vitexin vs. rhamnosyl orientin and orientin vs. rhamnosyl vitexin. These findings indicate some competitiveness between the two different biosynthetic branches using naringenin as the common precursor and orientin or vitexin as alternative intermediate products.

### Identification of genes involved in phytic acid and C-GFs biosynthesis

To identify key genes involved in phytic acid biosynthesis and accumulation (S1 Fig) we made a BLAST search taking advantage of the availability of the pearl millet genome, that was recently released [41]. We used the deduced amino acid sequences of *MIPS*, *MIK*, *IMP*, *ITPKs*, *IPK2*, *IPK1* and *MRP* coding genes of maize, rice and Arabidopsis as queries (S2 Table).

#### Phytic acid biosynthesis

**Fig 7:**
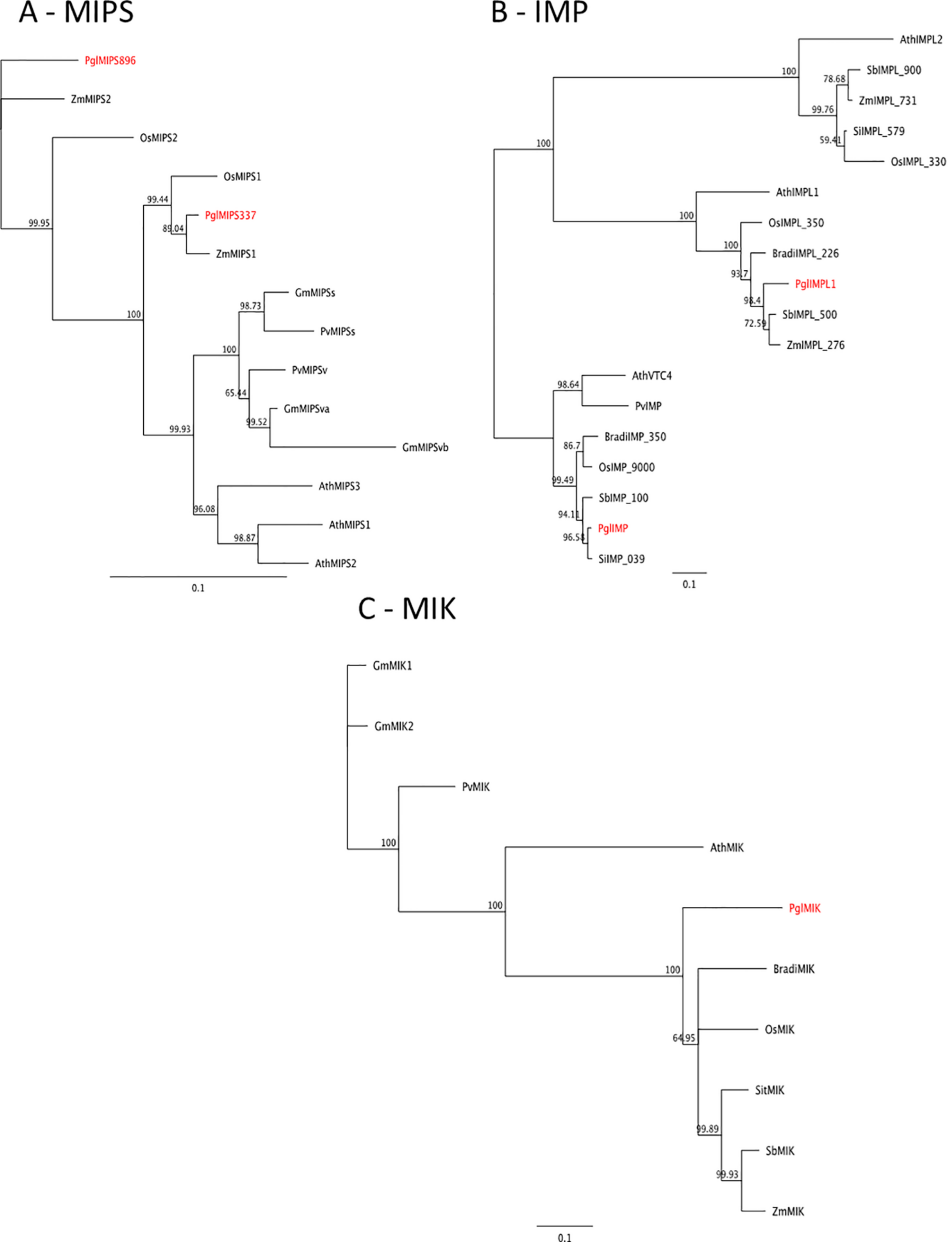
Neighbour-joining trees inferred from the deduced amino acid sequence of early phytic acid pathway genes identified in pearl millet. **(A) MIPS, (B) IMP, (C) MIK**. Bars indicate the number of amino acid substitutions per site. Sequences IDs are reported in S2 Table.

In pearl millet, the *myo*-inositol-1-phosphate synthase (MIPS) is coded by two genes Pgl_GLEAN_10001337 (*PglMIPS_337*) and Pgl_GLEAN_10010896 (*PglMIPS_896*), located on chromosomes 5 and 2, respectively (Fig 7A). The presence of two genes coding for MIPS suggests that they might be differentially expressed in seeds and the other plant tissues, as it occurs in common bean and soybean [48]. *Myo*-inositol-1-phosphate is dephosphorylated to *myo*-inositol by an inositol monophosphatase (IMP). IMP belongs to a small gene family comprising inositol monophosphatase like (*IMPL*) genes, that were well described in Arabidopsis; however, only the IMP protein is involved in the phytic acid biosynthetic pathway [49, 50]. BLAST search showed that pearl millet genome contains one *IMP* orthologue (Pgl_GLEAN_10013074, *PglIMP*) and only one *IMPL* gene (Pgl_GLEAN_10018666, *PglIMPL1*) both located on chromosome 2 (Fig 7B). The reaction catalysed by IMP is reversed by *myo*-inositol kinase (MIK), that in pearl millet is coded by a single copy gene PglGLEAN10008928, *PglMIK*, located on chromosome 2 (Fig 7C).

**Fig 8:**
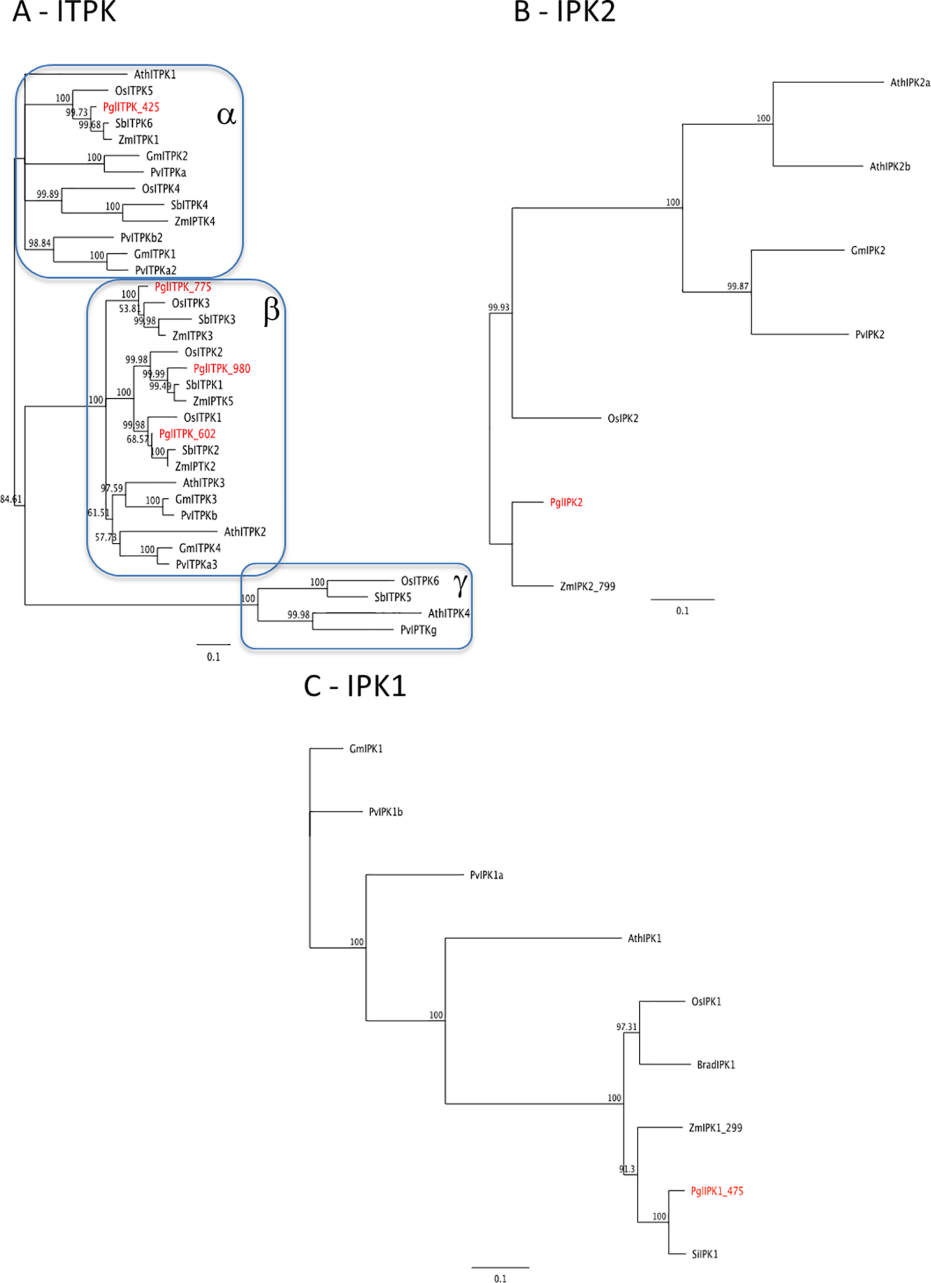
Neighbour-joining trees inferred from the deduced amino acid sequence of intermediate-late phytic acid pathway genes identified in pearl millet. **(A) ITPK, (B) IPK2, IPK1 (C)**. Bars indicate the number of amino acid substitutions per site. Sequences IDs are reported in S2 Table.

In many plants, inositol 1,3,4-triphosphate 5/6 kinase (ITPK) comprises several members that can belong to three different subgroups [48]. In pearl millet four IPTK members were identified (Pgl_GLEAN_10021425, *PglITPK_425*; Pgl_GLEAN_10017602, *PglITPK_602*; Pgl_GLEAN_10015980, *PglITPK_980*; all located on chromosomes 5 and Pgl_GLEAN_10023775, *PglITPK_775*, located on chromosomes 2). The phylogenetic tree, built using family members of different species (Arabidopsis, soybean, maize, common bean, etc), showed that the PglITPK_425 protein belongs to the subgroup α, while all the other pearl millet ITPKs were found in the subgroup β, clustering with orthologous proteins of maize, rice and sorghum (Fig 8A). No orthologs belonging to the subgroup γ were identified. Inositol 1,4,5-tris-phosphate kinase (IPK2), or more appropriately inositol polyphosphate multikinase, is a dual-specificity InsP_3_/InsP_4_ 6-/3-kinase that sequentially generates InsP_5_ from InsP_3_ and plays a major role in the lipid–independent route for phytic acid synthesis in the seed [33, 51, 52]. In pearl millet we identified only one gene (Pgl_GLEAN_10026485, *PglIPK2* located on chromosome 3) (Fig 8B).

The last step of phytic acid biosynthesis is catalysed by inositol polyphosphate 2-kinase (IPK1). The BLAST search for pearl millet orthologs identified two almost identical genes, Pgl_GLEAN_10010284 and Pgl_GLEAN_10018475 (located on chromosomes 3 and 1, respectively), which differ for 577 bp (192 aa) present at the 5’ end of Pgl_GLEAN_10018475 (S3 Fig). The multiple alignment of the deduced amino acid sequences of the two pearl millet *IPK1* genes with those of other IPK1 proteins showed that Pgl_GLEAN_10018475 protein contained extra 157/159 aa at the N-terminus, while the N-terminal of the Pgl_GLEAN_10010284 protein was 33/35 aa shorter compared to other plant IPK1 proteins (S3 Fig). BLAST search of the extra 157/159 aa at the N-terminus of Pgl_GLEAN_10018475 protein showed that this region corresponds to an ATP/GTP binding protein-like of maize (AQK44452), which function is unknown. This suggests a possible mistake in the assembly of Pgl_GLEAN_10010284 and Pgl_GLEAN_10018475, further supported by the finding that, although sharing 100% identity in the overlapping region, the two genes are located in two different chromosome positions. For this reason, we considered only the IPK1 protein coded by Pgl_GLEAN_10018475, devoid of 157 aa at the N-terminus to build the phylogenetic tree (Fig 8C).

#### Phytic acid accumulation: MRP transporter

**Fig 9:**
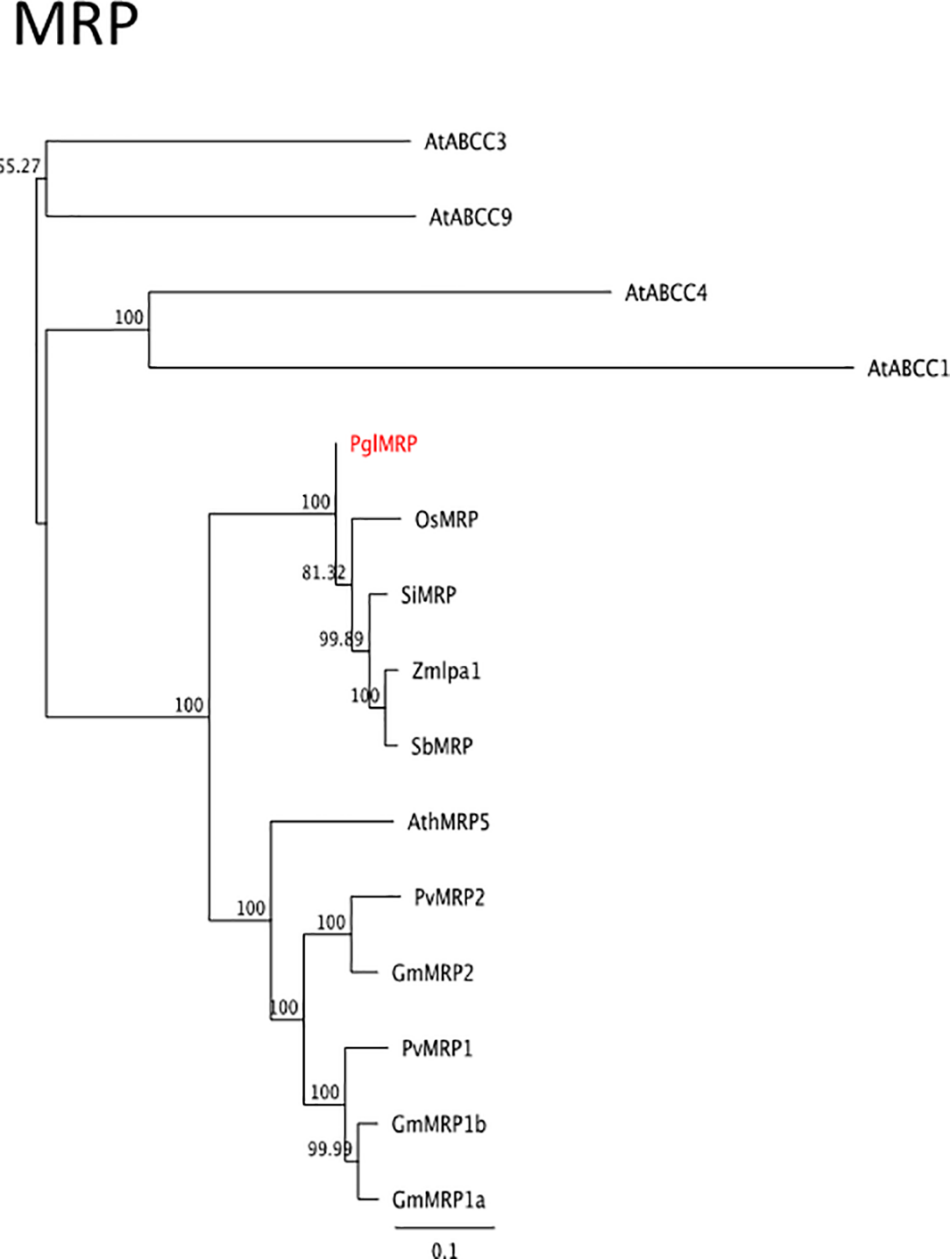
Neighbour-joining trees inferred from the deduced amino acid sequence of *MRP* phytic acid transporter gene identified in pearl millet. Bars indicate the number of amino acid substitutions per site. Sequences IDs are reported in S2 Table.

An important protein involved in the accumulation of phytic acid is a tonoplast phytic acid transporter, that is a multidrug resistance-associated protein (MRP) belonging to the ABBCC cluster of plant ABC transporters [53]. The BLAST search allowed the identification of only one putative MRP orthologue, Pgl_GLEAN_10009510 (*PglMRP*) located on chromosome 5. We found that the predicted CDS for this gene was lacking a large portion in the second half of the protein. A careful analysis of the genomic region revealed that a genomic region of 2418 bp, extending from the middle of Exon 4 to Intron 9, was absent from the CDS (S4 Fig). We therefore reannotated the gene according to the exon intron structure of this region, and the new PglMRP deduced protein was found to be 1320 aa long but still lacking about 120 aa at the N-terminus, compared to the close homolog maize ZmMRP protein (S5 Fig). This PglMRP protein was used for the phylogenetic analyses. For the construction of the phylogenetic tree we included different members of ABCC family in order to verify the correct clustering of the pearl millet ortholog (S2 Table, Fig 9). The analysis of the multiple alignment showed that PglMRP clusters with cereals MRP proteins including maize and rice phytic acid transporters, which are a subgroup of the known phytic acid transporters identified in a number of plant species.

#### C-glycosylflavones biosynthesis

**Fig 10:**
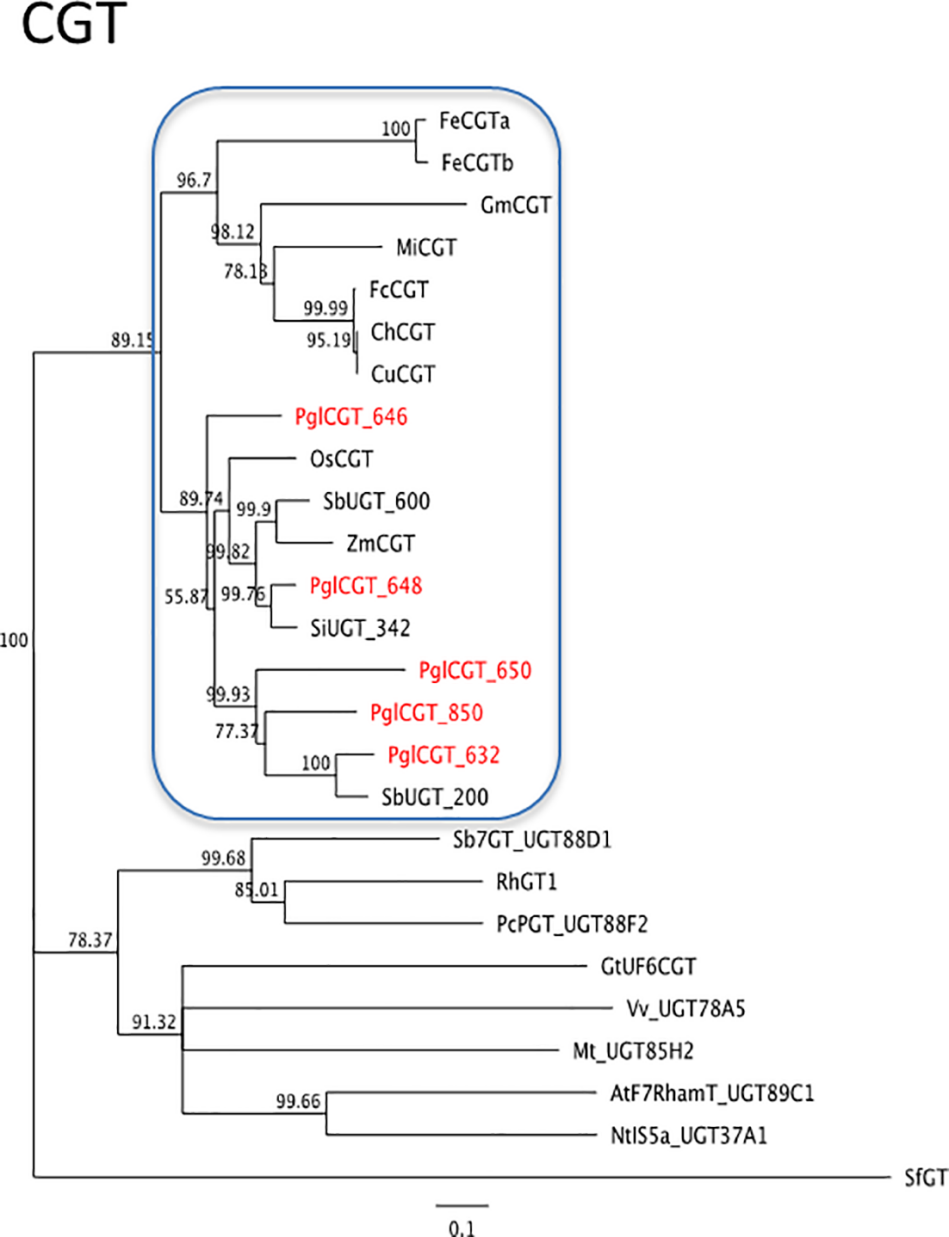
Neighbour-joining trees inferred from the deduced amino acid sequence of *CGT* gene identified in pearl millet. Bars indicate the number of amino acid substitutions per site. Sequences IDs are reported in S2 Table.

As far as C-GFs are concerned, little information on genes involved in specific steps of their biosynthetic pathway is available. A number of papers reported the characterization and molecular identification of C-glycosyl transferases (CGT) catalysing the formation of C-GFs in different species [35–38,42], while only one paper reported the isolation and characterisation of a rice flavanone 2-hydoxylase, which generates 2-hydoxyflavanone substrates for C-glucosylation by CGT [54] (S2 Fig). On the basis of these information we decided to look for a pearl millet candidate gene coding for a putative C-glycosylflavone transferase involved in the production of vitexin and orientin. For this purpose, we used maize and rice CGTs amino acid sequences to BLAST pearl millet proteins dataset (S2 Table). Five pearl millet CGT candidates were identified and the highest similarity to the query sequences were the proteins coded by the Pgl_GLEAN_10014648 and Pgl_GLEAN_10014646 genes (both located on chromosome 2 at a distance of about 39,000 bp) that share 75.5% and 60.7% similarity with ZmCGT and 70.4% and 68% similarity with OsCGT proteins, respectively. However, both these genes code for proteins that are about 150 aa shorter than the other CGTs. A careful look at the genomic regions of Pgl_GLEAN_10014648 and Pgl_GLEAN_10014646 genes revealed that region upstream of their CDS contained about 100 more nucleotides and then there was an unresolved region containing unidentified nucleotides. We included the nucleotides upstream of the CDS to extend the deduced amino acid sequences at the N-terminus. The resulting proteins PglCGT_648 and PglCGT_646 were 30 and 33 aa longer, respectively (S6 Fig). We then used all the pearl millet CGT candidates to build a phylogenetic tree together with the deduced amino acid sequences of known plant CGTs and other plant UGTs belonging to different orthologous groups [38] (Fig 10, S2 Table). From this analysis PglCGT_646 appears to be the ancestor of two subgroups, one clustering PglCGT_648 together with maize and rice CGTs, and the other one clustering all the remaining pearl millet CGTs. On this basis, we presumed that PglCGT_648 was the best CGT candidate.

### Gene expression during pearl millet seed development and in plant organs

To verify the role of the genes involved in phytic acid and C-GFs synthesis we performed gene expression analyses by qRT-PCR in developing grains of the progeny of the two inbred lines selected for their contrasting content of phytic acid (Fig 2, Fig 5). These two lines also showed a consistent difference in total C-GFs accumulation, with the line ICML157032_lpa_hcgf having a 2.3 fold higher content of C-GFs than the line ICML157001_hpa_lcgf (Fig 3, S5 Table). When the analysis was repeated on mature seeds of the second generation, this difference was still detectable with the same fold change. It should be pointed out that compared to the seeds of the corresponding parental plant, seeds of the progenies accumulated less total C-GFs and in the case of the ICML157001_hpa_lcgf line the C-GFs composition was slightly different (Fig 5), most probably due to the different growth conditions of the plants (open field in Niger for the first analysed generation and phytotron for the second one), as it is well known that synthesis of secondary metabolites is often induced by environmental stimuli such as light, abiotic stresses, etc. [55]. As to other storage compounds, phytic acid accumulation starts at the end of the differentiation phase (in which cell divisions occur), and then it significantly increases during the middle stage of seed development (during which reserves begin to be accumulated). We hypothesized that the different accumulation levels of phytic acid observed in the seeds of the two lines ICML157032_lpa and ICML157001_hpa would correlate with differences in the expression of key genes.

**Fig 11:**
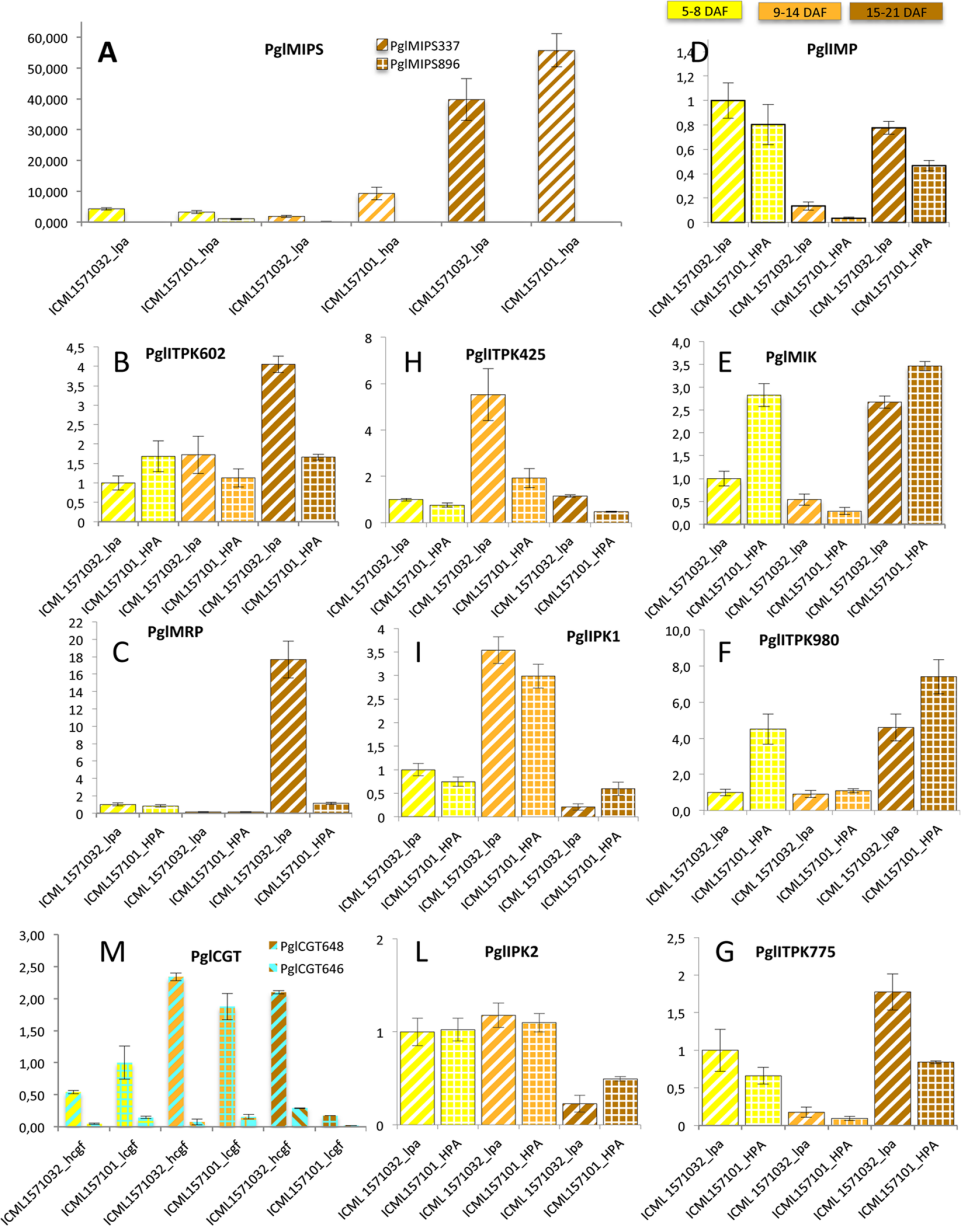
**Expression analysis by quantitative RT-PCR of genes involved in phytic acid (A-L) and C-GFs (M) pathway in developing seeds of the two inbred lines contrasting for their content of phytic acid and C-GFs (ICML157032_lpa_hcgf and ICML157101_hpa_lcgf).** Colour of the bars indicate different seed developmental stages: yellow, 5–8 DAF; orange, 9–14 DAF; brown, 15–21 DAF. Striped bars indicate ICML157032_lpa_hcgf line, dashed bars indicate ICML157101_hpa_lcgf line. (A) expression of *PglMIPS_337* and *PglMIPS_896*; (B) expression of *PglITPK_602*; (C) expression of *PglMRP;* (D) expression of *PglIMP*; (E) expression of *PglMIK*; (F) expression of *PglITPK_980*; (G) expression of *PglITPK_775*; (H) expression of *PglITPK_425*; (I) expression of *PglIPK1*; (L) expression of *PglIPK2*; (M) expression of *PglCGT_646* and *PglCGT_648*. Samples at early stage of seed development were used as calibrators.

MIPS is the first enzyme involved in phytic acid synthesis and is encoded by a key gene of the pathway. The two pearl millet *MIPS* genes showed different expression patterns during seed development. *PglMIPS_896* expression level was almost undetectable, on the contrary *PglMIPS_337* was well expressed and reached the highest levels at late developmental stage (15–21 DAF) (Fig 11A). Interestingly, *PglMIPS_337* expression was higher in developing seeds of the ICML157001_hpa line compared to the ICML157032_lpa one, that accumulated less phytic acid, suggesting that its expression may play a role in the final phytic acid content in the grains. We identified four genes coding for putative ITPKs. Among them, PglITPK_425 and PglITPK_980 showed the highest expression levels during seed development (S7 Fig). However they were expressed with a different timing: the *PglITPK_980* gene was more expressed at early and late stages (ICML157001_hpa line), while *PglITPK_425* transcript was accumulated at the highest levels at the middle stage.

In general, depending on the line, different trends of expression of some genes could be detected. *PglITPK_602* and *PglMRP* genes showed an increase in expression in the ICML157032_lpa line only at late developmental stage (15–21 DAF), while they were not so modulated in the ICML157001_hpa line (Fig 11B and 11C). A number of genes, including *PglIMP*, *PglMIK*, *PglITPK_980* and *PglITPK_775*, had their lowest expression at the middle developmental stage (9–14 DAF) (Fig 11D–11G). However, in the case of *PglMIK* and *PglITPK_980* genes their expression at the other two developmental stages was higher in the ICML157001_hpa line, especially at the early developmental stage (5–8 DAF). Finally, *PglITPK_425* and *PglIPK1* had a higher expression at the middle stage of seed development (9–14 DAF), although in the case of *PglITPK_425* only for the ICML157032_lpa line (Fig 11H and 11I). *PglIPK2* gene expression was not much different between the two lines and it slowed down at late developmental stage (14–21 DAF) (Fig 11L).

The expression of *PglCGT_648* showed the highest levels at the middle and late stage of seed development (9–14 DAF and 15–21 DAF). At 15–21 DAF *PglCGT_648* expression was much higher (about 10 times) in the ICML157001_lpa_hcgf line, which accumulated more total C-GFs than the line ICML157032_hpa_lcgf. Conversely, *PglCGT_646* expression was very low at all stages of seed development (Fig 5).

**Fig 12:**
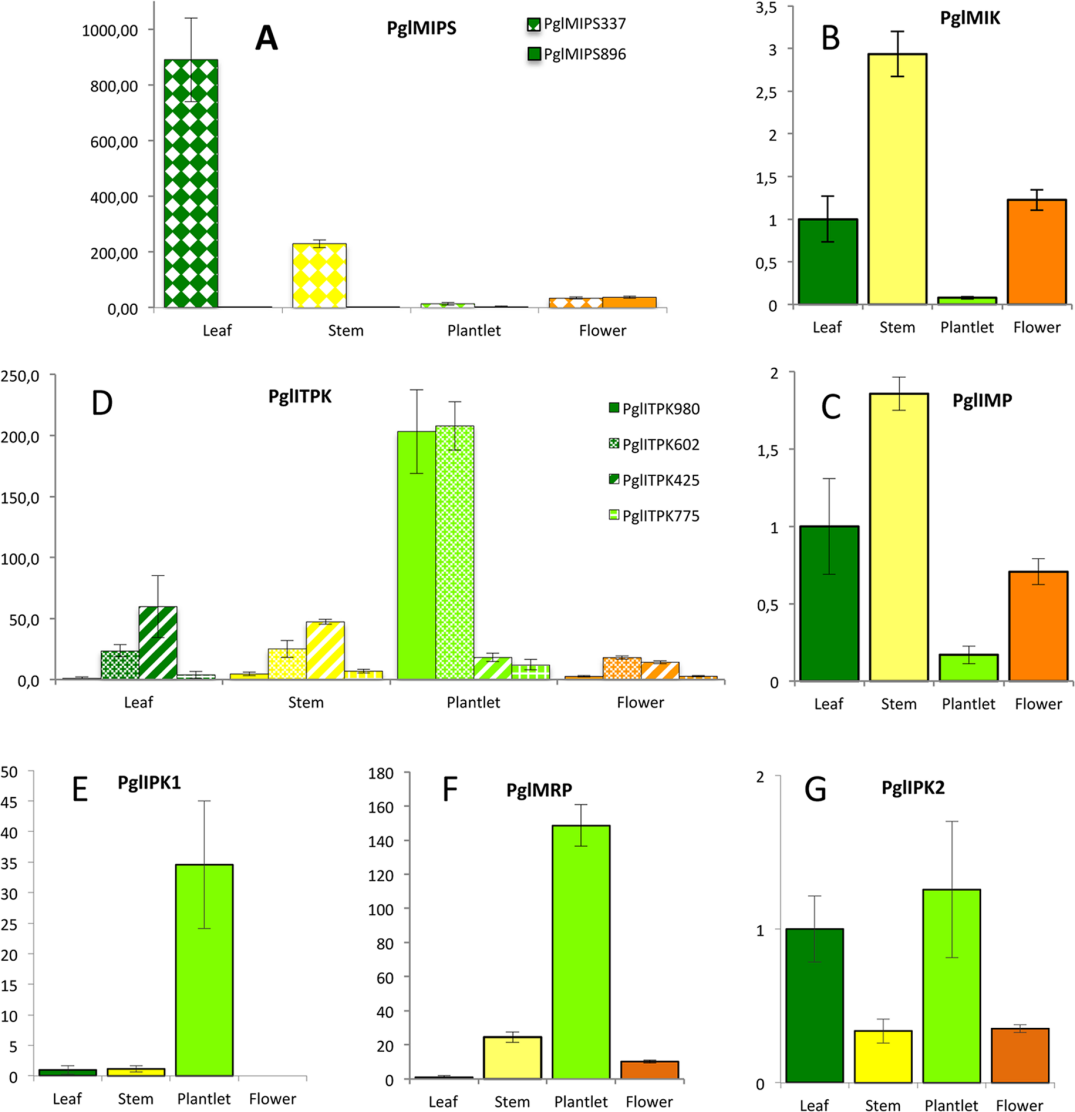
Expression analysis by quantitative RT-PCR of genes involved in phytic acid pathway in leaves, stems, plantlets and anthesis flowers of the reference line SDEB4L-160P6. Color of the bars indicate different organs: dark green, leaf; yellow, stem; light green, plantlet; orange, flower. (A) expression of *PglMIPS_337* and *PglMIPS_896*; (B) expression of *PglMIK*; (C) expression of *PglIMP;* (D) expression of *PglITPK_980*, *PglITPK_602*, *PglITPK_425* and PglITPK_775; (E) expression of *PglIPK1*; (F) expression of *PglMRP*; (G) expression of *PglIPK2*. Leaf samples were used as calibrators.

Gene expression was also investigated in other plant organs, i.e. leaves, stems, plantlets and anthesis flowers. Unexpectedly, *PglMIPS_896* transcript was not detected in leaves and very poorly accumulated in other organs (the highest expression was in flowers) compared to *PglMIPS_337* which was mostly expressed in leaves and stems, while *PglMIP_896* was not detected in leaves. (Fig 12A). *PglMIK* and *PglIMP* showed very similar expression patterns: they were mostly expressed in stems and very poorly in plantlets (Fig 12B and 12C). *PglITPK_980* and *PglITPK_602* showed the highest expression in plantlets, similarly to *PglIPK1* and *PglMRP*. *PglITPK_425* was more expressed in leaves and stems, while *PglITPK_775* was poorly expressed in all organs (Fig 12D–12F). *PglIPK2* expression was not very much modulated (Fig 12G).

## Discussion

Pearl millet is a staple food for more than 90 million people in Africa and Asia, thus the nutritional quality of the grains has a big impact on the diet of these populations. Efforts to improve this crop are increasing (reviewed by [56–58]) and very recently the sequence of pearl millet genome was released [43], opening new opportunities for accelerating breeding of this species. With the purpose to contribute to the knowledge needed for breeding programs regarding nutritional traits of pearl millet, here we analysed the content of the major antinutrient phytic acid and of the goitrogenic C-glycosylflavones of the grain and provide information on genes involved in their biosynthesis. To our knowledge this is the first report in which the variability of these compounds was assessed in a large number of samples belonging to a panel of inbred lines covering a large genetic diversity.

Our results show that a large variability exists for both phytic acid and C-GFs contents (Table 1), indicating a good potential to breed for these traits. Seed phytic acid values detected in the studied population were in line with previously reported data in pearl millet [22,59,60]. It is known that variation of P supply could significantly affect phytic acid accumulation during seed development and maturation, however it is expected that the proportion of PAP versus the total phosphate should not vary too much and remains in the range of 70–80% [61,62]. Conversely, if the observed low PAP values correlate positively with increase in inorganic phosphate and/or other lower inositol phosphates, while maintaining invariable total phosphorous content, most likely the reduction has a genetic basis [63]. As expected, we found a linear correlation of 0.894 between PAP and P_tot_, however a moderate correlation (0.610) was detected between PAP and the ratio PAP/P_tot._ This indicates that the PAP content only partially depends on P supply, as in some lines the low PAP values are accompanied by increases in free P_i_, thus indicating the existence of a genetic variability in the ability to synthesize and/or accumulate PAP. These data help us to infer that the impact of the environmental effect seems to have a secondary role on the variability of the PAP content observed on these lines. Indeed, the two lines ICML157032_lpa and ICML157101_hpa, identified for their contrasting content of PAP, also showed different PAP/P_tot_ ratios (0.516±0.058 g/100 g and 0.803±0.047 g/100 g, respectively), indicating that in line ICML157032_lpa the low PAP content was accompanied by an increase in free P_i_. Moreover, the different ability of the two lines to accumulate phytic acid was confirmed in the grains of the progeny (Fig 2). These data correlate and are supported by the finding that the expression of *PglMIPS_337* in developing seeds is reduced in the ICML157032_lpa line. MIPS is the first enzyme activity involved in phytic acid synthesis and is encoded by a key gene for phytic acid accumulation. In fact, its expression is often reduced in *lpa* mutants, even if not affected in the *MIPS* gene [62–64]. Also the *PglMIK* and *PglITPK_980* genes were less expressed in the ICML157032_lpa line, indicating that the genetic basis of the trait involves an attenuation of the biosynthetic pathway (Fig 11A, 11E and 11F). Other genes involved in phytic acid pathway showed an opposite behaviour with higher expression observed in the ICML157032_lpa line compared to the ICML157101_hpa one. This is the case of *PglMRP*, *PglITPK_602*, *PglITPK_425*, *PglITPK_775* and *PglIMP* (Fig 11B, 11C, 11G, 11H and 11D). Although this finding might seem in contrast to what expected and reported above, it is not surprising. In fact, in Arabidopsis it was reported that different *lpa* mutants showed changes in the expression of other genes of the pathway [65], and a similar behaviour was shown for the common bean *lpa1* mutant [66]. Furthermore, alterations in the content of phytic acid pathway related compounds, such as *myo*-inositol, inositol phosphates, galactinol, or galactose-1-P, have been reported in different *lpa* mutants (reviewed by [53]). Overall, our results together with those already reported in other species, strongly support the hypothesis that the expression of the genes of the pathway is modulated by other constituents of phytic acid metabolism. Interestingly, we observed that the most contrasting gene expressions between the ICML157032_lpa and the ICML157101_hpa lines were observed for the genes involved in the lipid independent pathway (Fig 11A–11G), the branch that is assumed to work specifically in the seeds and other organs that accumulate phytic acid as a storage compound [33].

Varying values of polyphenol content in pearl millet grain were reported in literature: [67] reported values ranging between 590 and 1060 mg/100 g; [68] reported 304 and 444 mg/100 g for 2 cultivars of pearl millet and [69] reported 318 and 293 mg/100 g as the polyphenol content of other two cultivars. As regards in particular C-GFs, according to [70], the C-glycosylflavones content of pearl millet, expressed as glucosyl vitexin equivalents, ranges from 87 to 259 mg/100 g. The data we collected regarding the flour amount of vitexin, orientin and glucosyl vitexin range from from 15.29 to 541.10 μg/g, but taking into account also glucosyl orientin and the two molecules that we very recently showed to be present in pearl millet, i.e. rhamnosyl vitexin and rhamnosyl orientin, these values should become much higher, approximately doubled. In fact, as judged from the values of the areas of the respective chromatographic peaks, the amounts of the “novel” C-GFs together with glucosyl orientin are about 40% of the total amounts of all the C-GFs molecules detectable in our analyses (Fig 6). To our knowledge this is the first study reporting a detailed qualitative and quantitative analysis of C-GFs in pearl millet grains and providing an analysis of the variability of the trait in a broad population covering a large genetic diversity.

As shown in Fig 6, our results clearly demonstrate that a high variability of the grain C-GFs level exists across the 97 investigated pearl millet inbred lines and regards both the molecules already known to be present in pearl millet, glucosyl vitexin, vitexin, glucosyl orientin and orientin, as well as the two “novel” ones, i.e. rhamnosyl vitexin and rhamnosyl orientin. Our data are in line with those reported by [71] who found that glucosyl vitexin and glucosyl orientin were more abundant than vitexin and were present in pearl millet in ratios of 29:11:4, respectively. The goitrogenic and antithyroid effects of millet diets, extracts of millet, and certain pure compounds contained therein were determined *in vivo* in rats and *in vitro* using porcine thyroid slices and a thyroid peroxidase (TPO) assay more than twenty years ago [23,24]. The results of those studies showed that the three C-GFs present in millet, i.e. glucosyl vitexin, glucosyl orientin, and vitexin, inhibited TPO activity. However, no direct information about the goitrogenicity of orientin, rhamnosyl orientin and rhamnosyl vitexin is so far available. Given the strict similarity of the six chemical structures, it is quite probable that the three latter molecules are goitrogenic too and thus the histogram shown in Fig 3 may well represents the genetic variability, besides of the C-GFs level, also of the goitrogenicity degree of the investigated lines.

In order to reduce the concentrations of C-GFs in such a way to improve the health benefits derived from pearl millet food, some understanding of the biosynthetic route and the interactions between the compounds under analysis is in fact of value. Therefore, we verified the correlations between the relative amounts of the six C-GFs accumulated in the grains of the investigated pearl millet lines by calculating the correlation coefficients for each couple of compounds. According to literature, all the C-GFs are formed from one biosynthetic pathway starting from phenylalanine, flowing through cinnamic acid, coumaric acid, coumaroyl CoA, naringenin chalcone and the flavanone naringenin [72], intermediate compound that can be transformed in vitexin or in eriodictyol (which in turn is transformed in orientin), thus branching the biosynthetic route.

**Fig 13:**
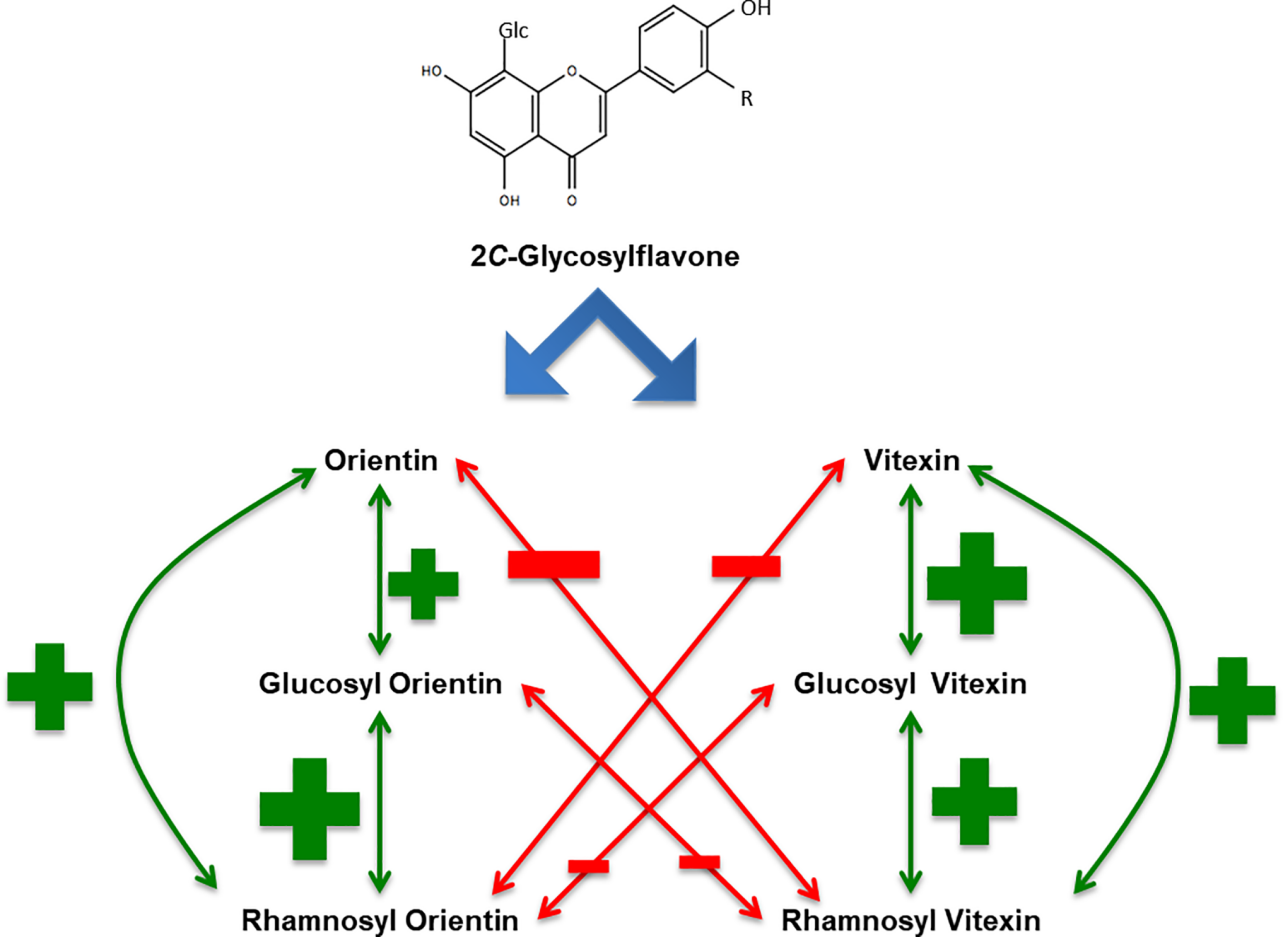
Scheme representing the two branches of C-GFs biosynthesis in pearl millet. The aglycone (either apigenin or luteolin) is converted to orientin or vitexin. These C-GFs than can be further glycosylated resulting in the production of corresponding glucosylated o rhamnosylated forms. Arrows indicate significant correlations (p<0.01), green lines and plus symbol correspond to positive correlation, red lines and minus symbol indicate a negative correlation. The size of plus and minus symbols indicate the strength of the correlation, as reported in Table 3.

Highly significant negative linear correlations were found when plotting the seed levels of couples of CGFs belonging to the two different biosynthetic branches using naringenin as the common precursor and orientin or vitexin as alternative intermediate product, respectively. Conversely, significant positive linear correlations were found for couples of C-GFs belonging to the same biosynthetic route, in particular for glucosyl orientin vs. rhamnosyl orientin and vitexin vs glucosil vitexin (Fig 13). These results confirm and reinforce the validity of the data obtained from the analyses carried out, since they are consistent with the classical mechanism controlling metabolite fluxes in branched pathways: whenever a substrate is used more efficiently by one path, the other concurrent path is obviously and proportionally less fed, thus producing less final products.

Besides understanding of the biosynthetic interactions between the compounds under analysis, it is also important to identify key genes involved in the biosynthetic pathway. The key enzyme is C-glycosyl transferase that was shown to be sufficient together with a flavanone2-hydroxylase for the synthesis of C-GFs in engineered tobacco and yeast expression host systems [72]. CGTs were identified and characterised in a number of plant species, such as rice, maize, buckwheat, soybean, gentian and citrus [35–38,42]. All these CGTs catalyse flavone C-glycosylation at either C-6 or C-8 and specific amino acid residues (His20, Asp85, and Arg292, referred to soybean CGT) within the N-terminal acceptor binding pocket were shown to be necessary for the CGT activity [73]. These amino acid residues were conserved in all the pearl millet putative CGTs we identified with the exception of PglCGT_646 and PglCGT_648, which contained only the conserved Arg292, since they are lacking about 140 aa at the N-terminus (S6 Fig, light blue dots). All pearl millet CGTs here identified clustered in the same clade with other plant CGTs, however PglCGT_648 was the most similar to the maize CGT and rice ones and was the most related to the finger millet ortholog (SiUGT_342), therefore we assumed that most closely related gene was most likely the one with highest probability to have in pearl millet the same function of maize and rice CGTs. An *in silico* analysis of *OsCGT* and *ZmCGT* expression using the e-FP Browser [74] showed that rice *CGT* gene is highly expressed in developing seed, especially at very early developmental stage, while maize *CGT* is less expressed in seeds compared to leaves at different stages of plant development (S8 Fig). We showed that *PglCGT_648* was significantly expressed in developing seeds at middle and late development stages (9–14 DAF and 15–21 DAF) and found a positive correlation between the *PglCGT_648* expression and CGFs accumulation in the progenies of the originally identified lines derived from ICML157032_lpa_hcgf and ICML157101_hpa_lcgf lines. ICML157032_lpa_hcgf accumulates about 2.3 times more CGFs and *PglCGT_648* expression is about 2.3 times higher compared to ICML157101_hpa_lcgf line (Fig 6 and Fig 11M). This finding, together with the finding that *PglCGT_646* is poorly expressed, strongly support the hypothesis that *PglCGT_648* is involved in CGFs synthesis and accumulation in the grains. Of course we cannot rule out a contribution of the other *PglCGTs*, and it will be of interest to perform a more exhaustive study of the transcriptional regulation of each *PglCGTs* in developing pearl millet grains.

## Conclusions

The research described in the present paper was the first phase of a program designed to improve pearl millet—derived food by accomplishing the following objectives: 1) reduction of the level of phytic acid accumulated in the grain; 2) reduction of the potential health problems derived from the presence in the grain of the goitrogenic C-glycosylflavones.

Regarding the first objective we demonstrated that substantial variability exists as regards the seed level of phytate and we identified contrasting lines in which lpa and hpa phenotype were genetically inherited.

For what concerns the second objective, the results we presented here clearly show that a high variability exists across the panel of investigated pearl millet inbred lines as to the seed levels of the six C-GFs that were analysed and quantified by HPLC. Since the greater is the variability for an individual trait, the greater the opportunity for genetic improvement by plant breeders, the next phase of the research will focus on creating new germplasm with increased nutritional quality. Potential parents for use in breeding programs can be chosen from the surveyed material among the inbred lines with seeds containing the least amount of C-GFs.

In addition, last but not least, we also produced molecular data regarding phytic acid pathway and showed which genes are more relevant for phytic acid biosynthesis in the seeds. These results together with a preliminary analysis of pearl millet orthologous genes for C-glycosylflavones biosynthesis open the way to dissect the genetic determinants controlling key seed nutritional phenotypes and to the characterization of their impact on grain nutritional value in pearl millet.

## Supporting information

S1 Table List of pearl millet inbred lines. List of pearl millet inbred lines used in this work, reporting line number, entry number and pedigree.
(XLSX)

S2 Table List of genes used for BLAST searches and phylogenetic analyses. List of genes used for BLAST searches (red colour) and phylogenetic analyses. Corresponding locus and/or gene/protein ID are given for each gene/protein in the list. For pearl millet genes the corresponding gene name and pseudochromosomal location are also given.
(XLSX)

S3 Table List of primers used for qRT-PCR analyses. (XLSX)

S4 Table Phytate content in pearl millet inbred lines. Phytic acid phosphorus (PAP), total phosphorus (P_tot_), ratio of phytic acid content to total phosphorus (PAP/P_tot,_) and P_i_ values and relative standard deviations for each of the 145 pearl millet lines from a panel of inbred lines covering a large genetic diversity.
(XLSX)

S5 Table C-GFs content in pearl millet inbred lines. Orientin, glucosyl orientin, rhamnosyl orientin, vitexin, glucosyl vitexin and rhamnosyl vitexin content, expressed as peak area, and orientin, vitexin and glucosyl vitexin content, expressed as μg/g of seed flour, for each of the 97 pearl millet lines from a panel of inbred lines covering a large genetic diversity.
(XLSX)

S1 Fig Schematic representation of phytic acid biosynthetic pathway. The substrate supply, lipid independent (red) and lipid dependent (dark grey) sub-pathways for InsP_6_ synthesis are indicated. MIPS, *myo*-inositol-3-phosphate synthase; IMP, *myo*-inositol-phosphate monophosphatase; MIK, *myo*-inositol kinase; IPK2, inositol 1,4,5-tris-phosphate kinase; ITPK, inositol 1,3,4-triphosphate 5 ⁄ 6-kinase; IPK1, inositol 1,3,4,5,6 penta*kis*phosphate 2-kinase; PtdIS, phosphatidyl inositol phosphate synthase; PtdI4K, phosphatidyl inositol 4-kinase; PtdI5K, phosphatidyl inositol 5-kinase; PtdIns, phosphatidyl inositol; PtdIns(4)P_1_, phosphatidyl inositol 4-phosphate; PtdIns(4,5)P_2_, phosphatidyl inositol 4,5-biphosphate; PLC, phospholipase C.
(TIF)

S2 Fig Scheme of C-glycosylflavones biosynthesis. C-glycosylflavones biosynthesis from flavanone via C-glycosylation of 2-hydroxyflavanone or flavone. Enzymatic steps controlled by flavanone 2-hydoxylase (CYP93G2) and C-glycosyl transferase (CGT) are shown.
(TIF)

S3 Fig Multiple alignment of IPK1 proteins. Multiple alignment of the deduced amino acid sequences of genes coding for IPK1 from pearl millet (*PglIPK1_284* and *PglIPK1_475*) and other plant species (names are as in S2 Table).
(PDF)

S4 Fig Exon-Intron structure of *PglMRP* gene. Schematic representation of the alignment betweeen *PglMRP_CDS* and its corresponding genomic region (*PglMRP*_genomic) showing the Exon-Intron structure before and after the reannotaion (exon green and introns grey blocks numbered from E1 to E11 and from I1 to I10, respectively).
(TIF)

S5 Fig Multiple alignment of MRP proteins. Multiple alignment of the deduced amino acid sequences of genes coding for MRP transporter from pearl millet (*PglMRP*) and other plant species (names are as in S2 Table).
(PDF)

S6 Fig Multiple alignment of CGT proteins. Multiple alignment of the deduced amino acid sequences of genes coding for CGT from pearl millet (*PglCGT_646* and *PglCGT_648*) and other plant species (names are as in S2 Table). Red arrow indicates the start codon according to the CDS of *PglCGT_646* and *PglCGT_648* as reported in the annotated pearl millet genome. Blue dots indicate conserved amino acid residues within the N-terminal acceptor binding pocket.
(PDF)

S7 Fig Comparative expression analysis of *PglITPK* genes. Comparative expression analysis by quantitative RT-PCR of *PglITPK* genes, *PglITPK_980*, *PglITPK_602*, *PglITPK_475*, *PglITPK_775* in developing seeds of the two inbred lines contrasting for their content of phytic acid (ICML157032_lpa and ICML157101_hpa). Early stage of seed development sample was used as calibrator.
(TIF)

S8 Fig Expression analysis of rice and maize *CGT* gene. Expression analysis of **(A)** LOC_Os06g18010 (*OsCGC*) and **(B)** GRMZM2G083841 (*ZmCGT*) according to rice and maize eFP browser at bar.utoronto.ca.
(TIF)
