# Supplementary figures and images for: Antinutritional factors in pearl millet grains: Phytate and goitrogens content variability and molecular characterization of genes involved in their pathways
Source: PLoS One. 2018 Jun 1;13(6):e0198394. doi: 10.1371/journal.pone.0198394 (PMC5983567; doi:10.1371/journal.pone.0198394)

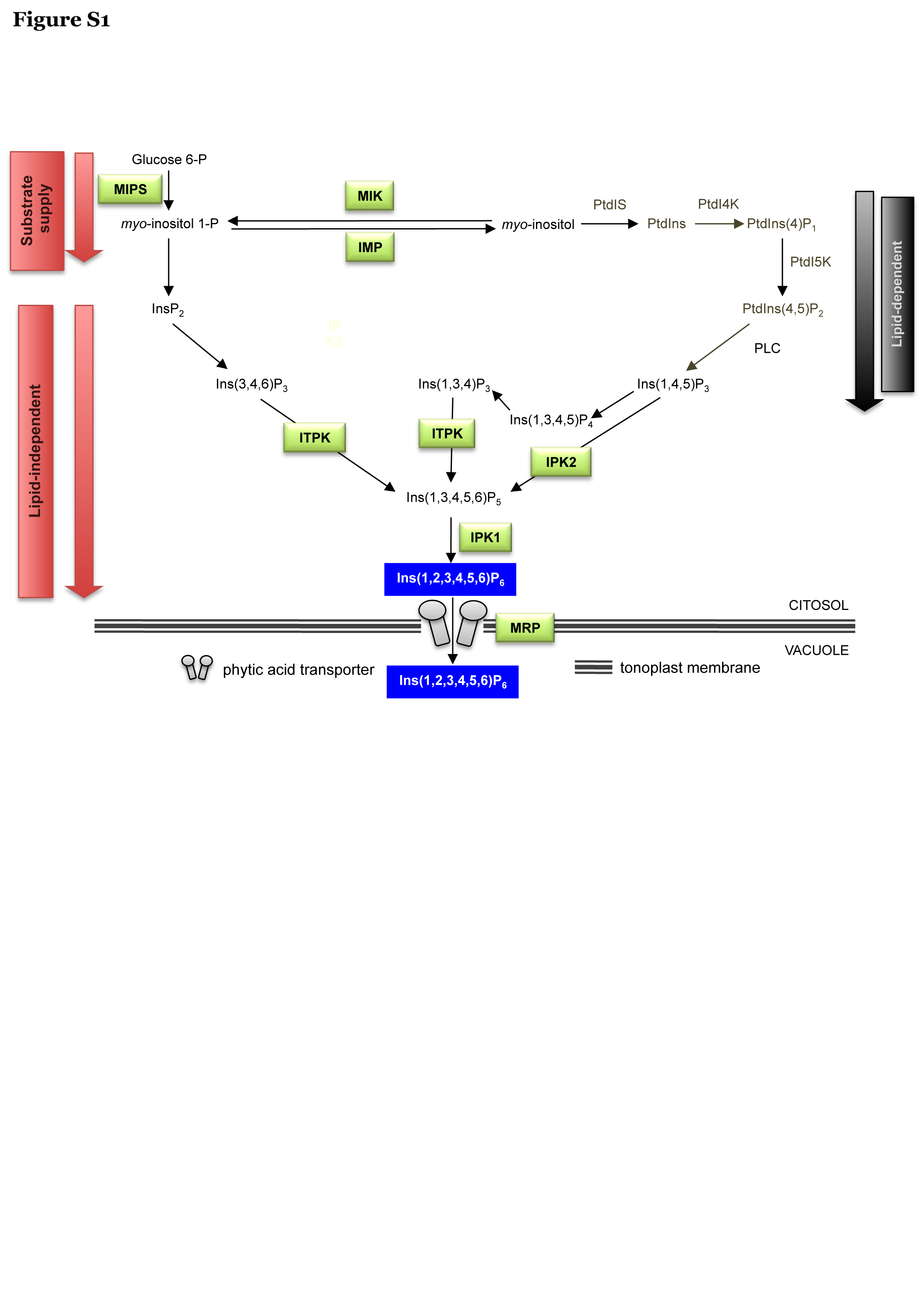

Supplement: S1 Fig — The substrate supply, lipid independent (red) and lipid dependent (dark grey) sub-pathways for InsP6 synthesis are indicated. MIPS, myo-inositol-3-phosphate synthase; IMP, myo-inositol-phosphate monophosphatase; MIK, myo-inositol kinase; IPK2, inositol 1,4,5-tris-phosphate kinase; ITPK, inositol 1,3,4-triphosphate 5 ⁄ 6-kinase; IPK1, inositol 1,3,4,5,6 pentakisphosphate 2-kinase; PtdIS, phosphatidyl inositol phosphate synthase; PtdI4K, phosphatidyl inositol 4-kinase; PtdI5K, phosphatidyl inositol 5-kinase; PtdIns, phosphatidyl inositol; PtdIns(4)P1, phosphatidyl inositol 4-phosphate; PtdIns(4,5)P2, phosphatidyl inositol 4,5-biphosphate; PLC, phospholipase C. (TIF) [file pone.0198394.s006.tif]

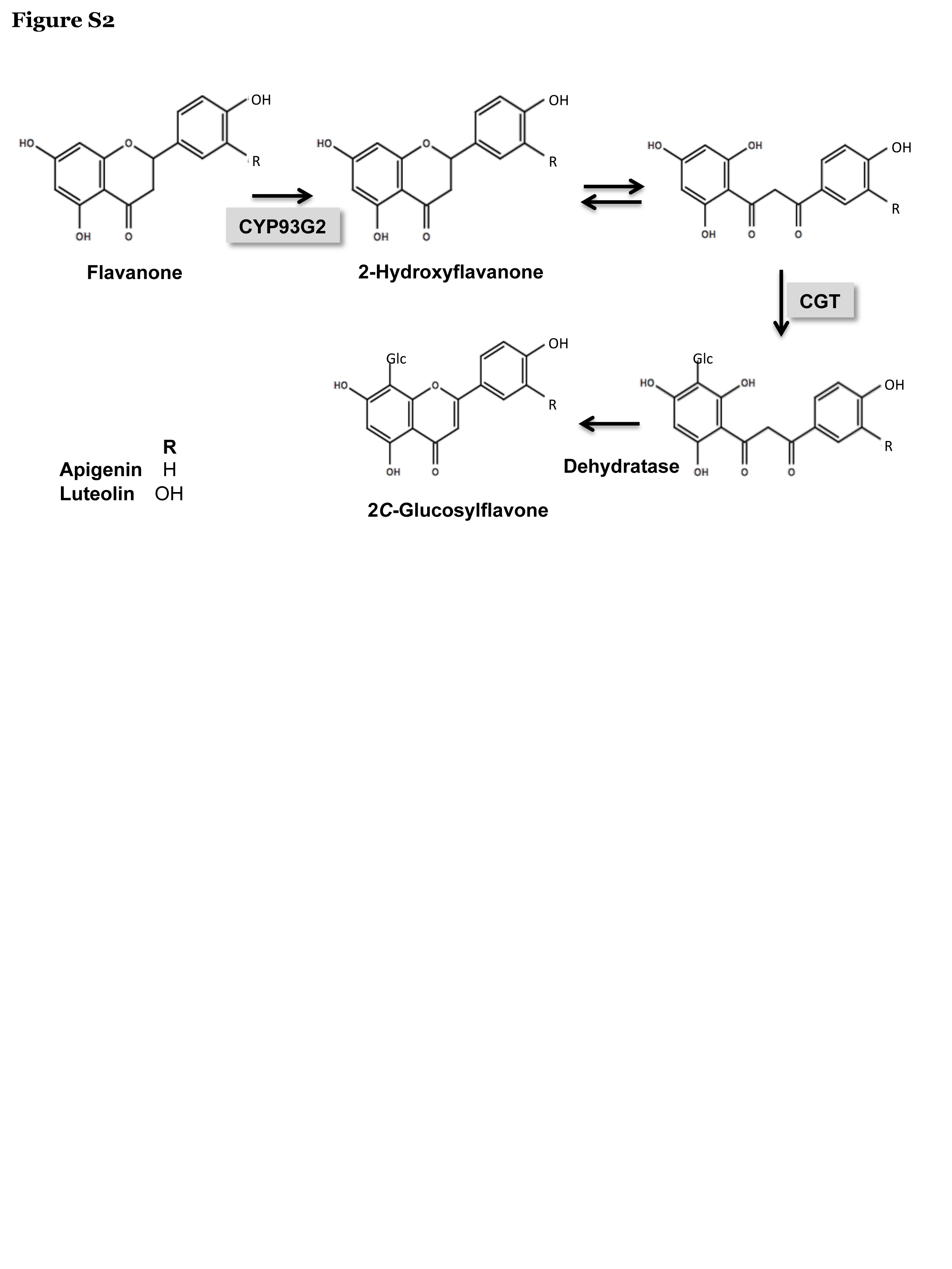

Supplement: S2 Fig — C-glycosylflavones biosynthesis from flavanone via C-glycosylation of 2-hydroxyflavanone or flavone. Enzymatic steps controlled by flavanone 2-hydoxylase (CYP93G2) and C-glycosyl transferase (CGT) are shown. (TIF) [file pone.0198394.s007.tif]

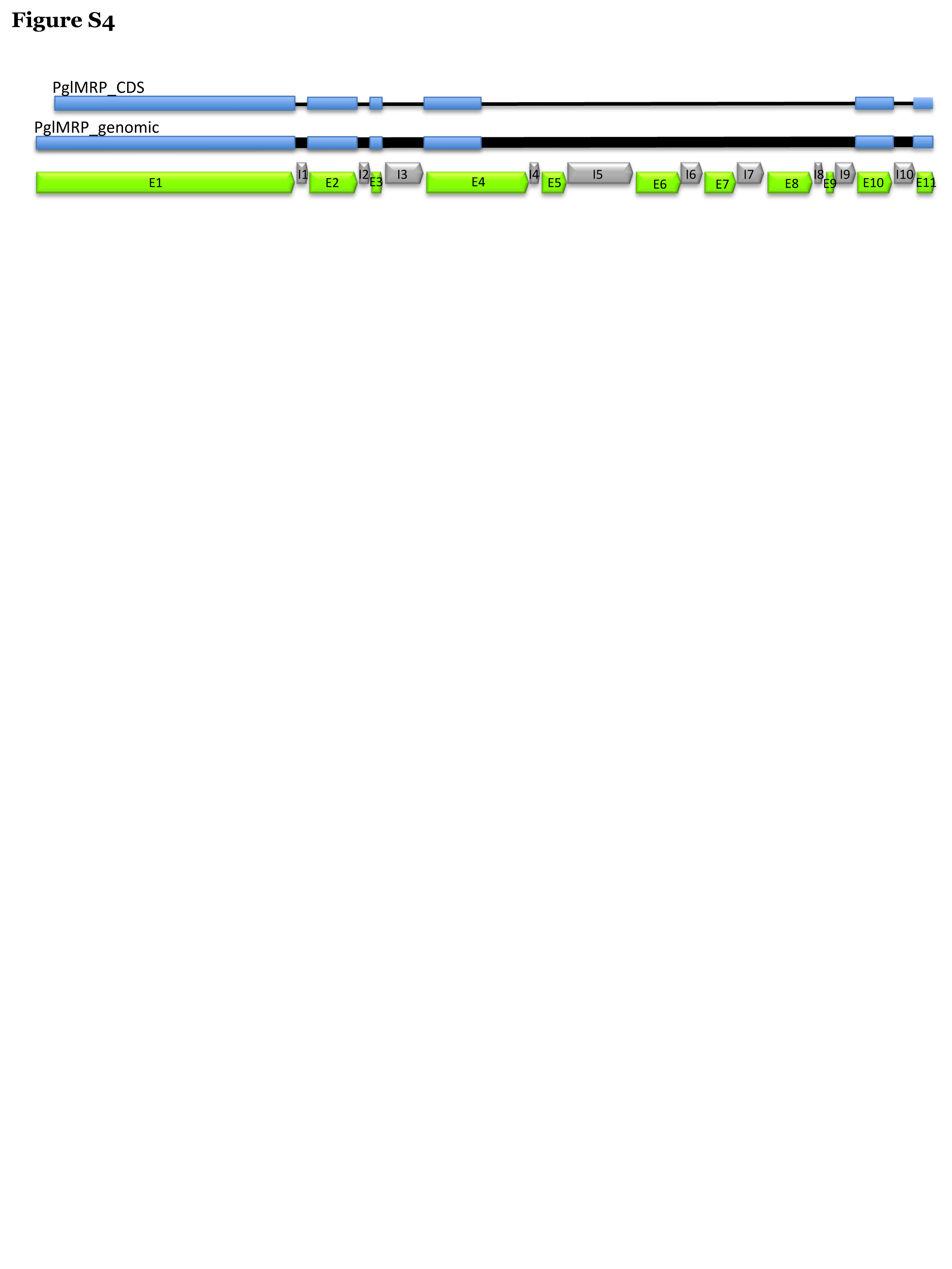

Supplement: S4 Fig — Schematic representation of the alignment betweeen PglMRP_CDS and its corresponding genomic region (PglMRP_genomic) showing the Exon-Intron structure before and after the reannotaion (exon green and introns grey blocks numbered from E1 to E11 and from I1 to I10, respectively). (TIF) [file pone.0198394.s009.tif]

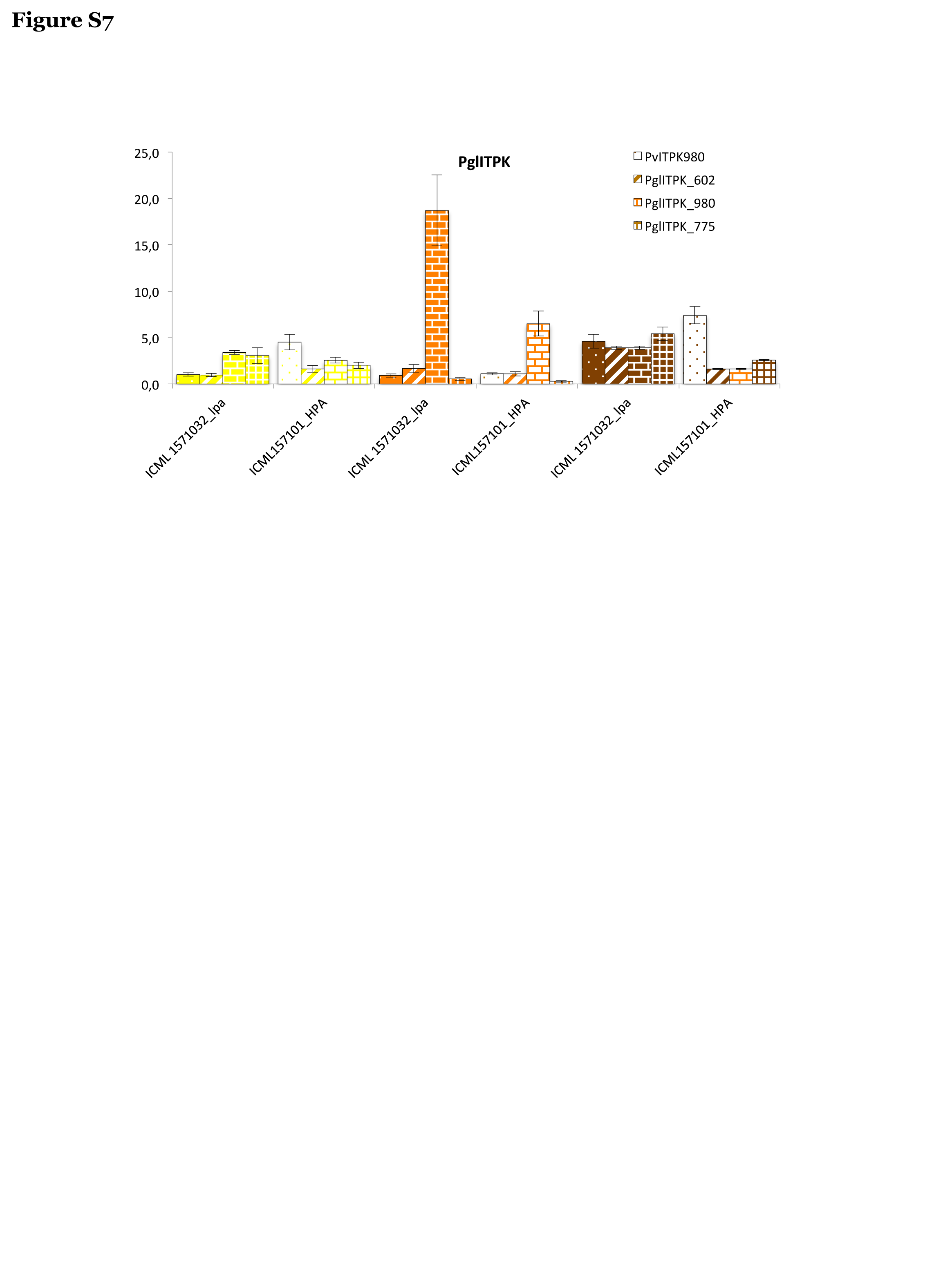

Supplement: S7 Fig — Comparative expression analysis by quantitative RT-PCR of PglITPK genes, PglITPK_980, PglITPK_602, PglITPK_475, PglITPK_775 in developing seeds of the two inbred lines contrasting for their content of phytic acid (ICML157032_lpa and ICML157101_hpa). Early stage of seed development sample was used as calibrator. (TIF) [file pone.0198394.s012.tif]

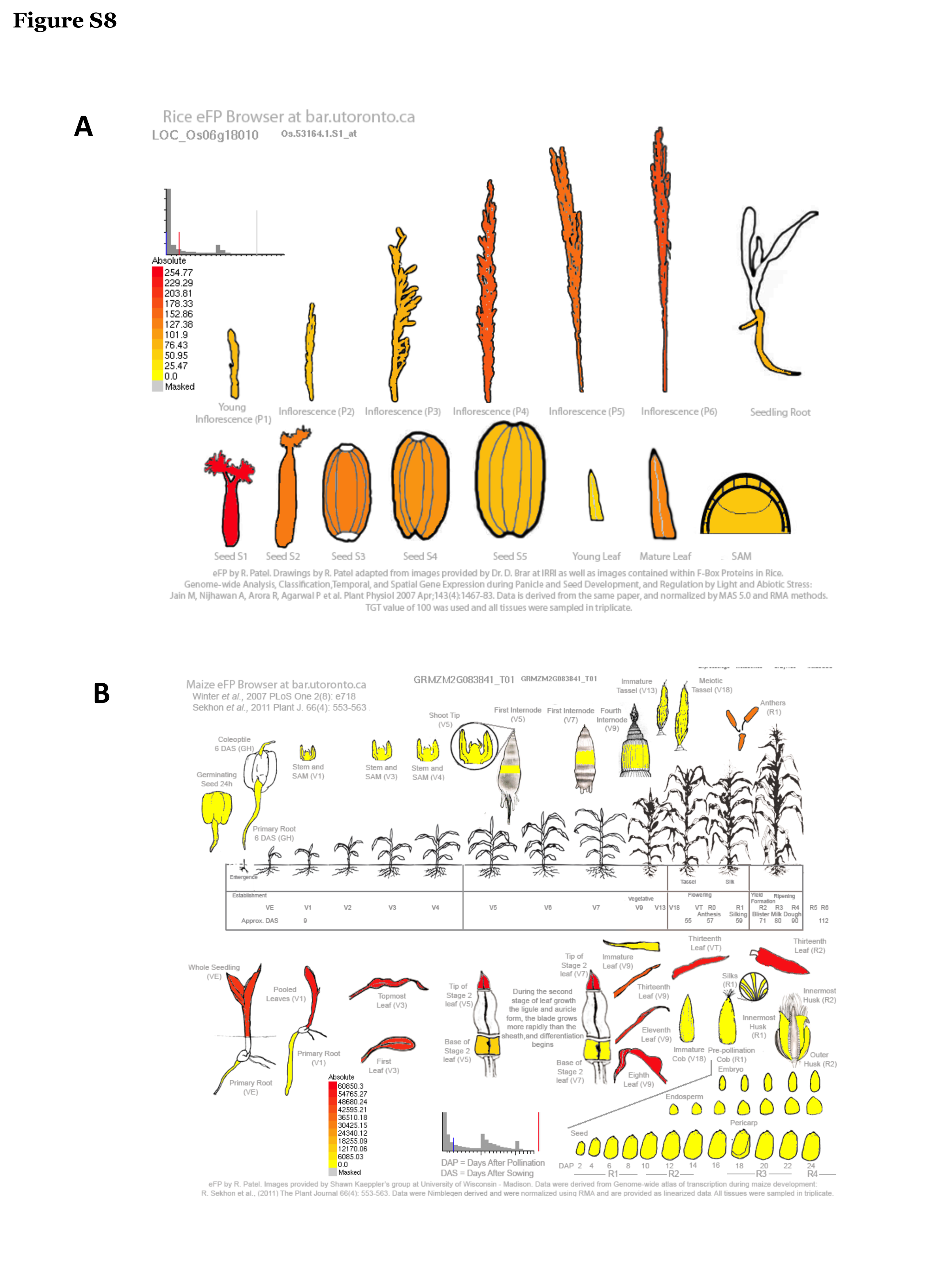

Supplement: S8 Fig — Expression analysis of (A) LOC_Os06g18010 (OsCGC) and (B) GRMZM2G083841 (ZmCGT) according to rice and maize eFP browser at bar.utoronto.ca. (TIF) [file pone.0198394.s013.tif]
