# Supplementary material for: Antinutritional factors in pearl millet grains: Phytate and goitrogens content variability and molecular characterization of genes involved in their pathways
Source: PLoS One. 2018 Jun 1;13(6):e0198394. doi: 10.1371/journal.pone.0198394 (PMC5983567; doi:10.1371/journal.pone.0198394)
Supplement: S3 Fig — Multiple alignment of the deduced amino acid sequences of genes coding for IPK1 from pearl millet (PglIPK1_284 and PglIPK1_475) and other plant species (names are as in S2 Table). (PDF) [file pone.0198394.s008.pdf]

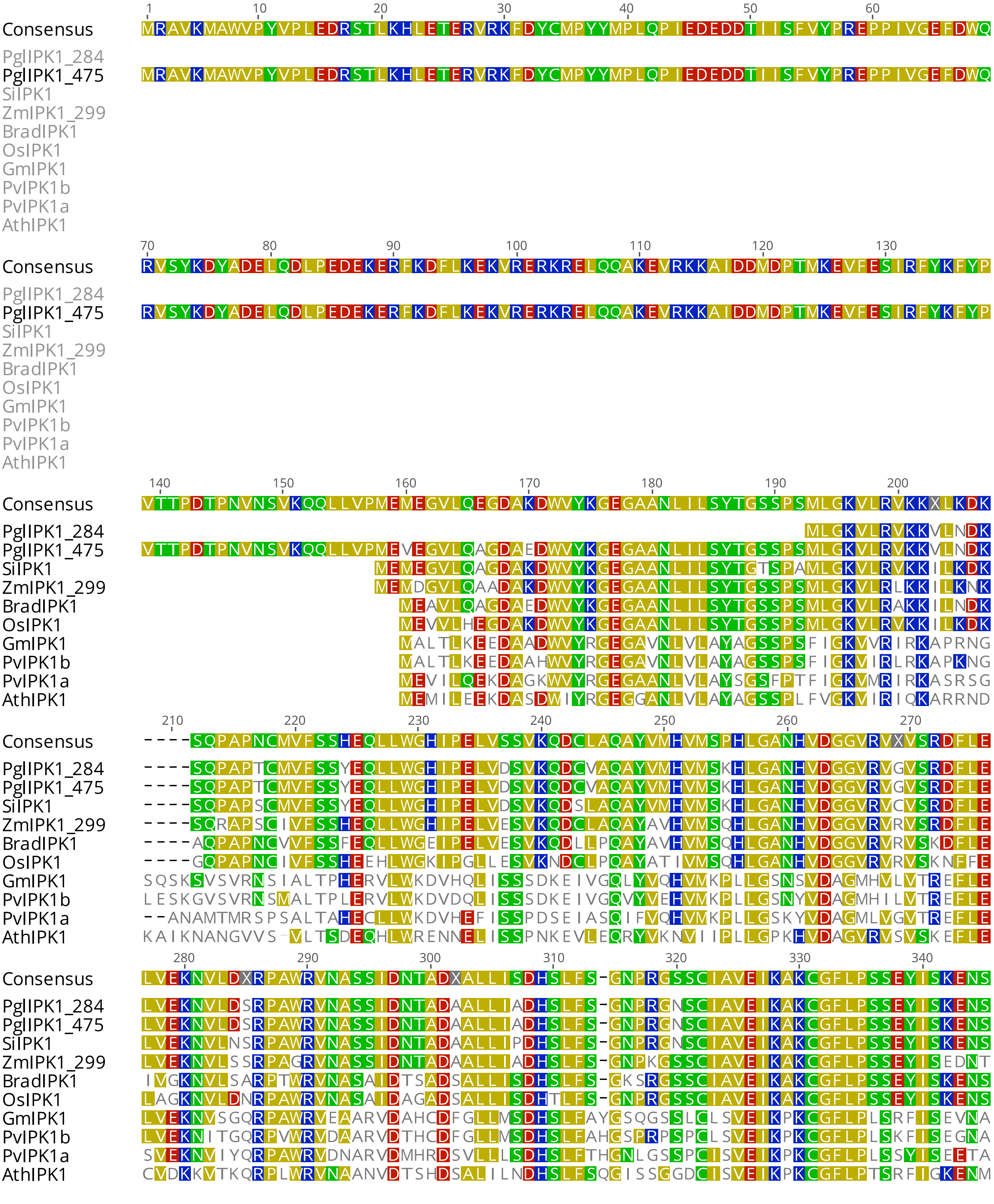
Consensus
PgIPK1_284
PgIPK1_475
SiIPK1
ZmIPK1_299
BradIPK1
OsIPK1
GmIPK1
PvIPK1b
PvIPK1a
AthIPK1

```
                 350       360       370       380       390       400       410
Consensus   IKKQVTRYKMHQHLKFHQGEISXTSEYNPLDLFSGSKERICXAIKSXFSTPQNNFRVFVNGSLVFGGMG

PgIPK1_284  IKKQVTRYKMHQHLKFHQGEISTTSEYNPLDLFSGSKERICIAIKSFFSTPQNNFRVFVNGSLVFGGMG
PgIPK1_475  IKKQVTRYKMHQHLKFHQGEISTTSEYNPLDLFSGSKERICIAIKSFFSTPQNNFRVFVNGSLVFGGMG
SiIPK1      IKKQVTRYKMHQHLKFHQGEISTPSEYNPLDLFSGSKERICIAIKSFFSTPQNNFRVFVNGSLVFGGMG
ZmIPK1_299  IKKQVTRYKMHQHLKFYQGEISKTSEYNPLDLFSGSKERICMAIKSLFSTPQNNLRIFVNGSLAFGGMG
BradIPK1    IKKQVTRYKMHQHLKFHQGEVSKTSEYDPLDLFSGSKERIHTAIKSFFSTPQNNFRIFVNGSLVFGGMG
OsIPK1      IKKQVTRYKMHQHLKFHLGEISKTSEYDPLDLFSGSKERIHMAIKSFFSTPQNNFRIFVDGSLVFGGMG
GmIPK1      IKRRITRFEMHQTLKLLQGEISQLSEYNPLDLFSGSKERILKAIKGLLTTPQNNFRVFLNGSLILGGLG
PvIPK1b     IKRRITRFEMHQTLKFLQGEISRLSEYNPLDLFSGSKERIKKAIKGLLTTPQNNFRVFLNGSLILGGLG
PvIPK1a     VKRTITRFQMHQVLKLQQGEISLLSEYNPLDLFSGSKEKTFKAIKNLFSSPQNNFRVFMNGSLIFGGLG
AthIPK1     LKTSVSRFKMHQLLKLEYNEISEESEYDPLDLFSGSKESVLEAIKALYSTPQNNFRVFLNGSLILGGSG

              420       430       440       450       460       470       480
Consensus   GGADNVHPAETKKCLEDLSKVS----GLXLPDFIELLSEAIFKSGVLXKLLATQKLDDHDIEGAIHLYY

PgIPK1_284  GGADNVHPAERNKCLEDLSKVS----GLELPDFIELLSEAIFRSGVLGKLLTTQKLDDHDIEGAIHLYY
PgIPK1_475  GGADNVHPAERNKCLEDLSKVS----GLELPDFIELLSEAIFRSGVLGKLLTTQKLDDHDIEGAIHLYY
SiIPK1      GGADNVHPAETDKCLQDLSKVS----GLELPDFIELLSEAIFRSGVLGKLLITQKLDDHDIEGAIHLYY
ZmIPK1_299  GGADSVHPADTLKCLEDLSKIS----GLKLPDFTELLSETIFRSEVLGNLLATQKLDDHDIEGVIHLYY
BradIPK1    GGADKVHPNETEKCLEDLSKVS----GLQLSDFIELLSEAIYKSGVLDKLLATQKLDDHDIEGAIHLYY
OsIPK1      GGADSVHPNETEKCLEDLSKVT----GLQLSDFIELLSEAIFKSGVLGKLLATQKLDDHDIEGAIHLYY
GmIPK1      GVAKNTDVCIAKAFEDELKSIIRADDDLCTNNLSTLVTEALQKSGVLDKLLKVQKLDNIDIEGVIHAYY
PvIPK1b     GVAKNTDICIAKEFEDELKSFIQVDDVLRTENLFTLVTEALQKSGVLDKLLEVQKLDSIDIEGVIHAYY
PvIPK1a     GGAQNTNICIAKAFEDALKSVIRSDDGLRTENLLTLVTEAVQKSGVLDQLLKVQKLDNVDIEGVIHAYY
AthIPK1     ESTGRTSPEIGYAFEDALKGFIQSEDGHRTECFLQLVSDAVYGSGVLDRLLEIQKLDKLDIEGAIHSYY

               490       500       510       520       530       540       550
Consensus   NIISQPCLVCKXLTDAELLRKYTXLHSLPLDKSLKIVRDFLISATAKDCSLMISFRPRESGXTDSEYDS

PgIPK1_284  NIISQPCLVCKKVTDAELLRKYTLLHSLPLDKSLKVVRDFLVSATAKDCSLMISFRPRESGTTDSEYDS
PgIPK1_475  NIISQPCLVCKKVTDAELLRKYTLLHSLPLDKSLKVVRDFLVSATAKDCSLMISFRPRESGTTDSEYDS
SiIPK1      SIISQPCLVCKNVTDAELLRKYTLLHSLPLDKSLKIVRDFLVSATAKDCSLMISFRPRESGTADSEYDS
ZmIPK1_299  NIISQPCLVCKNLTDVELLRKYTFLHSLPLDKSLKIVRDFLISATAKDCSLMISFRPRENGSTDSEYDS
BradIPK1    NITSQPCLVCKNISDAELLRKYAVLHSLSLDKSCKIIRDFLISATAKDCSLMMSFQPRECEKTDSEYDS
OsIPK1      NIISQPCLVCKSITDTELLRKYSTLHSLPLDKSEKIVRDFLISATAKDCSLMISFRPRQSGTTDSEYDS
GmIPK1      NITSQQCMVCKELS-EEQAKIYTPLHSASLDESLRIVKDYLIATTAKDCSLMLCFRPRNEEDSGSVYDN
PvIPK1b     NITSEQCVVCKELS-EEQAKTYASLHSASFDESLRIVKDYLIAATAKDCSLMLCFRPRNEEDSGSVYNN
PvIPK1a     DITHQQCMICKHLS-AEQLKRYTSLHSASLDESLRIVKDYLIATTAKDCSFMICFRPRKEGDSGSVCSN
AthIPK1     DLINQPCPICKEG---KPLEAELSLHALPLDESLKIVKEYLIAATAKDCSIMISFQSRNAWDSEPSGDY

                 560       570       580       590       600       610       620
Consensus   VFLDSVKQTYDYKAYFIDLDVKPLDKMEHYFKLDQKIVNFYXRNX-MVXGLKXS-XKGASXIXLQQXXX

PgIPK1_284  VFLDSVKQTYEYKANFIDLDVKPLDKMEHYFKLDQKIVNFYSRNE-IVPSLKSS-TKDASLIPLQQ
PgIPK1_475  VFLDSVKQTYEYKANFIDLDVKPLDKMEHYFKLDQKIVNFYSRNE-IVPSLKSS-TKDASLIPLQQ
SiIPK1      VFLDSVKQTYEYKANFIDLDVKPLDKMEHYFKLDQKIVNFYSRNEELVPSLKGSNTKDASQIQLQQ
ZmIPK1_299  VFLESVKRTYEYKAYFLDLDVKPLDKMEHYFKLDQRIVNFYTRNG---GGLAIS--KGQ*
BradIPK1    VFLESVNRIYDYKANFVDLDVKPLDKMVHYFKLDQKIVNFYTKHG-EVGRIACDSSKGGGSTDSTKVQP
OsIPK1      VFLDSVNQSYDYKAYFIDLDVKPLDKMVHYFKLDQKIVNFYTRNG-EVGGDPRDPPKGCGPR
GmIPK1      VYLDSTEQSFDYKVYFIDLDLKRLSKVEDYYELDKKIVNCYRQIIKMDQGRNEETGLKASNAAY*
PvIPK1b     VYLESTKQAFHFKVYFIDLDLKRLSKLKDYYELDKKIVSRYRQKIKMDQARNE--GLKACNASACYRDC
PvIPK1a     VYLHSTKQTFDFKVYFIDLDLKRMSKMEEYYELDKKIVSCYKEMVKMDRGRD----LRICKHHKLRT
AthIPK1     VSLKPTNQTFDYKVHFIDLSLKPLKRMESYYKLDKKIISFYNRKQKAENTAEQ---IGNSKPSHS

                   630       640       651
Consensus   XXXAVVADQTAAVTILKSPFQERHGGVLER

PgIPK1_284
PgIPK1_475
SiIPK1
ZmIPK1_299
BradIPK1    QH*
OsIPK1
GmIPK1
PvIPK1b     CSGAVVADQTAAVTILKSPFQERHGGVLER
PvIPK1a
AthIPK1
```
