# Supplementary material for: Antinutritional factors in pearl millet grains: Phytate and goitrogens content variability and molecular characterization of genes involved in their pathways
Source: PLoS One. 2018 Jun 1;13(6):e0198394. doi: 10.1371/journal.pone.0198394 (PMC5983567; doi:10.1371/journal.pone.0198394)
Supplement: S5 Fig — Multiple alignment of the deduced amino acid sequences of genes coding for MRP transporter from pearl millet (PglMRP) and other plant species (names are as in S2 Table). (PDF) [file pone.0198394.s010.pdf]

```
           1        10        20        30        40        50
Consensus  MAVDEIEILSXT-XFSXXSSGSXAPSFPXLLGLPALEL-AAICANLTLALLFLXVVSA

Zmlpa1     MPPSFPSLPLPEAVAA-TAHAALLALAALLLLLRAA
SbMRP      MPPSFPSLPLPEAAAA-AAHAALLALAALVLLLRAA
SiMRP      MPPSFPSLPLPEAAAA-AAHASLLALAALLLLLRAA
PgIMRP
OsMRP      MPHFPNLPLPEAAAA-AAHAALLALALLLLLLRSA
GmMRP1b    MAVDEIEILSPT-FSS---SGSFETLWSAILGLPLLEL-VAICANLTLSLLFLFVVSA
GmMRP1a    MAVDEIEILSST-LFSSSSSGSFEILWSAILGLPLLEL-VAICANLTLFILFLVVVSA
PvMRP1     MAVDEIGISSPTSFFSSYSSGSFESRWTAILGLPWVEV-VAICANLTLFIVFVFVLLA
PvMRP2     MGVARFFHDVLGLPVLEL-ATICVNLTLVLLFLFVVSV
GmMRP2     MGVARFFHDVLGLPVLEL-ATICMNLTFVLLFLFVVSV
AthMRP5    MDFIEISLIFREHLPLLEL-CSVIINLLLFLVFLFAVSA
AtABCC3    MDFLGSTTGSGTLAMLFSFSESILPLDS-RSFLLKPLFLRWLSGFLHS
AtABCC9    MFKPFGFAAETGSHLLTTQWLQLGNSLCLKERISIAMQVTFLA
AtABCC4    MWLLSSSPWLSELSCSYSAVVEHTSSVPVPIQWLRFVLLSPCPQRALFSAVDF
AtABCC1    MGFEPLDWYCKPVPNGVWTKTVDY-AFGAYTPCAIDSFVLGISH

           60        70        80        90        100       110
Consensus  RXLLVXCXSGLRFGKRRGS---GNASPGC---XXETRXVR-IGAWFKLSLLSCFYVL-

Zmlpa1     RALASRCASCLKAPRRRG---PAVVVGD--GAGGALAAATAGAWHRAVLASCAYAL-
SbMRP      RSLASRCASCLKAPRRRG---PAVVVGAGAGAGGALAATSAGAWHRAVLASCAYAL-
SiMRP      RALASRCASCLKAAPRRGP---AAAVAAG---GGTLAATAR-AWHRAVLASCAYAL-
PgIMRP
OsMRP      RALASRCASCLKTAPRR------AAAVDG----GLAAASSVGAWYRAALACCGYAL-
GmMRP1b    RKVLVCVGRGVRFGKENIT---GNASPGCVSVDLETRDVVR-IETWFKLSVLSCLYVL-
GmMRP1a    RKVLVCVWGGVRFGKENGT---GNASPGCVSVDLETRDIR-IETWFKLSVLSCFYVL-
PvMRP1     RRVVVCVGGGVRFGKDDGT---GNASRGCDSVDLETRDVR-IGTWFKWSVFSCFYVL-
PvMRP2     RRALVYQG-GFRFGKNGNS---GNASPICSVIDERRGVR-IGLVFKLSVVSCFYVL-
GmMRP2     RRVLVYGG-GFRFGKDGNS---GNASPICSVIDETRGVR-IGVGFKLSVLSCFYVL-
AthMRP5    RQILVCVRRGRDRLSKDDT---VSASN--LSLEREVNHVS-VGFGFNLSLLCCLYVL-
AtABCC3    VLLLVLFFSWVRKKIRGDS---GVTESLK-----DRRDFGFKSALFCSLALSLLNLV-
AtABCC9    FFLIHLALKWFGVVRNRGS---NDVEEDLK----KQSITVKQSFSYNISLLCSVSILG
AtABCC4    IFLLCFALHKLFSSPSSSSEINGHAEIRKPLIGIRGRTPTRTTAWFKTTVAVTVLLSF
AtABCC1    LVLLILCLYRLWLITKDHK-------------VDKFCLRSKWFSYFLALLAAYATAE

              120       130       140       150       160       170
Consensus  -LVQVLVLXFEGAXLGRGVS---------LXLLLXPAVQALAWAVLSFLALQCKFKAW

Zmlpa1     -LSQVAVLSYEVAVAGSRVS---------ARALLLPAVQAVSWAALLALALQARAVGW
SbMRP      -LAQVAVLSYEVAVAGSRVA---------AGALLLPAVQAVSWAALLALALQARAVGW
SiMRP      -LAQVAALSYEVAVAGSRVS---------AGALLLPAVQAVAWAALLVLALQARALGW
PgIMRP     LAW
OsMRP      -LAQVAALSYEVAVAGSHVA---------VEALLLPAVQALAWAALLALAMQARAVGW
GmMRP1b    -LVQVLLLGFDGVALIRGRD-LDVDLDLGLALLSVPLVQGLAWVVLSFSALQCKFKAS
GmMRP1a    -LVQVLVLGFDGVALIRGR--DLDLDLGLALLSVPLVQGLAWVVLSFSALQCKFKAC
PvMRP1     -LVQVLVFAFDGFALFRER--DVDLDWGLALLSAPLAQGLAWIALSFSALQCKFKAL
PvMRP2     -FVHVLALGFEGGALIWGED------DVDLSLLSVPAAQCLAWFVLSFWTLDCKFKVS
GmMRP2     -FVNVLALGFEGGALIWGEAN--GDADVDLSLLAVPAAQGLAWFVLSFSALYCKFKVS
AthMRP5    -GVQVLVLVYDGVKVRREVS--------DWFVLCFPASQSLAWFVLSFLVLHLKYKSS
AtABCC3    -LMSLSGFYWYESGWLDNEQ---------LVSSLGFLLGMVSWGVLSICLHRCRDCEH
AtABCC9    THCFILLLLFRDSVVSRCDS--------SVSVFSAEVSQSFSWLFVSVVVVKIRERRL
AtABCC4    CSVVLCVLAFTGKRRTQRPWN--------LIDPLFWLIHAVTHLVIAVLVLHQKRFAA
AtABCC1    PLFRLVMRISVLDLDGAGFP---------PYEAFMLVLEAFAWGSALVMTVVETKTYI

              180       190       200       210       220       230
Consensus  ERFPALLRVWWVVSFVLCLCILYVDGRXLWXXGSR--HLYXHMVANFASTPALGFLCL

Zmlpa1     ARFPALVRLWWVVSFALCVVIAYDDSRRLIGQGAR-AVDYAHMVANFASVPALGFLCL
SbMRP      ARFPALVRVWWVVSFALCVVIAYDDSRRLIGDGAR-AVDYAHMVANFASVPALGFLCL
SiMRP      ARFPALVRVWWVVSFALCVGIAYDDSRRLIRDEPR-TVDYAHMVANFASVPALGFLCL
PgIMRP     PRFPALVRVWWVVSFALCVGIAYDDSRRLVRDEPR-TVDYAHMVANFASAPALGFLCL
OsMRP      GRFPVLVRVWWVVSFVLCVGIAYDDTRHLMGDDDDDEVDYAHMVANFASAPALGFLCL
GmMRP1b    ERFPILLRLWWVMLFGICLCGLYVDGKGVWMEGSK--HLRSHVVANFTITPALAFLCI
GmMRP1a    ERFPVLLRVWLFVVFVICLCGLYVDGRGVWMEGSK--HLRSHVVANFAVTPALAFLCI
PvMRP1     ERFPILLRVWWFVLFVICLCGLYVDGRGVWMEGSK--HLRSHVVANFAVTPALGFLCI
PvMRP2     ERFPVLLRVWWFLCFVICLCTLYVDGRGFWENGSQ--HLCSRAVSNVAVTPPLAFLFV
GmMRP2     ERFPFLLRAWWFLSFVICLCTLYVDGRGFWEEGSE--HLCSRAVANVAVTPALAFLCV
AthMRP5    EKLPFLVRIWWFLAFSICLCTMYVDGRRLAIEGWS--RCSSHVANLAVTPALGFLCF
AtABCC3    KKAPFLLRLWLVFYLVVSCYSLVVDFVMYERRETVPVHLLVFDIVAFIAAVFLGYVAV
AtABCC9    VKFPWMLRSWWLCSFILSFS---FDAHFITAKHEP--LEFQDYADLTGLLASLFLLA
AtABCC4    LNHPLSLRIYWISSFVLTSLFAVTGIFHFLSDAAT--SLRAEDVASFFSFPLTAFLLI
AtABCC1    HELRWYVRFAVIYALVGDMVLLNLVLSVKEYYGSFKLYLYISEVAVQVAFGTLLFVYF
```

```
                 240       250       260       270       280       290
Consensus  VAVRGSTG--IEVTXXS-GLHEPLLVG--------EEPGCLRVTPYXDAGLFSLATLS

Zmlpa1     VGVMGSTGLELEFTEDGNGLHEPLLLG-RQRREAEEELGCLRVTPYADAGILSLATLS
SbMRP      VGVMGSTGLELEFMEDENGLHEPLLLG-RQRREAEEELGCLRVTPYSDAGILSLATLS
SiMRP      VGVMGSTGLEFEFT-DENGVHEPLLLG-RQRREAEEEPGCLRVTPYADAGILSLATLS
PglMRP     VGVMGSTGLEFEFTTGDDGVHEPLLLGGRQRREAEEEPGCLRVTPYADAGILSLATLS
OsMRP      VGVMGSTGVELEFTDDDSSVHEPLLLG-GQRRDADEEPGCLRVTPYGDAGIVSLATLS
GmMRP1b    VAIRGVTG--IKVFRNS-EEHQPLLVE--------EEPGCLKVTPYTDAGLFSLATLS
GmMRP1a    VAIRGVTG--IKVFRSS-EEQQPLLVD--------EDPGCLKVTPYSDAGLFSLAILS
PvMRP1     VAIRGVTG--IKVCRIS-EEQQPLLVE--------EEPGCLKVTPYNDAGLFSLATLS
PvMRP2     VAVRGGTG--IIVCRNS-DLQEPLLVE--------EEPGCLRVTPYLDAGLFSLATLS
GmMRP2     VAIRGGTG--IRVCGNS-DLQEPLLVD--------EEPGCLKVTPYRDAGLFSLATLS
AthMRP5    LAWRGVSG--IQVTRSSSDLQEPLLVE--------EEAACLKVTPYSTAGLVSLITLS
AtABCC3    LKKDRSNS--NGVLEEPLLNGGDSRVGGDDSVELNKTNGSGEATPYSRAGILSLLTFS
AtABCC9    VSIRGKTG--FHLLESS-GNTEPLLLG--DQTEQNKKDSYSSSSPYGNATLFQRITFS
AtABCC4    ASVRGITGLVTAETNSPTKPSDAVSVE-----------KSDNVSLYASASVFSKTFWL
AtABCC1    PNLDPYPGYTPVGTENSEDYEYEELPG------------GENICPERHANLFDSIFFS

                  300       310       320       330       340
Consensus  WLNPLLSIGAKRPLELKDIPLLAPKDRAKXCYKXLXSNWERXKAEN--PSREPSLAWA

Zmlpa1     WLSPLLSVGAQRPLELADIPLLAHKDRAKSCYKAMSAHYERQRLEY--PGREPSLTWA
SbMRP      WLSPLLSVGAQRPLELADIPLLAHKDRAKSCYKVMSAHYERQRLEH--PGREPSLTWA
SiMRP      WLSPLLSIGAQRPLELADIPLLAHKDRAKSCYKAMSAHYERQRLEN--PYREPSLTWA
PglMRP     WLSPLLSIGAQRPLELADIPLLAHKDRAKSCYKAMSAHYERQRLEN--PYREPSLTWA
OsMRP      WLSPLLSVGAQRPLELADIPLMAHKDRAKSCYKAMSSHYERQRMER--PGSEPSLAWA
GmMRP1b    WLNPLLSIGAKRPLELKDIPLVAAKDRSKTNYKVLNSNWERLKAEN--QSEQPSLAWA
GmMRP1a    WLNPLLSIGAKRPLELKDIPLVAPKDRSKTNYKVLNSNWERLKAEN--LSGQPSLAWA
PvMRP1     WLNPLLSIGAKRPLELKDIPLVAPNDRSKTNYKILNSNWEKLKAEN--TSRQPSLAWA
PvMRP2     WLNPLLSIGAKRPLELKDIPLVAPRDRAKTSYKILNSNWERLKAENDNPSKHSSLAWA
GmMRP2     WLNPLLSIGAKRPLELKDIPLVAPRDRAKTSYKVLNSNWERLKAENENPSKQPSLAWA
AthMRP5    WLDPLLSAGSKRPLELKDIPLLAPRDRAKSSYKVLKSNWKRCKSEN--PSKPPSLARA
AtABCC3    WMSPLIDIGNKKTLDLEDVPQLHDTDSVVGLAPKFRSMLESPDGGERSGVTTFKLIKA
AtABCC9    WINPLFSLGYKRPLEKDDVPDIDVKDSARFCSHAFDQKLKTTKEKE--GPGNAFFYNS
AtABCC4    WMNPLLSKGYKSPLTLEQVPTLSPEHKAERLALLFESSWPKPSENS-----SHPIRTT
AtABCC1    WLNPLMTLGSKRPLTEKDVWHLDTWDKTETLMRSFQKSWDKELEKP-----KPWLLRA

             350       360       370       380       390       400
Consensus  ILKSFWKEAAVNAXFAGVNTLVSYVGPYLISYFVDYLSGKE-XFPHEGYILAGIFFVA

Zmlpa1     ILKSFWREAAVNGTFAAVNTIVSYVGPYLISYFVDYLSGNI-AFPHEGYILASIFFVA
SbMRP      ILKSFWREAAVNGTFAAVNTIVSYVGPYLISYFVDYLSGNI-AFPHEGYILASIFFVA
SiMRP      ILKSFWREAAVNGAFAAVNTIVSYVGPYLISYFVDYLSGNI-AFPHEGYILASIFFVA
PglMRP     ILKSFWREAAVNGTFAAVNTIVSYVGPYLISYFVDYLSGNI-AFPHEGYILASVFFVA
OsMRP      ILKSFWREAAINGAFAAVNTIVSYVGPYLISYFVDYLSGKI-EFPHEGYILASVFFVA
GmMRP1b    LLKSFWKEAACNAVFAGVTTLVSYVGPYMISYFVDYLVGKE-IFPHEGYVLAGVFFVA
GmMRP1a    LLKSFWKEAACNAVFAGVTTLVSYVGPYMISYFVDYLVGKE-IFPHEGYVLAGVFFVA
PvMRP1     ILKSFWKEAACNAIFAGVTTLVSYVGPYMISYFVDFLVGKE-IFPHEGYVLAGIFFSA
PvMRP2     ILTSFWKEAALNAIFAGLNTLVSYVGPYMISYFVDYLSGKE-TFPHEGYALAGIFFAA
GmMRP2     ILKSFWKDAALNAIFAGMNTLVSYVGPYMISYFVDYLGGKE-TFPHEGYILAGIFFVA
AthMRP5    IMKSFWKEAACNAVFAGLNTLVSYVGPYLISYFVDYLGGKE-IFPHEGYVLAGIFFTS
AtABCC3    LYFTAQWEILVTAFFAFIYTVASYVGPALIDTFVQYLNGRR-QYNHEGYVLVITFFAA
AtABCC9    VLRYVWRKAAINAVFAVVNASTAYIGPYLINDFVEFLSEKQSQSLNHGYLLALGFLTA
AtABCC4    LLRCFWKEILFTAILAIVRLGVMYVGPVLIQSFVDFTSGKR-SSPWQGYYLVLILLVA
AtABCC1    LNNSLGGRFWWGGFWKIGNDCSQFVGPLLLNELLKSMQLNE--PAWIGYIYAISIFVG

              410       420       430       440       450       460
Consensus  KLVETLTTRQWYLGVDILGMHVRSALTAMVYRKGLRLSSSXRQSHTSGEIVNYMAVDV

Zmlpa1     KLLETLTARQWYLGVDIMGIHVKSGLTAMVYRKGLRLSNASRQSHTSGEIVNYMAVDV
SbMRP      KLLETLTARQWYLGVDIMGIHVKSGLTAMVYRKGLRLSNASRQSHTSGEIVNYMAVDV
SiMRP      KLLETLTARQWYLGVDIMGIHVKSGLTAMVYRKGLRLSNASRQSHTSGEIVNYMAVDV
PglMRP     KLLETLTARQWYLGVDIMGIHVKSGLTAMVYRKGLRLSNSSRQSHTSGEIVNYMAVDV
OsMRP      KLLETLTARQWYLGVDVMGIHVKSGLTAMVYRKGLRLSNSSRQSHTSGEIVNYMAVDV
GmMRP1b    KLVETFTTRQWYLGVDILGMHVRSALTAMVYRKGLRISSLAKQSHTSGEVVNYMAIDV
GmMRP1a    KLVETFTTRQWYLGVDILGMHVRSALTAMVYRKGLRISSLAKQSHTSGEVVNYMAIDV
PvMRP1     KLVETFTTRQWYIGVDIMGMHVRSALTAMVYRKGLRISSLAKQSHTSGEIVNYMAIDV
PvMRP2     KLVETVTTRQWYLGVDILGMHVRSALTAMVYRKGLRLSSSAKQSHTSGEIVNYMAVDV
GmMRP2     KLVETVTTRQWYLGVDILGMHVRSALTAMVYRKGLRLSSSAKQSHTSGEIVNYMAVDV
AthMRP5    KLIETVTTRQWYMGVDILGMHVRSALTAMVYRKGLKLSSIAKQNHTSGEIVNYMAVDV
AtABCC3    KIVECLSQRHWFFRLQKVGIRMRSALVAMIYEKGLTLSCQSKQGRTSGEIINFMTVDA
AtABCC9    KIVETVTQRQWIFGARQLGLRLRAALISHIYQKGLVLSSQSRQSHTSGEIINYMSVDV
AtABCC4    KFVEVLTTHQFNFDSQKLGMLIRSTLITALYKKGLKLTGSARQNHGVGQIVNYMAVDA
AtABCC1    VVLGVLCEAQYFQNVMRVGYRLRSALIAAVFRKSLRLTNEGRKKFQTGKITNLMTTDA
```

```
                470       480       490       500       510       520
Consensus  QRVGDYSWYLHDIWMLPLQIXLALAILYKNVGIASVATLVATXJSIAVTVPVAKLQEH

Zmlpa1     QRVGDYAWYFHDIWMLPLQIILALAILYKNVGIAMVSTLVATVLSIAASVPVAKLQEH
SbMRP      QRVGDYAWYFHDIWMLPLQIILALAILYKNVGIAMVSTLVATALSIAASVPVAKLQEH
SiMRP      QRVGDYAWYFHDIWMLPLQIILALAILYKNVGIAMVSTLIATVLSIAASVPVAKLQEH
PglMRP     QRVGDYAWYFHDIWMLPLQIILALAILYKNVGIAMVSTLVATVLSIAASVPVAKLQEH
OsMRP      QRVGDYAWYFHDIWMLPLQIILALAILYKNVGIAMVSTLVATVLSIAASVPVAKLQEH
GmMRP1b    QRVGDYSWYLHDMWMLPLQIVLALAILYKNVGIAAIATLIATIISIVVTVPIARVQEN
GmMRP1a    QRVGDYSWYLHDMWMLPLQIVLALAILYKNVGIASIATLIATIISIAVTVPIARIQEN
PvMRP1     QRVGDYSWYLHDMWMLPLQIVLALAILYKNIGIASVATLIATIISIIVTVPVARIQED
PvMRP2     QRVGDFSWYLHDLWMLPMQIVLALLILYKNIGIASIATLVATVVSIVVTIPVAKIQED
GmMRP2     QRVGDYSWYLHDMWMLPMQIVLALLILYKNVGIASVATLIATIISIVVTVPVARVQED
AthMRP5    QRIGDYSWYLHDIWMLPMQIVLALAILYKSVGIAAVATLVATIISILVTIPLAKVQED
AtABCC3    ERIGNFSWYMHDPWMVLLQVGLALWILYRNLGLASIAALVATIIVMLINFPFGRMQER
AtABCC9    QRITDFIWYVNNIWMLPIQIFSAIYILQKHLGLGALAALVTTLMVMACNYPLTRLQRN
AtABCC4    QQLSDMMLQLHAIWLMPLQVTVALVLLYGSLGASVITAVIGLTGVFVFILLGTQRNNG
AtABCC1    ESLQQICQSLHTMWSAPFRIIVALVLLYQQLGVASIIGALFLVLMFPIQTVIISKTQK

                530       540       550       560       570       580
Consensus  YQDKLMAAKDERMRKTSECLRNMRILKLQAWEDRYRLKLEEMRXVEFKWLRKALYSQA

Zmlpa1     YQDKLMASKDERMRKTSECLKNMRILKLQAWEDRYRLQLEEMRNVECRWLRWALYSQA
SbMRP      YQDKLMASKDERMRKTSECLKNMRILKLQAWEDRYRLQLEEMRNVECRWLRWALYSQA
SiMRP      YQDKLMASKDERMRKTSECLKNMRILKLQAWEDRYRLQLETMRNVECRWLRWALYSQA
PglMRP     YQDKLMASKDERMRKTSECLKNMRILKLQAWEDRYRLQLEEMRNVECRWLRWALYSQA
OsMRP      YQDKLMASKDERMRKTSECLKNMRILKLQAWEDRYRLKLEEMRNVECKWLRWALYSQA
GmMRP1b    YQDKLMAAKDERMRKTSECLRNMRILKLQAWEDRYRVKLEEMRGVEFKWLRKALYSQA
GmMRP1a    YQDKLMAAKDERMRKTSECLRNMRILKLQAWEDRYRVKLEEMRGVEFKWLRKALYSQA
PvMRP1     YQDRLMAAKDERMRKTSECLRNMRILKLQAWEDRYRVMLEDMRGVEFKWLRKALYSQA
PvMRP2     YQDNLMAAKDERMRKTSECLRNMRILKLQAWEDRYRLKLEEMRGVEFKWLRKSLYTQA
GmMRP2     YQDKLMAAKDERMRKTSECLRNMRILKLQAWEDRYRLKLEEMRGVEFKWLRKALYSQA
AthMRP5    YQDKLMTAKDERMRKTSECLRNMRVLKLQAWEDRYRVRLEEMREEEYGWLRKALYSQA
AtABCC3    FQEKLMEAKDSRMKSTSEILRNMRILKLQGWEMKFLSKIFDLRKSEEGWLKKYVYNSA
AtABCC9    YQSDIMNAKDDRMKATSEILKNMKILKLQAWDNQFLNKVKTLRKKEYDCLWKSLRLQA
AtABCC4    YQFSLMGNRDSRMKATNEMLNYMRVIKFQAWENHFNKRILKFRDMEFGWLSKFLYSIA
AtABCC1    LTKEGLQRTDKRIGLMNEVLAAMDTVKCYAWENSFQSKVQTVRDDELSWFRKAQLLSA

                 590       600       610       620       630
Consensus  FXTFVFWSSPIFVSVVTFGTCILLGGQLTAGGVLSALATFRILQEPLRNFPDLISMMA

Zmlpa1     AVTFVFWSSPIFVAVITFGTCILLGGQLTAGGVLSALATFRILQEPLRNFPDLISMMA
SbMRP      AVTFVFWSSPIFVAVITFGTCILLGGQLTAGGVLSALATFRILQEPLRNFPDLISMMA
SiMRP      AVTFVFWSSPIFVSVITFGTCILLGGQLTAGGVLSALATFRILQEPLRNFPDLISMMA
PglMRP     AVTFVFWSSPIFVAVITFGTCILLGGQLTAGGVLSALATFRILQEPLRNFPDLISMMA
OsMRP      AVTFVFWSSPIFVAVITFGTCILLGGELTAGGVLSALATFRILQEPLRNFPDLISMIA
GmMRP1b    FITFIFWSSPIFVSAVTFATSILLGGQLTAGGVLSALATFRILQEPLRNFPDLVSTMA
GmMRP1a    FITFIFWSSPIFVSAVTFGTSILLGGQLTAGGVLSALATFRILQEPLRNFPDLVSTMA
PvMRP1     FITFMFWSSPIFVSAVTFATSILLGGQLTAGGVLSALATFRILQEPLRNFPDLVSTMA
PvMRP2     FITFIFWSSPIFVSAVTFATCILLGGQLTAGGVLSALATFRILQEPLRNFPDLVSTMA
GmMRP2     CITFMFWSSPIFVSAVTFATSILLGGQLTAGGVLSALATFRILQEPLRNFPDLVSTMA
AthMRP5    FVTFIFWSSPIFVAAVTFATSIFLGTQLTAGGVLSALATFRILQEPLRNFPDLVSMMA
AtABCC3    VISFVFWGAPTLVSVSTFGACILLGIPLESGKILSALATFRILQEPIYNLPDTISMIV
AtABCC9    FTTFILWGAPSLISVVTFVTCMLMGVKLTAGAVLSALATFQMLQSPIFGLPDLLSALV
AtABCC4    GNIIVLWSTPVLISALTFATALALGVKLDAGTVFTTTTIFKILQEPIRTFPQSMISLS
AtABCC1    FNMFILNSIPVLVTVVSFGVFSLLGGDLTPARAFTSLSLFSVLRFPLFMLPNIITQMV

             640       650       660       670       680       690
Consensus  QTKVSLDRLSHFLQQEELQEDATIVVPQGSTNKAIEIKDGVFSWDPSSS-RPTLSGIH

Zmlpa1     QTRVSLDRLSHFLQQEELPDDATINVPQSSTDKAVDIKDGAFSWNPYTL-TPTLSDIH
SbMRP      QTRVSLDRLSHFLQQEELPDDATINVPQSSTDKAIDIKNGAFSWNPYSL-TPTLSDIQ
SiMRP      QTRVSLDRLSHFLQQEELPDDATINVPQSSTDKAIDIKDGTFSWNPYSP-TPTLSGIH
PglMRP     QTRVSLDRLSHFLQQEELPDDATINVPQSSTDKAIDIKDGTFSWNPYSQ-TPTLSDIH
OsMRP      QTRVSLDRLSHFLQQEELPDDATITVPHGSTDKAININDATFSWNPSSP-TPTLSGIN
GmMRP1b    QTKVSLDRLSGFLLEEELQEDATIVLPQGITNIAIEIKDGIFCWDPSSSFRPTLSGIS
GmMRP1a    QTKVSLDRLSGFLLEEELQEDATIVLPQGITNIAIEIKGGVFCWDPSSSSRPTLSGIS
PvMRP1     QTKVSLDRLSGFLLEEELQEDATVAMPQGITNIALEIKDGVFCWDPLSS-RPTLSGIS
PvMRP2     QTRVSLDRITTYLQDEELQEDATIVMPRGISNMAIEIRDGVFCWATSLP-RPTLSGIH
GmMRP2     QTKVSLDRISAFLQDEELQEDATIVLPPGISNTAIEIMDGVFCWDSSLP-RPTLSGIH
AthMRP5    QTKVSLDRISGFLQEEELQEDATVVIPRGLSNIAIEIKDGVFCWDPFSS-RPTLSGIQ
AtABCC3    QTKVSLDRLASYLCLDNLQPDIVERLPKGSSDVAVEVINSTLSWDVSSS-NPTLKDIN
AtABCC9    QSKVSADRIASYLQQSETQKDAVEYCSKDHTELSVEIENGAFSWEPESS-RPTLDDIE
AtABCC4    QAMISLGRLDSYMMSKELSEDAVERALGCDGNTAVEVRDGSFSWDDEDN-EPALSDIN
AtABCC1    NANVSLNRLEEVLSTEERVLLPNPPIEPGQP--AISIRNGYFSWDSKAD-RPTLSNIN
```

```
                 700       710       720       730       740       750
Consensus  LKVERGMRVAVCGXVGSGKSSLLSSILGEIPKLSG-EVRVCGSAAYVPQSAWIQSGNI

Zmlpa1     LSVVRGMRVAVCGVIGSGKSSLLSSILGEIPKLCG-HVRISGTAAYVPQTAWIQSGNI
SbMRP      LSVVRGMRVAVCGVIGSGKSSLLSSILGEIPKLCG-HVRISGTAAYVPQTAWIQSGNI
SiMRP      LSVVRSMRVAVCGVIGSGKSSLLSSILGEIPKLCG-HVRISGTAAYVPQTAWIQSGNI
PglMRP     LSVVRGMRVAVCGVIGSGKSSLLSSILGEIPKLCG-HVRISGTAAYVPQTAWIQSGNI
OsMRP      LSVVRGMRVAVCGVIGSGKSSLLSSILGEIPKLCG-QVRISGSAAYVPQTAWIQSGNI
GmMRP1b    MKVERRMRVAVCGMVGSGKSSFLSCILGEIPKLSG-EVRVCGSSAYVSQSAWIQSGTI
GmMRP1a    MKVERRMRVAVCGMVGSGKSSFLLCILGEIPKISG-EVRVCGSSAYVSQSAWIQSGTI
PvMRP1     MKVEKRMRVAVCGMVGSGKSSFLSCILGEIPKTSG-EVRVCGSSAYVSQSAWIQSGTI
PvMRP2     MKVEKGMNVAVCGMVGSGKSSFLSCILGEIPKLSG-EVKVCGSVAYVSQSAWIQSGNI
GmMRP2     VKVERGMTVAVCGMVGSGKSSFLSCILGEIPKLSG-EVKMCGSVAYVSQSAWIQSGNI
AthMRP5    MKVEKGMRVAVCGTVGSGKSSFISCILGEIPKISG-EVRICGTTGYVSQSAWIQSGNI
AtABCC3    FKVFPGMKVAVCGTVGSGKSSLLSSLLGEVPKVSG-SLKVCGTKAYVAQSPWIQSGKI
AtABCC9    LKVKSGMKVAVCGAVGSGKSSLLSSILGEIQKLKG-TVRVSGKQAYVPQSPWILSGTI
AtABCC4    FKVKKGELTAIVGTVGSGKSSLLASVLGEMHRISG-QVRVCGSTGYVAQTSWIENGTV
AtABCC1    LDIPLGSLVAVVGSTGEGKTSLISAMLGELPARSDATVTLRGSVAYVPQVSWIFNATV

                760       770       780       790       800       810
Consensus  EENILFGSPMDKXKYKRVJHACSLKKDLELXSHGDQTIIGDRGINLSGGQKQRVQLAR

Zmlpa1     EENILFGSQMDRQRYKRVIAACCLKKDLELLQYGDQTVIGDRGINLSGGQKQRVQLAR
SbMRP      EENILFGSPMDRQRYKRVIAACCLKKDLELLQYGDQTVIGDRGINLSGGQKQRVQLAR
SiMRP      EENILFGSPMDRQRYKRVIAACSLKKDLELLQYGDQTIIGDRGINLSGGQKQRVQLAR
PglMRP     EENILFGSPMDRQRYKRVIAACSLKKDLELLQYGDQTIIGDRGINLSGGQKQRVQLAR
OsMRP      EENILFGSPMDKQRYKRVIEACSLKKDLQLLQYGDQTIIGDRGINLSGGQKQRVQLAR
GmMRP1b    EENILFGSPMDKAKYKNVLHACSLKKDLELFSHGDQTIIGDRGINLSGGQKQRVQLAR
GmMRP1a    EENILFGSPMDKAKYKNVLHACSLKKDLELFSHGDLTIIGDRGINLSGGQKQRVQLAR
PvMRP1     EENILFGSPMDKAKYKNVLHACSLKKDLELFSHGDQTIIGDRGINLSGGQKQRVQLAR
PvMRP2     EENILFGTPMDKAKYKKVLHACSLKKDLELFSHGDQTIIGDRGINLSGGQKQRVQLAR
GmMRP2     EENILFGTPMDKAKYKNVLHACSLKKDLELFSHGDQTIIGDRGINLSGGQKQRVQLAR
AthMRP5    EENILFGSPMEKTKYKNVIQACSLKKDIELFSHGDQTIIGERGINLSGGQKQRVQLAR
AtABCC3    EDNILFGKPMERERYDKVLEACSLSKDLEILSFGDQTVIGERGINLSGGQKQRIQIAR
AtABCC9    RDNILFGSMYESEKYERTVKACALIKDFELFSNGDLTEIGERGINMSGGQKQRIQIAR
AtABCC4    QDNILFGLPMVREKYNKVLNVCSLEKDLQMMEFGDKTEIGERGINLSGGQKQRIQLAR
AtABCC1    RDNILFGAPFDQEKYERVIDVTALQHDLELLPGGDLTEIGERGVNISGGQKQRVSMAR

                   820       830       840       850       860       870
Consensus  ALYQDADIYLLDDPFSAVDAHTGSELFREYILTALADKTVIYVTHQVEFLPAADLILV

Zmlpa1     ALYQDADIYLLDDPFSAVDAHTGSELFKEYILTALATKTVIYVTHQVEFLPAADLILV
SbMRP      ALYQDADIYLLDDPFSAVDAHTGSELFKEYILSALATKTVIYVTHQVEFLPAADLILV
SiMRP      ALYQDADIYLLDDPFSAVDAHTGSELFKEYILSALATKTVIYVTHQVEFLPAADLILV
PglMRP     ALYQDSDIYLLDDPFSAVDAHTGSELFKEYILSALATKTVIYVTHQVEFLPAADLILV
OsMRP      ALYQDADIYLLDDPFSAVDAHTGSELFREYILTALASKTVIYVTHQIEFLPAADLILV
GmMRP1b    ALYQDADIYLLDDPFSAVDAHTGSDLFREYILTALADKTVIFVTHQVEFLPAADLILV
GmMRP1a    ALYQDADIYLLDDPFSAVDAHTGSDLFREYILTALADKTVIYVTHQVEFLPAADLILV
PvMRP1     ALYQDADIYLLDDPFSAVDAHTGSDLFRDYILTALADKTVIYVTHQVEFLPAADLILV
PvMRP2     ALYQDAEIYLLDDPFSAVDAHTGSELFREYVLTALADKTVIFVTHQVEFLPSADMILV
GmMRP2     ALYQDADIYLLDDPFSAVDAHTGSELFREYVLTALADKTVIFVTHQVEFLPAADMIMV
AthMRP5    ALYQDADIYLLDDPFSALDAHTGSDLFRDYILSALAEKTVVFVTHQVEFLPAADLILV
AtABCC3    ALYQDADIYLFDDPFSAVDAHTGSHLFKEVLLGLLCSKSVIYVTHQVEFLPAADLILV
AtABCC9    AVYQNADIYLLDDPFSAVDAHTGRELFEDCLMGILKDKTVLYVTHQVEFLPAADLILV
AtABCC4    AVYQECDVYLLDDVFSAVDAHTGSDIFKKCVRGALKGKTVLLVTHQVDFLHNVDCILV
AtABCC1    AVYSNSDVCILDDPLSALDAHVGQQVFEKCIKRELGQTTRVLVTNQLHFLSQVDKILL

                       880       890       900       910       920
Consensus  LKXGHIIQAGKYDDLLQAGTDFNALVSAHXEAIEAMDIPEDS--DSD----XVSSXMX

Zmlpa1     LKDGHITQAGKYDDLLQAGTDFNALVSAHKEAIETMDIFEDS--DSD----TVSS-IP
SbMRP      LKDGHITQAGKYDDLLQAGTDFNALVSAHKEAIETMDIFEDS--DGD----TVSS-IP
SiMRP      LKDGHITQAGKYDDLLQAGTDFNALVSAHKEAIETMDIFEDS--DGD----TVSSSIP
PglMRP     LKDGHITQAGKYDDLLQAGTDFNALVSAHKEAIETMDIFEDS--DGD----TVSSSIP
OsMRP      LKDGHITQAGKYDDLLQAGTDFNALVCAHKEAIETMEFSEDS--DED----TVSS-VP
GmMRP1b    LKEGCIIQSGKYDDLLQAGTDFNTLVSAHHEAIEAMDIPTHSSEESDENLSLEASVMT
GmMRP1a    LKEGCIIQSGKYDDLLQAGTDFNTLVSAHNEAIEAMDIPTHS-EDSDENLSLEACVMT
PvMRP1     LREGCIIQAGKYDDLLQAGTDFNILVSAHHEAIEAMDIPTHSSEDSDENLSLEASVMT
PvMRP2     LKEGHIIQAGKYDDLFLAGTDFKTLVSAHHEAIEAMDIPNHS-EDSDENVPLDESIMK
GmMRP2     LKEGHIIQAGKYDDLLQAGTDFKTLVSAHHEAIEAMDIPNHS-EDSDENVPLDDTIMT
AthMRP5    LKEGRIIQSGKYDDLLQAGTDFKALVSAHHEAIEAMDIPSPSSEDSDENPIRDSLVLH
AtABCC3    MKDGRISQAGKYNDILNSGTDFMELIGAHQEALAVVDS-------------VDANSVS
AtABCC9    MQNGRVMQAGKFEELLKQNIGFEVLVGAHNEALDSILSIEKS---------------S
AtABCC4    MRDGKIVESGKYDELVSSGLDFGELVAAHETSMELVEAGADS-----------AAVAT
AtABCC1    VHEGTVKEEGTYEELCHSGPLFQRLMENAGKVEDYSEENGEA----------------
```

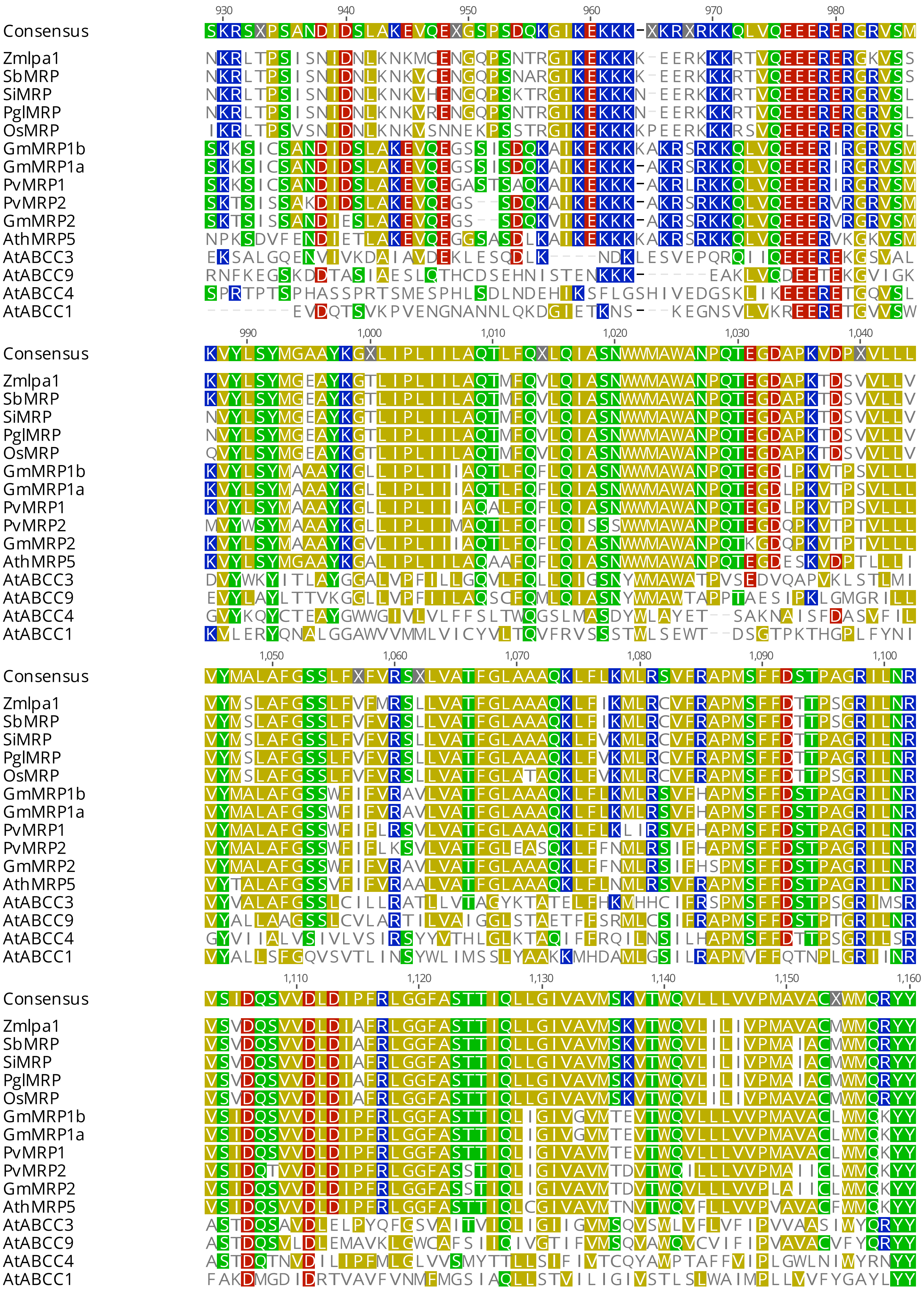
930 940 950 960 970 980
Consensus SKRSXPSANDIDSLAKEVQEXGSPSDQKGIKEKKK-XKRXRKKQLVQEEERERGRVSM
Zmlpa1 NKRLTPSISNIDNLKNKMCENGQPSNTRGIKEKKKK-EERKKKRTVQEEERERGKVSS
SbMRP NKRLTPSISNIDNLKNKVCENGQPSNARGIKEKKKK-EERKKKRTVQEEERERGRVSS
SiMRP NKRLTPSISNIDNLKNKVHENGQPSKTRGIKEKKKN-EERKKKRTVQEEERERGRVSL
PglMRP NKRLTPSISNIDNLKNKVRENGQPSNTRGIKEKKKN-EERKKKRTVQEEERERGRVSL
OsMRP IKRLTPSVSNIDNLKNKVSNNEKPSSTRGIKEKKKKPEERKKKRSVQEEERERGRVSL
GmMRP1b SKKSICSANDIDSLAKEVQEGSSISDQKAIKEKKKAKRSRKKQLVQEEERIRGRVSM
GmMRP1a SKKSICSANDIDSLAKEVQEGSSISDQKAIKEKK-AKRSRKKQLVQEEERIRGRVSM
PvMRP1 SKKSICSANDIDSLAKEVQEGASTSAQKAIKEKK-AKRLRKKQLVQEEERIRGRVSM
PvMRP2 SKTSISSAKDIDSLAKEVQEGS--SDQKAIKEKK-AKRSRKKQLVQEEERVRGRVSM
GmMRP2 SKTSISSANDIESLAKEVQEGS--SDQKVIKEKK-AKRSRKKQLVQEEERVRGRVSM
AthMRP5 NPKSDVFENDIETLAKEVQEGGSASDLKAIKEKKKAKRSRKKQLVQEEERVKGKVSM
AtABCC3 EKSALGQENVIVKDAIAVDEKLESQDLK----NDKLESVEPQRQIIQEEEREKGSVAL
AtABCC9 RNFKEGSKDDTASIAESLQTHCDSEHNISTENKKK------EAKLVQDEETEKGVIGK
AtABCC4 SPRTPTSPHASSPRTSMESPHLSDLNDEHIKSFLGSHIVEDGSKLIKEEERETGQVSL
AtABCC1 -------EVDQTSVKPVENGNANNLQKDGIETKNS---KEGNSVLVKREERETGVVSW
990 1,000 1,010 1,020 1,030 1,040
Consensus KVYLSYMGAAYKGXLIPLIILAQTLFQXLQIASNWWMAWANPQTEGDAPKVDPXVLLL
Zmlpa1 KVYLSYMGEAYKGTLIPLIILAQTMFQVLQIASNWWMAWANPQTEGDAPKTDSVVLLV
SbMRP KVYLSYMGEAYKGTLIPLIILAQTMFQVLQIASNWWMAWANPQTEGDAPKTDSVVLLV
SiMRP NVYLSYMGEAYKGTLIPLIILAQTMFQVLQIASNWWMAWANPQTEGDAPKTDSVVLLV
PglMRP NVYLSYMGEAYKGTLIPLIILAQTMFQVLQIASNWWMAWANPQTEGDAPKTDSVVLLV
OsMRP QVYLSYMGEAYKGTLIPLIILAQTMFQVLQIASNWWMAWANPQTEGDAPKTDSVVLLV
GmMRP1b KVYLSYMAAAYKGLLIPLIIIAQTLFQFLQIASNWWMAWANPQTEGDLPKVTPSVLLL
GmMRP1a KVYLSYMAAAYKGLLIPLIIIAQTLFQFLQIASNWWMAWANPQTEGDLPKVTPSVLLL
PvMRP1 KVYLSYMAAAYKGLLIPLIIIAQALFQFLQIASNWWMAWANPQTEGDLPKVTPSVLLL
PvMRP2 MVYWSYMAAAYKGLLIPLIIMAQTLFQFLQISSSWWMAWANPQTEGDQPKVTPTVLLL
GmMRP2 KVYLSYMAAAYKGVLIPLIIIAQTLFQFLQIASNWWMAWANPQTKGDQPKVTPTVLLL
AthMRP5 KVYLSYMGAAYKGALIPLIILAQAAFQFLQIASNWWMAWANPQTEGDESKVDPTLLLI
AtABCC3 DVYWKYITLAYGGALVPFILLGQVLFQLLQIGSNYWMAWATPVSEDVQAPVKLSTLMI
AtABCC9 EVYLAYLTTVKGGLLVPFIILAQSCFQMLQIASNYWMAWTAPPTAESIPKLGMGRILL
AtABCC4 GVYKQYCTEAYGWWGIVLVLFFSLTWQGSLMASDYWLAYET--SAKNAISFDASVFIL
AtABCC1 KVLERYQNALGGAWVVMMLVICYVLTQVFRVSSSTWLSEWT--DSGTPKTHGPLFYNI
1,050 1,060 1,070 1,080 1,090 1,100
Consensus VYMALAFGSSLFXFVRSXLVATFGLAAAQKLFLKMLRSVFRAPMSFFDSTPAGRILNR
Zmlpa1 VYMSLAFGSSLFVFMRSLLVATFGLAAAQKLFIKMLRCVFRAPMSFFDTTPSGRILNR
SbMRP VYMSLAFGSSLFVFVRSLLVATFGLAAAQKLFIKMLRCVFRAPMSFFDTTPSGRILNR
SiMRP VYMSLAFGSSLFVFVRSLLVATFGLAAAQKLFVKMLRCVFRAPMSFFDTTPAGRILNR
PglMRP VYMSLAFGSSLFVFVRSLLVATFGLAAAQKLFVKMLRCVFRAPMSFFDTTPAGRILNR
OsMRP VYMSLAFGSSLFVFVRSLLVATFGLATAQKLFVKMLRCVFRAPMSFFDTTPSGRILNR
GmMRP1b VYMALAFGSSWFIFVRAVLVATFGLAAAQKLFLKMLRSVFHAPMSFFDSTPAGRILNR
GmMRP1a VYMALAFGSSWFIFVRAVLVATFGLAAAQKLFLKMLRSVFHAPMSFFDSTPAGRILNR
PvMRP1 VYMALAFGSSWFIFLRSVLVATFGLAAAQKLFLKLIRSVFHAPMSFFDSTPAGRILNR
PvMRP2 VYMALAFGSSWFIFLKSVLVATFGLEASQKLFFNMLRSIFHAPMSFFDSTPAGRILNR
GmMRP2 VYMALAFGSSWFIFVRAVLVATFGLAAAQKLFFNMLRSIFHSPMSFFDSTPAGRILNR
AthMRP5 VYTALAFGSSVFIFVRAALVATFGLAAAQKLFLNMLRSVFRAPMSFFDSTPAGRILNR
AtABCC3 VYVALAFGSSLCILLRATLLVTAGYKTATELFHKMHHCIFRSPMSFFDSTPSGRIMSR
AtABCC9 VYALLAAGSSLCVLARTILVAIGGLSTAETFFSRMLCSIFRAPMSFFDSTPTGRILNR
AtABCC4 GYVIIALVSIVLVSIRSYYVTHLGLKTAQIFFRQILNSILHAPMSFFDTTPSGRILSR
AtABCC1 VYALLSFGQVSVTLINSYWLIMSSLYAAKKMHDAMLGSILRAPMVFFQTNPLGRIINR
1,110 1,120 1,130 1,140 1,150 1,160
Consensus VSIDQSVVDLDIPFRLGGFASTTIQLLGIVAVMSKVTWQVLLVVPMAVACXWMQRYY
Zmlpa1 VSVDQSVVDLDIAFRLGGFASTTIQLLGIVAVMSKVTWQVLILIVPMAVACMWMQRYY
SbMRP VSVDQSVVDLDIAFRLGGFASTTIQLLGIVAVMSKVTWQVLILIVPMAIACMWMQRYY
SiMRP VSVDQSVVDLDIAFRLGGFASTTIQLLGIVAVMSKVTWQVLILIVPMAIACMWMQRYY
PglMRP VSVDQSVVDLDIAFRLGGFASTTIQLLGIVAVMSKVTWQVLILIVPMAIACMWMQRYY
OsMRP VSVDQSVVDLDIAFRLGGFASTTIQLLGIVAVMSKVTWQVLILIVPMAVACMWMQRYY
GmMRP1b VSIDQSVVDLDIPFRLGGFASTTIQLIGIVGVMTEVTWQVLLVVPMAVACLWMQKYY
GmMRP1a VSIDQSVVDLDIPFRLGGFASTTIQLIGIVGVMTEVTWQVLLVVPMAVACLWMQKYY
PvMRP1 VSIDQSVVDLDIPFRLGGFASTTIQLIGIVAVMTEVTWQVLLVVPMAVACLWMQKYY
PvMRP2 VSIDQTVVDLDIPFRLGGFASSTIQLIGIVAVMTDVTWQILLVVPMAIICLWMQKYY
GmMRP2 VSIDQSVVDLDIPFRLGGFASSTIQLIGIVAVMTDVTWQVLLVVPLAIICLWMQKYY
AthMRP5 VSIDQSVVDLDIPFRLGGFASTTIQLCGIVAVMTNVTWQVFLLVVPVAVACFWMQKYY
AtABCC3 ASTDQSAVDLELPYQFGSVAITVIQLIGIIGVMSQVSWLVFLVFIPVVAASIWYQRYY
AtABCC9 ASTDQSVLDLEMAVKLGWCAFSIIQIVGTIFVMSQVAWQVCVIFIPVAVACVFYQRYY
AtABCC4 ASTDQTNVDILIPFMLGLVVSMYTTLLSIFIVTCQYAWPTAFFVIPLGWLNIWYRNYY
AtABCC1 FAKDMGDIDRTVAVFVNMFMGSIAQLLSTVILIGIVSTLSLWAIMPLLVVFYGAYLYY

```
            1,170     1,180     1,190     1,200     1,210
Consensus   XASSRELXRIVSXQKSPIIHLFXESIAGAATIRGFGQEKRFMKRNLYLLDCFARPFFC

Zmlpa1      IASSRELTRILSVQKSPVIHLFSESIAGAATIRGFGQEKRFMKRNLYLLDCFARPLFS
SbMRP       IASSRELTRILSVQKSPVIHLFSESIAGAATIRGFGQEKRFMKRNLYLLDCFARPLFS
SiMRP       IASSRELTRILSVQKSPVIHLFSESIAGAATIRGFAQEKRFMKRNLYLLDCFARPLFS
PglMRP      IASSRELTRILSVQKSPVIHLFSESIAGAATIRGFGQEKRFMKRNLYLLDCFARPLFS
OsMRP       IASSRELTRILSVQKSPVIHLFSESIAGAATIRGFGQEKRFMKRNLYLLDCFARPLFS
GmMRP1b     MASSRELVRIVSIQKSPIIHLFGESIAGASTIRGFGQEKRFMKRNLYLLDCFARPFFC
GmMRP1a     MASSRELVRIVSIQKSPIIHLFGESIAGASTIRGFGQEKRFMKRNLYLLDCFARPFFC
PvMRP1      MASSRELVRIVSIQKSPIIHLFGESIAGASTIRGFGQEKRFMKRNLYLLDCFARPFFC
PvMRP2      MASSRELVRIVSIQKSPIIHLFGESIAGAATIRGFGQEKRFMKRNLYLLDCFARPFFC
GmMRP2      MASSRELVRIVSIQKSPIIHLFGESIAGAATIRGFGQEKRFMKRNLYLLDCFARPFFC
AthMRP5     MASSRELVRIVSIQKSPIIHLFGESIAGAATIRGFGQEKRFIKRNLYLLDCFVRPFFC
AtABCC3     IAAARELSRLVGVCKAPLIQHFSETISGATTIRSFSQEFRFRSDNMRLSDGYSRPKFY
AtABCC9     TPTARELSRMSGVERAPILHHFAESLAGATTIRAFDQRDRFISSNLVLIDSHSRPWFH
AtABCC4     LASSRELTRMDSITKAPIIHHFSESIAGVMTIRSFRKQELFRQENVKRVNDNLRMDFH
AtABCC1     QNTSREIKRMDSTTRSPVYAQFGEALNGLSSIRAYKAYDRMAEINGRSMDNNIRFTLV

            1,220     1,230     1,240     1,250     1,260     1,270
Consensus   SLAAIEWLCLRMELLSTFVFAFCMVLLVS----FPPGTIDPSMAGLAVTYGLNLNARL

Zmlpa1      SLAAIEWLCLRMELLSTFVFAFCMAILVS----FPPGTIEPSMAGLAVTYGLNLNARM
SbMRP       SLAAIEWLCLRMELLSTFVFAFCMAILVS----FPPGTIEPSMAGLAVTYGLNLNARM
SiMRP       SLAAIEWLCLRMELLSTFVFAFCMAILVS----FPPGTIEPSMAGLAVTYGLNLNARM
PglMRP      SLAAIEWLCLRMELLSTFVFAFCMAILVS----FPPGTIEPSMAGLAVTYGLNLNARM
OsMRP       SLAAIEWLCLRMELLSTFVFAFCMAILVS----FPPGTIEPSMAGLAVTYGLNLNARM
GmMRP1b     SLSAIEWLCLRMELLSTFVFAFCMVLLVS----FPRGSIDPSMAGLAVTYGLNLNARL
GmMRP1a     SLSAIEWLCLRMELLSTFVFAFCMVLLVS----FPRGSIDPSMAGLAVTYGLNLNARL
PvMRP1      SLSAIEWLCLRMELLSTFVFAFCMVLLVS----FPRGTIDPSMAGLAVTYGLNLNARL
PvMRP2      SLAAIEWLCLRMELLSTFVFAFCLVLLVS----LPHGSIDPSMAGLAVTYGLNLNARL
GmMRP2      SLAAIEWLCLRMELLSTFVFAFCLVLLVS----LPHGSIDPSMAGLAVTYGLNLNARL
AthMRP5     SIAAIEWLCLRMELLSTLVFAFCMVLLVS----FPHGTIDPSMAGLAVTYGLNLNGRL
AtABCC3     TAGAMEWLCFRLDMLSSLTFVFSLVFLVS----IPTGVIDPSLAGLAVTYGLSLNTLQ
AtABCC9     VASAMEWLSFRLNLLSHFVFAFSLVLLVT----LPEGVINPSIAGLGVTYGLSLNVLQ
AtABCC4     NNGSNEWLGFRLELVGSWVLCISALFMVL----LPSNVIRPENVGLSLSYGLSLNSVL
AtABCC1     NMAANRWLGIRLEVLGGLMVWLTASLAVMQNGKAANQQAYASTMGLLLSYALSITSSL

            1,280     1,290     1,300     1,310     1,320     1,330
Consensus   SRWILSFCKLENKIISVERIYQYSQIPSEAPLXIEBSRPPSSWPENGTIELIDLKVRY

Zmlpa1      SRWILSFCKLENRIISVERIYQYCRLPSEAPLIIENCRPPSSWPQNGNIELIDLKVRY
SbMRP       SRWILSFCKLENRIISVERIYQYCKLPSEAPLIIENCRPPSSWPHNGSIELIDLKVRY
SiMRP       SRWILSFCKLENRIISVERIYQYCKIPSEAPLVIENCRPQSSWPENGNIELIDLKVRY
PglMRP      SRWILSFCKLENRIISVERIYQYCKIPSEAPLVIENCRPQSSWPENGNIELIDLKVRY
OsMRP       SRWILSFCKLENRIISVERIYQYCKLPSEAPLIIENSRPSSSWPENGNIELVDLKVRY
GmMRP1b     SRWILSFCKLENKIISIERIYQYSQIPSEAPTIIEDSRPPFSWPENGTIEIIDLKVRY
GmMRP1a     SRWILSFCKLENKIISIERIYQYSQIPSEAPTVIEDYRPPSSWPENGTIEIIDLKIRY
PvMRP1      SRWILSFCKLENKIISIERIYQYSQIPREAPTIIEDSRPPSSWPENGTIEIIDLKVRY
PvMRP2      SRWILSFCKLENKIISIERIYQYSQIPCEAPAVIEDSRPPSSWPESGTIQLIDLKVRY
GmMRP2      SRWILSFCKLENKIISIERIYQYSQIPSEAPAIVEDSRPPSSWPENGTIQLIDLKVRY
AthMRP5     SRWILSFCKLENKIISIERIYQYSQIVGEAPAIIEDFRPPSSWPATGTIELVDVKVRY
AtABCC3     AWLIWTLCNLENKIISVERILQYASVPSEPPLVIESNRPEQSWPSRGEVEIRDLQVRY
AtABCC9     ATVIWNICNAENKMISVERILQYSKIPSEAPLVIDGHRPLDNWPNVGSIVFRDLQVRY
AtABCC4     FFAIYMSCFVENKMVSVERIKQFTDIPSESEWERKETLPPSNWPFHGNVHLEDLKVRY
AtABCC1     TAVLRLASLAENSLNSVERVGNYIEIPSEAPLVIENNRPPPGWPSSGSIKFEDVVLRY

            1,340     1,350     1,360     1,370     1,380     1,390
Consensus   KENLPLVLHGVSCTFPGGKKIGIVGRTGSGKSTLIQALFRLIEPTGGKIIIDNIDISA

Zmlpa1      KDDLPLVLHGVSCMFPGGKKIGIVGRTGSGKSTLIQALFRLIEPTGGKIIIDNIDISA
SbMRP       KDDLPLVLHGVSCMFPGGKKIGIVGRTGSGKSTLIQALFRLIEPTGGKIIIDDIDISA
SiMRP       KDDLPLVLHGVSCMFPGGKKIGIVGRTGSGKSTLIQALFRLIEPTGGKIIIDNIDISA
PglMRP      KDDLPLVLHGVSCIFPGGKKVGIVGRTGSGKSTLIQALFRLIEPTGGKIIIDNIDISA
OsMRP       KDDLPLVLHGISCIFPGGKKIGIVGRTGSGKSTLIQALFRLIEPTGGKVIIDDVDISR
GmMRP1b     KENLPMVLHGVTCTFPGGKKIGIVGRTGSGKSTLIQALFRLIEPASGSILIDNINISE
GmMRP1a     KENLPLVLYGVTCTFPGGKKIGIVGRTGSGKSTLIQALFRLIEPTSGSILIDNINISE
PvMRP1      KENLPLVLHGVTCTFPGGKKIGIVGRTGSGKSTLIQALFRLIEPTSGSILIDNINISE
PvMRP2      KENLPVVLHGVSCIFPGGKKIGIVGRTGSGKSTLIQALFRLVEPEAGSIFIDNINISD
GmMRP2      KENLPVVLHGVSCTFPGGKKIGIVGRTGSGKSTLIQALFRLVEPEAGSILIDNINISS
AthMRP5     AENLPTVLHGVSCVFPGGKKIGIVGRTGSGKSTLIQALFRLIEPTAGKITIDNIDISQ
AtABCC3     APHMPLVLRGITCTFKGGLRTGIVGRTGSGKSTLIQTLFRIVEPSAGEIRIDGVNILT
AtABCC9     AEHFPAVLKNITCEFPGGKKIGVVGRTGSGKSTLIQALFRIVEPSQGTIVIDNVDITK
AtABCC4     RPNTPLVLKGITLDIKGGEKVGVVGRTGSGKSTLIQVLFRLVEPSGGKIIIDGIDIST
AtABCC1     RPELPPVLHGVSFLISPMDKVGIVGRTGAGKSSLLNALFRIVELEKGRILIDECDIGR
```

```
            1,400     1,410     1,420     1,430     1,440     1,450
Consensus   IGLHDLRSRLSIIPQDPTLFEGTIRXNLDPLEEHSDKEIWEALDKCQLGEVIRSKEEK

Zmlpa1      IGLHDLRSRLSIIPQDPTLFEGTIRMNLDPLEECTDQEIWEALEKCQLGEVIRSKEEK
SbMRP       IGLHDLRSRLSIIPQDPTLFEGTIRMNLDPLEECADQEIWEALEKCQLGEVIRSKEEK
SiMRP       IGLHDLRSRLSIIPQDPTLFEGTIRMNLDPLEERADHEIWEALEKCQLGEVIRSKEEK
PglMRP      IGLHDLRSRLSIIPQDPTLFEGTIRMNLDPLEERADHEIWEALEKCQLGEVIRSKEEK
OsMRP       IGLHDLRSRLSIIPQDPTLFEGTIRMNLDPLEECTDQEIWEALEKCQLGEVIRSKDEK
GmMRP1b     IGLHDLRSHLSIIPQDPTLFEGTIRGNLDPLDEHSDKEIWEALDKSQLGEVIREKGQQ
GmMRP1a     IGLHDLRSHLSIIPQDPTLFEGTIRGNLDPLDEHSDKEIWEALDKSQLGEVIREKGQQ
PvMRP1      IGLHDLRGHLSIIPQDPTLFEGTIRGNLDPLEEHSDKEIWEALDKSQLGEVIRDKGQQ
PvMRP2      IGLHDLRSHLSIIPQDPTLFEGTIRGNLDPLEEHSDKEIWEALDKSQLSQIIRETERK
GmMRP2      IGLHDLRSHLSIIPQDPTLFEGTIRGNLDPLDEHSDKEIWEALDKSQLGDIIRETERK
AthMRP5     IGLHDLRSRLGIIPQDPTLFEGTIRANLDPLEEHSDDKIWEALDKSQLGDVVRGKDLK
AtABCC3     IGLHDLRLRLSIIPQDPTMFEGTMRSNLDPLEEYTDDQIWEALDKCQLGDEVRKKEQK
AtABCC9     IGLHDLRSRLGIIPQDPALFDGTIRLNLDPLAQYTDHEIWEAIDKCQLGDVIRAKDER
AtABCC4     LGLHDLRSRFGIIPQEPVLFEGTVRSNIDPTEQYSDEEIWKSLERCQLKDVVATKPEK
AtABCC1     FGLMDLRKVLGIIPQAPVLFSGTVRFNLDPFSEHNDADLWESLERAHLKDTIRRNPLG

            1,460     1,470     1,480     1,490     1,500
Consensus   LDSPVLENGDNWSVGQRQLVALGRALLKQSKILVLDEATASVDTATDNLIQKIIRSEF

Zmlpa1      LDSPVLENGDNWSVGQRQLIALGRALLKQAKILVLDEATASVDTATDNLIQKIIRSEF
SbMRP       LDSPVLENGDNWSVGQRQLIALGRALLKQAKILVLDEATASVDTATDNLIQKIIRSEF
SiMRP       LDSPVLENGDNWSVGQRQLIALGRALLKQAKILVLDEATASVDTATDNLIQKIIRSEF
PglMRP      LDSPVLENGDNWSVGQRQLIALGRALLKQAKILVLDEATASVDTATDNLIQKIIRSEF
OsMRP       LDSPVLENGDNWSVGQRQLIALGRALLKQAKILVLDEATASVDTATDNLIQKIIRSEF
GmMRP1b     LDTPVLENGDNWSVGQRQLVALGRALLQQSRILVLDEATASVDTATDNLIQKIIRSEF
GmMRP1a     LDTPVLENGDNWSVGQRQLVALGRALLQQSRILVLDEATASVDTATDNLIQKIIRSEF
PvMRP1      LDTPVLENGDNWSVGQRQLVALGRALLQQSRILVLDEATASVDTATDNLIQKIIRSEF
PvMRP2      LDMPVLENGDNWSVGQRQLVSLGRALLKQSKILVLDEATASVDTATDNLIQKIIRREF
GmMRP2      LDMPVLENGDNWSVGQCQLVSLGRALLKQSKILVLDEATASVDTATDNLIQKIIRREF
AthMRP5     LDSPVLENGDNWSVGQRQLVSLGRALLKQAKILVLDEATASVDTATDNLIQKIIRTEF
AtABCC3     LDSSVSENGDNWSMGQRQLVCLGRVLLKRSKILVLDEATASVDTATDNLIQKTLREHF
AtABCC9     LDATVVENGENWSVGQRQLVCLGRVLLKKSNILVLDEATASVDSATDGVIQKIINQEF
AtABCC4     LDSLVVDNGENWSVGQRQLLCLGRVMLKRSRLLFLDEATASVDSQTDAVIQKIIREDF
AtABCC1     LDAEVTEAGENFSVGQRQLLSLARALLRRSKILVLDEATAAVDVRTDVLIQKTIREEF

            1,510     1,520     1,530     1,540     1,550     1,560
Consensus   KDCTVCTIAHRIPTVIDSDLVLVLSDGKVAEFDTPXRLLEDKSSMFLKLVTEYSSRSS

Zmlpa1      KDCTVCTIAHRIPTVIDSDLVLVLSDGKIAEFDTPQRLLEDKSSMFIQLVSEYSTRSS
SbMRP       KDCTVCTIAHRIPTVIDSDLVLVLSDGKIAEFDTPQKLLEDKSSMFMQLVSEYSTRSS
SiMRP       KDCTVCTIAHRIPTVIDSDLVLVLSDGKVAEFDTPQRLLEDKSSMFMQLVSEYSTRSS
PglMRP      KDCTVCTIAHRIPTVIDSDLVLVLSDGKVAEFDTPQKLLEDKSSMFMQLVSEYSTRSS
OsMRP       KDCTVCTIAHRIPTVIDSDLVLVLSDGKIAEFDTPQRLLEDKSSMFMQLVSEYSTRSS
GmMRP1b     KDCTVCTIAHRIPTVIDSDLVLVLSDGLVAEFDTPSRLLEDKSSVFLKLVTEYSSRSS
GmMRP1a     KECTVCTIAHRIPTVIDSDLVLVLSDGRVAEFNTPSRLLEDKSSMFLKLVTEYSSRSS
PvMRP1      KNCTVCTIAHRIPTVIDSDQVLVLSDGRVAEFDTPSRLLEDKSSMFLKLVTEYSSRSS
PvMRP2      RDCTVCTIAHRIPTVIDSDLVMVLSDGRVAEFDTPSRLLEDKSSMFLKLVTEYSSRSS
GmMRP2      RDCTVCTIAHRIPTVIDSDLVLVLSDGRVAEFDSPSRLLEDKSSMFLKLVTEYSSRSS
AthMRP5     EDCTVCTIAHRIPTVIDSDLVLVLSDGRVAEFDTPARLLEDKSSMFLKLVTEYSSRST
AtABCC3     SDCTVITIAHRISSVIDSDMVLLLSNGIIEEYDTPVRLLEDKSSSFSKLVAEYTSRSS
AtABCC9     KDRTVVTIAHRIHTVIESDLVLVLSDGRIAEFDSPAKLLQREDSFFSKLIKEYSLRSN
AtABCC4     ASCTIISIAHRIPTVMDGDRVLVIDAGKAKEFDSPARLLE-RPSLFAALVQEYALRSA
AtABCC1     KSCTMLIIAHRLNTIIDCDKVLVLDSGKVQEFSSPENLLSNGESSFSKMVQSTGTANA

              1,570     1,580     1,590     1,600     1,610     1,620
Consensus   GIPDF*XLXXKRTREANGDDSQPLEGQRKWQASSRWAAAAQFALAVSLTSSHNDLQSL

Zmlpa1      CI
SbMRP       CI*
SiMRP       CI*
PglMRP      CI
OsMRP       CI*
GmMRP1b     GIPDF*
GmMRP1a     GIPDF*
PvMRP1      GIPEF
PvMRP2      GIPDF*
GmMRP2      GIPDF*
AthMRP5     GIPEL
AtABCC3     SSFD
AtABCC9     HFAGSNDLLS
AtABCC4     GI
AtABCC1     EYLRSITLENKRTREANGDDSQPLEGQRKWQASSRWAAAAQFALAVSLTSSHNDLQSL
```

```
                 1,630     1,640     1,650     1,660     1,670     1,680
Consensus    EIEDDNSILKKTKDAVVTLRSVLEGKHDKEIEDSLNQSDISRERWWPSLYKMVEGLAV

Zmlpa1
SbMRP
SiMRP
PglMRP
OsMRP
GmMRP1b
GmMRP1a
PvMRP1
PvMRP2
GmMRP2
AthMRP5
AtABCC3
AtABCC9
AtABCC4
AtABCC1      EIEDDNSILKKTKDAVVTLRSVLEGKHDKEIEDSLNQSDISRERWWPSLYKMVEGLAV

                     1,690     1,700     1,710
Consensus    MSRLARNRMQHPDYNLEGKSFDWDNVEM

Zmlpa1
SbMRP
SiMRP
PglMRP
OsMRP
GmMRP1b
GmMRP1a
PvMRP1
PvMRP2
GmMRP2
AthMRP5
AtABCC3
AtABCC9
AtABCC4
AtABCC1      MSRLARNRMQHPDYNLEGKSFDWDNVEM
```
