# Supplementary material for: Antinutritional factors in pearl millet grains: Phytate and goitrogens content variability and molecular characterization of genes involved in their pathways
Source: PLoS One. 2018 Jun 1;13(6):e0198394. doi: 10.1371/journal.pone.0198394 (PMC5983567; doi:10.1371/journal.pone.0198394)
Supplement: S6 Fig — Multiple alignment of the deduced amino acid sequences of genes coding for CGT from pearl millet (PglCGT_646 and PglCGT_648) and other plant species (names are as in S2 Table). Red arrow indicates the start codon according to the CDS of PglCGT_646 and PglCGT_648 as reported in the annotated pearl millet genome. Blue dots indicate conserved amino acid residues within the N-terminal acceptor binding pocket. (PDF) [file pone.0198394.s011.pdf]

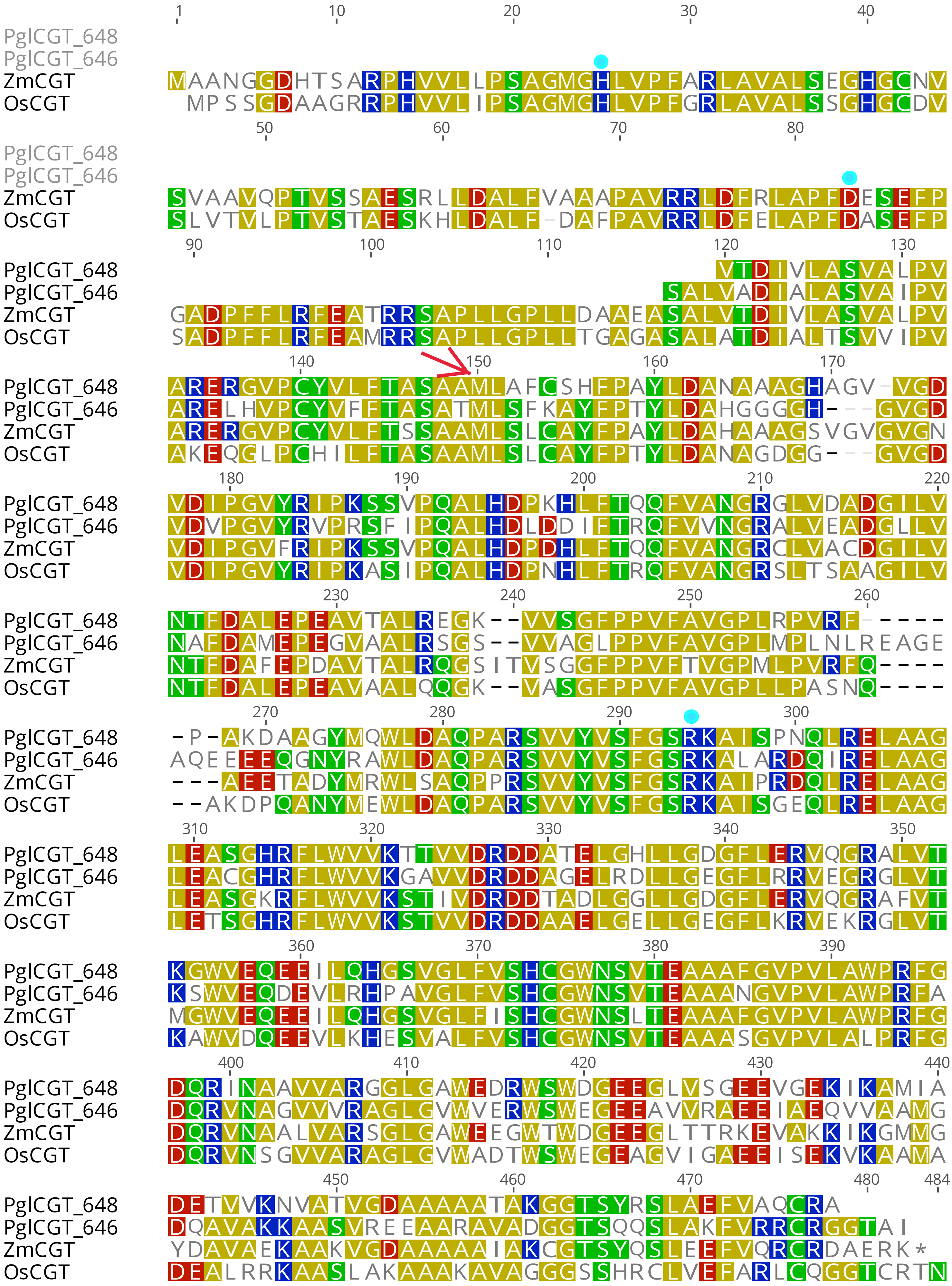
1 10 20 30 40
PgICGT_648
PgICGT_646
ZmCGT MAANGGDHTSARPHVVLLPSAGMGHLVPFARLAVALSEGHGCNV
OsCGT MPSSGDAAGRRPHVVLIPSAGMGHLVPFGRLAVALSSGHGCDV
50 60 70 80
PgICGT_648
PgICGT_646
ZmCGT SVAAVQPTVSSAESRLLDALFVAAAPAVRRLDFRLAPFDESEFP
OsCGT SLVTVLPTVSTAESKHLDALF-DAFPAVRRLDFELAPFDASEFP
90 100 110 120 130
PgICGT_648 VTDIVLASVALPV
PgICGT_646 SALVADIALASVAIPV
ZmCGT GADPFFLRFEATRRSAPLLGPLLDAAEASALVTDIVLASVALPV
OsCGT SADPFFLRFEAMRRSAPLLGPLLTGAGASALATDIALTSVVIPV
140 150 160 170
PgICGT_648 ARERGVPCYVLFTASAAMLAFCSHFPAYLDANAAAGHAGV-VGD
PgICGT_646 ARELHVPCYVFFTASATMLSFKAYFPTYLDAHGGGGH---GVGD
ZmCGT ARERGVPCYVLFTSSAAMLSLCAYFPAYLDAHAAAGSVGVGVGN
OsCGT AKEQGLPCHILFTASAAMLSLCAYFPTYLDANAGDGG---GVGD
180 190 200 210 220
PgICGT_648 VDIPGVYRIPKSSVPQALHDPKHLFTQQFVANGRGLVDADGILV
PgICGT_646 VDVPGVYRVPRSFIPQALHDLDDIFTRQFVVNGRALVEADGLLV
ZmCGT VDIPGVFRIPKSSVPQALHDPDHLFTQQFVANGRCLVACDGILV
OsCGT VDIPGVYRIPKASIPQALHDPNHLFTRQFVANGRSLTSAAGILV
230 240 250 260
PgICGT_648 NTFDALEPEAVTALREGK--VVSGFPPVFAVGPLRPVRF-----
PgICGT_646 NAFDAMEPEGVAALRSGS--VVAGLPPVFAVGPLMPLNLREAGE
ZmCGT NTFDAFEPDAVTALRQGSITVSGGFPPVFTVGPMLPVRFQ----
OsCGT NTFDALEPEAVAALQQGK--VASGFPPVFAVGPLLPASNQ----
270 280 290 300
PgICGT_648 -P-AKDAAGYMQWLDAQPARSVVYVSFGSRKAISPNQLRELAAG
PgICGT_646 AQEEEEQGNYRAWLDAQPARSVVYVSFGSRKALARDQIRELAAG
ZmCGT ---AEETADYMRWLSAQPPRSVVYVSFGSRKAIPRDQLRELAAG
OsCGT --AKDPQANYMEWLDAQPARSVVYVSFGSRKAISGEQLRELAAG
310 320 330 340 350
PgICGT_648 LEASGHRFLWVVKTTVVDRDDATELGHLLGDGFLERVQGRALVT
PgICGT_646 LEACGHRFLWVVKGAVVDRDDAGELRDLLGEGFLRRVEGRGLVT
ZmCGT LEASGKRFLWVVKSTIVDRDDTADLGGLLGDGFLERVQGRAFVT
OsCGT LETSGHRFLWVVKSTVVDRDDAAELGELLGEGFLKRVEKRGLVT
360 370 380 390
PgICGT_648 KGWVEQEEILQHGSVGLFVSHCGWNSVTEAAAFGVPVLAWPRFG
PgICGT_646 KSWVEQDEVLRHPAVGLFVSHCGWNSVTEAAANGVPVLAWPRFA
ZmCGT MGWVEQEEILQHGSVGLFISHCGWNSLTEAAAFGVPVLAWPRFG
OsCGT KAWVDQEEVLKHESVALFVSHCGWNSVTEAAASGVPVLALPRFG
400 410 420 430 440
PgICGT_648 DQRINAAVVARGGLGAWEDRWSWDGEEGLVSGEEVGEKIKAMIA
PgICGT_646 DQRVNAGVVVRAGLGVWVERWSWEGEEAVVRAEEIAEQVVAAMG
ZmCGT DQRVNAALVARSGLGAWEEGWTWDGEEGLTTRKEVAKKIKGMMG
OsCGT DQRVNSGVVARAGLGVWADTWSWEGEAGVIGAEEISEKVKAAMA
450 460 470 480 484
PgICGT_648 DETVVKNVATVGDAAAAATAKGGTSYRSLAEFVAQCRA
PgICGT_646 DQAVAKKAASVREEAARAVADGGTSQQSLAKFVRRCRGGTAI
ZmCGT YDAVAEKAAKVGDAAAAAIAKCGTSYQSLEEFVQRCRDAERK*
OsCGT DEALRRKAASLAKAAAKAVAGGGSSHRCLVEFARLCQGGTCRTN
